# Desirable Pore Connectivity Effects in Multiscale Interactive Cobalt Centers with Asymmetric B/N‐Coordination Carbon for Promoting Zn‐Air Batteries

**DOI:** 10.1002/advs.202514619

**Published:** 2025-10-16

**Authors:** Tingzheng Fu, Hongbiao Xiao, Qiusen Liu, Ye Yu, Zhiqing Che, Yixing Zhang, Anran Chen, Mian Li, Tingting Liu

**Affiliations:** ^1^ School of Materials and Energy Yunnan University Kunming 650091 China; ^2^ Electron Microscopy Center Yunnan University Kunming 650091 China; ^3^ National and Local Joint Engineering Center for Lithium‐ion Batteries and Materials Preparation Technology Faculty of Metallurgical and Energy Engineering Kunming University of Science and Technology Kunming 650093 China

**Keywords:** 3D reconstruction, asymmetric B/N‐coordination, bicontinuous structure, permeability simulation, rechargeable zinc‐air batteries

## Abstract

Cobalt‐based catalysts have demonstrated promising performance in both the oxygen reduction/evolution reaction (ORR/OER), positioning them as potential dual‐functional catalysts for recharging Zn‐air battery. However, the long‐standing challenge remains in achieving satisfactory dual‐functionality and stability of these cobalt metal centers. Herein, bicontinuous structured nanofibers composed of multiscale cobalt embedded in asymmetric B/N‐coordination carbon (denoted as CoBNPCF‐900) are constructed, exhibit enhanced ORR/OER activity, and enable the effective operation of zinc‐air battery. The utilization of 3D tomograph reconstruction and absolute permeability experiment simulation unravels a “pore connectivity” effect from visualizing the intricate internal porous structure and comprehending the fluid flow within internal passages. Theoretical calculations further elucidate the electronic transfer tendency and spin polarization of CoBNPCF‐900, providing a rationale for the improved performance resulting from alterations in the electronic environment surrounding active Co sites embedded in asymmetric B/N‐coordination carbon. A homemade rechargeable zinc‐air battery using CoBNPCF‐900 as the air cathode exhibits a bifunctional overpotential of 0.808 V and a battery lifetime exceeding 1706.6 h, which is superior to that of the Pt/C+RuO_2_ catalysts (526 h). This study offers new insights into constructing catalysts with 3D spatial precision and provides strong references for practical applications in energy storage and conversion electrocatalysts.

## Graphical abstract

A bicontinuous constructed cobalt active centers/asymmetric B/N‐coordination carbon catalyst is developed, which the ZAB assembled with CoBNPCF‐900 air cathode exhibits ultralong stability (1706.6 h). The permeability simulations indicate that the catalytic active site is highly exposed to the electrolyte, guaranteeing a high catalytic efficiency. Equally, DFT calculation validates that synergistic B,N‐coordination at the cobalt‐active sites enhances OER/ORR activities.

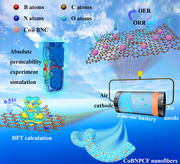

## 1 Introduction

Rechargeable zinc‐air batteries (RZABs) offer high theoretical energy density (1086 Wh kg^−1^) and the advantage of cost‐effectiveness and environmental friendliness, which have inspired significant research attention to ensure renewable electricity for the promising next‐generation energy conversion device.^[^ ^1^, ^2^ ^]^ It is also quite surprising to overcome the time‐and‐space restriction that can continuously supply their high energy density.^[^ ^3^ ^]^ Therefore, the exploitation of electrocatalysis will break this original suppressed with a high efficiency and green conversion approach.^[^ ^4^, ^5^, ^6^ ^]^ Among them, transition metal/carbon‐based hybrid electrocatalyst materials are attractive for their highly tunable electronic structure, unique coordination micro‐environment, and 3D porous construction, which endow the specific catalysts with superior conversion efficiency and high selectivity.^[^ ^7^ ^]^ These hybrid electrocatalysts also have stable catalytic activity in both oxygen reduction reaction (ORR) and oxygen evolution reaction (OER) and are adapted to various regulation strategies, thus proposing to significantly modulate bifunctional performance for RZABs.^[^ ^8^, ^9^ ^]^ In addition, pursuing a suitable interconnectional architecture and optimized surrounding environment is crucial to determining the catalytic performance and prompts them to be preferable for practical applications.^[^ ^10^, ^11^, ^12^ ^]^ However, manipulating the electronic properties and the internal porous structure of the electrocatalyst is an indispensable component that plays a vital role in further improving the energy density and conversion efficiency of RZABs.

Particularly, for the multielectron/proton transfer process in the OER/ORR at the air electrode, the attenuation issue primarily arises from the lack of connectivity within the internal pore structure and metal corrosion, resulting in the dissolution of the metal active center that generated the oxygen intermediates during the oxygen reaction process.^[^ ^13^, ^14^, ^15^ ^]^ Usually, transition metals are anchored onto carbon supports by coordination with nitrogen species.^[^ ^16^, ^17^ ^]^ However, the aggregation of metal species and the ultra‐strong electron affinity of nitrogen species will synergistically alter the local electronic structure of the catalytic center, bringing an increased reaction energy barrier that is related to poor catalytic performance.^[^ ^18^, ^19^ ^]^ Moreover, the carbon network support can be flexibly incorporated or replaced by secondary heteroatoms with relatively low electronegativity, such as sulfur (S), phosphorus (P), and boron (B), while the heteroatoms can accept electrons based on their electron deficiency, in which local distortion in the structure of sp^2^‐hybridized carbons and rupture of the charge neutrality will optimize the localized charge density and induced a shifted Fermi level, resulting in the formation of different reactive groups and enhancing catalytic activity for ORR/OER.^[^ ^20^, ^21^, ^22^, ^23^, ^24^, ^25^ ^]^ Meanwhile, it largely maximizes the metal atom utilization rate and the geometric and electronic properties of metal species, which significantly impact the mass transfer and gas release behavior.^[^ ^26^, ^27^ ^]^ Therefore, optimizing the active site and creating an effective internal flow channel in the transition metal/carbon‐based hybrid electrocatalyst is highly desirable for practical applications and accessible to establish the interplay between transition metal and carbon support species. However, it remains more challenging.

Herein, we pioneer the use of Joule Heating technology to trigger modulation of Co electronic characteristics onto asymmetric B/N‐coordination carbon nanofiber and local microenvironments, and construct an efficient bicontinuous structured CoBNPCF‐900 bifunctional electrocatalyst for Zn‐air batteries. By means of the 3D tomograph reconstruction technology, it reveals a bicontinuous structure for the CoBNPCF‐900 composed of multiscale Co nanomonomer, which not only provides abundant active sites and reaction regions for catalysis, but also enhances mass and electron transfer. Additionally, advanced absolute permeability experiment simulations were utilized to visually depict the complex internal porous structure and understand fluid flow within internal passages. The Badder charge analysis indicates that when Co is anchored in BCN, the number of valence electrons on the surface of Co decreases, while the number of valence electrons near C and N increases. This demonstrates that the Co clusters/nanoparticles (NPs) will enhance the electronic coupling at the heterojunction interface, facilitating the adsorption/activation of the oxygenic species. Meanwhile, the density of state (DOS) calculations suggests that Co serves as the primary catalytic active center in the CoBNC, and the embedded Co‐centered moieties enhance the conductivity and lower the reaction barriers. The first‐principles calculations establish Co@BNC as a high‐performance bifunctional oxygen electrocatalyst, exhibiting exceptional activity toward both OER and ORR due to synergistic B,N‐coordination at the cobalt‐active sites. When incorporated into the Zn‐air battery, the rational CoBNPCF‐900 electrocatalyst exhibits outstanding bifunctional performance with a voltage gap of 0.808 V and an exceptionally long cycle life exceeding 1706.6 h. This study presents a viable approach to the design and synthesis of a highly efficient and stable Co‐based/carbon catalyst with abundant nanoreactors, showing significant potential for practical applications in rechargeable Zn‐air batteries.

## 2 Results and Discussion

**Figure 1:**
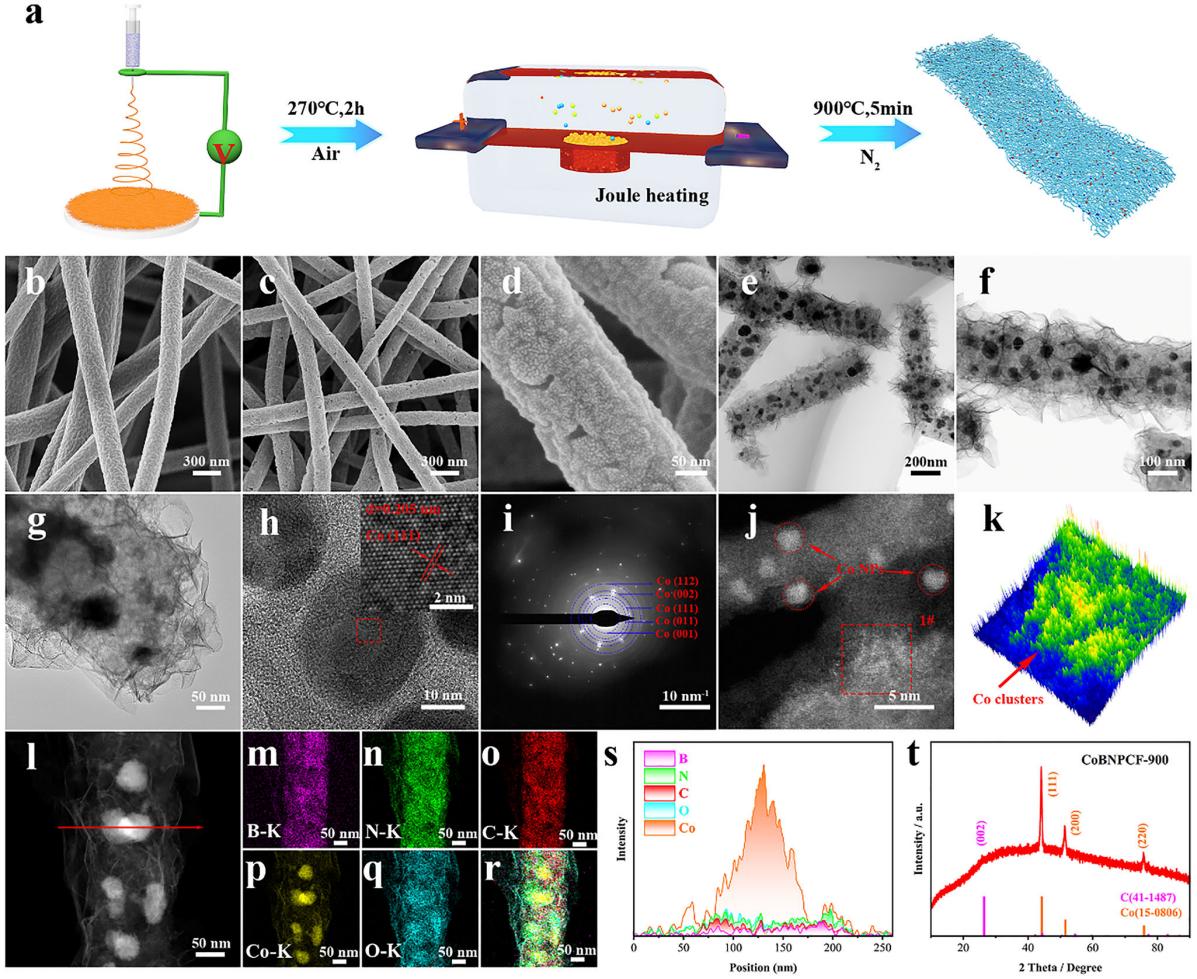
a) Schematic illustration for the synthesis processes of CoBNPCF‐900. b) SEM image of precursor membrane. c,d) SEM image, e,f) Bright Field Scanning (BF‐S) images, g) TEM, h) HRTEM, i) SAED images of CoBNPCF‐900. j) AC‐TEM of CoBNPCF‐900. k) corresponding 3D intensity profile fitting images from area 1^#^ in (j). (l–r) HAADF image and corresponding EDS mapping, and t) XRD pattern of CoBNPCF‐900.

CoBNPCF‐900 is successfully synthesized by controlling electrostatic spinning and subsequent joule heating process. The fabrication process is schematic depicted in **Figure** **1**a, and its details are described in Experimental Sections “Synthesis of the Cobalt Acetate [Co(AC)2] / Hydroxy Benzene Boronic Acid [HBBA]/ Polyvinylpyrrolidone [PVP] Precursor Membranes” and “Synthesis of the Porous CoBNPCFs‐T (T = 600, 700, 800, 900, 1000 °C) Nanofibers.” The difference in the synthesis method of CoBNPCFs‐T (T = temperature, 600–1000 °C) catalysts lies in the change in temperature during the joule heating process. First, the detailed morphology and crystallographic form are investigated in Figure 1. The scanning electron microscopy (SEM) and transmission electron microscopy (TEM) images of the as‐prepared CoBNPCFs precursor exhibits a 3D randomly woven network linked by numerous nanofibers and uniform aggregated small wrinkles on the surface (Figure 1b; Figure S1a, Supporting Information). After undergoing pyrolysis reaction at 900 °C through ultrafast flash joule heating, CoBNPCF‐900 inherited this skeleton construction and appeared into a shrank fiber composed of many open pores and nanoparticles (Figure 1c). Moreover, many Co NPs are encapsulated in nanofibers, and abundant clusters are exposed to the surface thanks to Co‐metal precipitation (Figure 1d; Figure S3, Supporting Information). Notably, various ultra‐thin nanosheets are self‐assembled outside of the nanofibers and provide enough available space in this 3D woven network (Figure 1e–g; Figure S4–S6, Supporting Information). As shown in Figures S1 and S2 (Supporting Information), the diameter of fibers decreased with increasing pyrolysis temperatures, in which the presence of pores and nanoparticle space distribution also proved similar to the CoBNPCF‐900 structure under different temperatures (Figure S4, Supporting Information). But for CoBNPCF‐1000, the fractured fibers are obtained that may result from the exorbitant pyrolysis temperature. These fractured fibers cause the uneven exposure of active sites, reduce the efficiency of electron transmission, and affect the diffusion and transmission of oxygen within the catalytic layer, thereby leading to the slower reaction kinetics.^[^ ^28^, ^29^ ^]^ Moreover, the interplanar spacings of 0.205 nm assigned to the Co (111) crystal plane and corresponding diffraction rings corresponded to (001), (011), (111), (002), and (112) crystal planes of Co metal (Figure 1h,i). The co‐existence of Co clusters and NPs dispersion state is further confirmed by spherical aberration‐corrected scanning transmission electron microscope (AC‐STEM) (Figure 1j; Figure S7, Supporting Information), and the corresponding 3D intensity profile fitting images from area 1^#^ also proves the distribution of Co clusters on the surface of nanofibers (Figure 1k). At the same time, the carbon fiber support collectively forms a highly conductive network with an exceptionally high specific surface area, which immobilizes cobalt nanoparticles and disperses cobalt cluster active sites throughout the matrix. This multiscale interactive architecture effectively reduces particle agglomeration and enhances charge transfer during electrostatic reactions.^[^ ^26^, ^27^ ^]^

To further evaluate the distribution of elements across CoBNPCF‐900, EDS mapping image and line scanning analysis were performed, and identified homogeneous scattering of B, N, and Co elements along with the carbon fiber, and the Co signal is also present on the entire carbon fiber, indicating the highly‐scattered Co species (Figure 1l–r). In addition, the unique Co@BNC core‐shell structure that Co NPs embedded in the asymmetric B/N‐coordination carbon fibers is characterized by the EDS overlap image, and the O signal may be from the slight surface oxidation. The direction of line scanning (Figure 1s) was indicated by the red arrow in Figure 1l, and two crucial messages can be revealed: 1) The signal intensity of B, N, and C elements ranging from ≈75 to 175 nm and no significant degradation proves that Co nanoparticles were uniformly wrapped by B, N, and C species. 2) Two signal intensity of Co, B, N, and C elements located at 0–50 nm and 225–250 nm both demonstrate the Co clusters appear with the B, N species and their probable coordinated effect. The powder X‐ray diffraction (XRD) patterns of CoBNPCF‐900 (Figure 1t) depict the distinct crystal peaks of cobalt and carbon species. The weak crystal peaks in Figure S8 (Supporting Information) of CoBNPCF‐600 indicate the inferior crystallinity of cobalt species and insufficient carbonization of carbon fibers under low pyrolysis temperatures. In contrast, the prominent crystalline peaks emerging above 600 °C demonstrate that elevated temperatures facilitate catalyst crystallization and carbon fiber graphitization, corresponding to the TEM image of Figure S4 (Supporting Information). In general, the above analysis confirmed that the synthesized CoBNPCF‐900 exhibits an interconnected porous structure with a uniform boron‐nitrogen element distribution and multistate dispersion of the coexistence of the Co NPs and clusters.

The specific surface area and pore‐size distribution of the synthesis CoBNPCFs‐T catalysts is determined using the Brunauer–Emmett–Teller (BET) method that exhibited the largest BET surface area (64.34 m^2^ g^−1^) and 2–5 nm narrow range of pore‐size distribution for CoBNPCF‐900, revealing the existence of a mesoporous structure, which improves the efficiency of mass transfer between reactants and active sites, the diffusion rate of oxygen and reactants, and thus promoting the catalytic activity (Figures S9 and S10, Supporting Information).^[^ ^30^, ^31^ ^]^ On the contrary, other samples have a poor hysteresis loop corresponding to their relatively low porosities and plentiful small (2–5 nm) and large (5–40 nm) mesopores. CoBNPCF‐900 displayed type‐IV isotherms simultaneously, which are further expected to offer high surface area values and can be useful for enhancing electrolyte transport and anchoring the active sites. Two strong characteristic peaks are observed in the Raman spectrum of CoBNPCFs‐T catalysts (Figure S11, Supporting Information), corresponding to the D band (≈1340) and G band (≈1580) of the carbon support, respectively. Among them, CoBNPCF‐900 shows the higher I_D_/I_G_ ratio of 2.42, suggesting the abundant carbon defects (Figure S11f, Supporting Information). The above characterizations also demonstrate that CoBNPCF‐900 has multiple pores and abundant defects, further validating its pore connectivity effects and excellent electrical conductivity. In addition, as OER/ORR occurs at the electrode/electrolyte interface, the accessibility of active centers is determined by the porous structure and surface wettability.^[^ ^32^ ^]^ To probe the surface wettability of the synthesized catalysts, the solid‐liquid contact angles (CA) and the underwater bubble contact angles were determined through measurement. Figure S12 (Supporting Information) shows that CoBNPCF‐900 possesses a stronger hydrophilicity (11°) and maintains a superaerophobic surface (177.1°). These indicate that the electrode surface can rapidly absorb the electrolyte, and effectively prevent bubble accumulation on the catalyst to ensure sufficient transport channels for bubble removal, thereby leading to remarkable enhancement in catalytic activity of CoBNPCF‐900.^[^ ^33^, ^34^ ^]^

**Figure 2:**
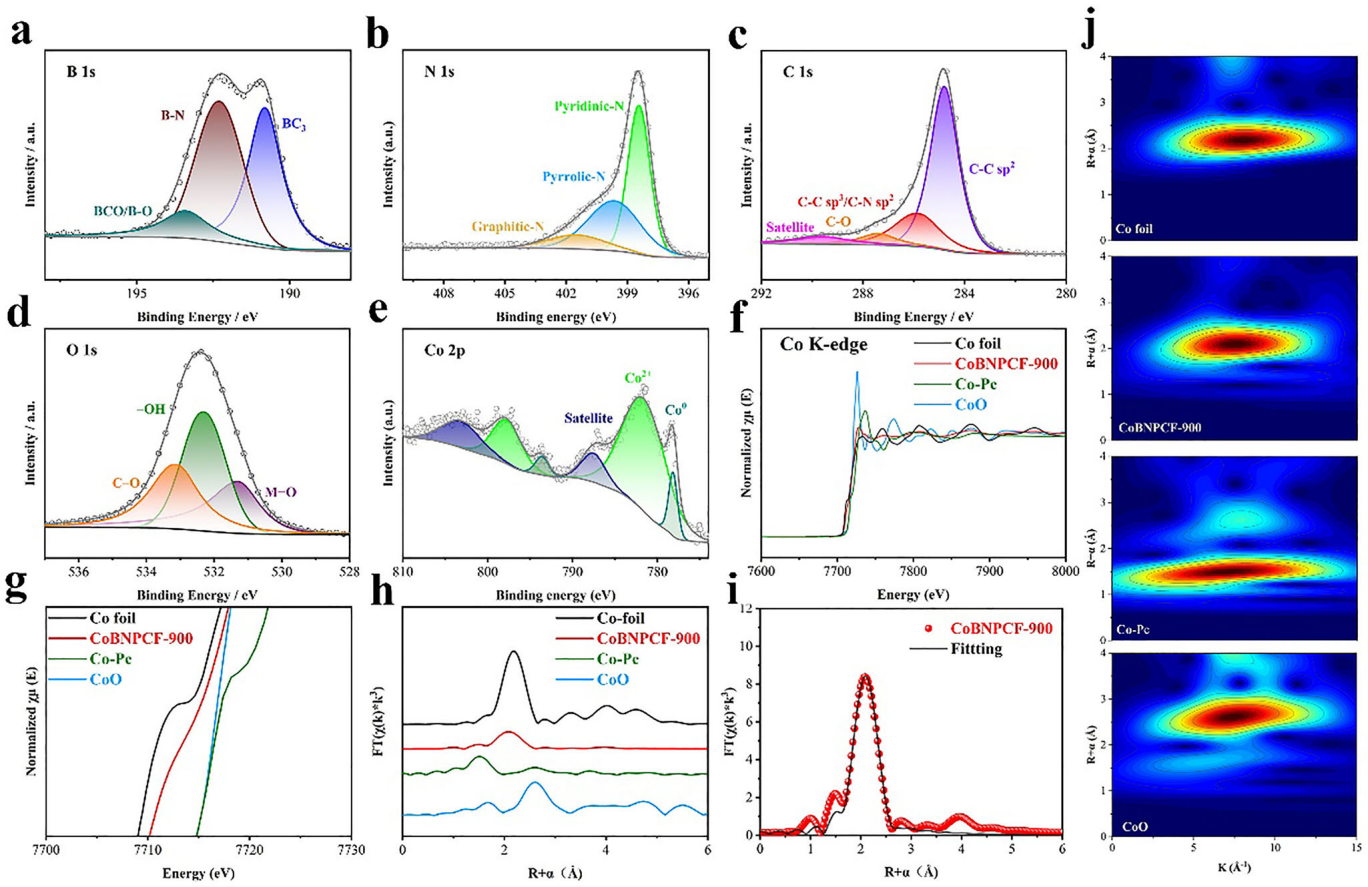
High‐resolution XPS spectrum of CoBNPCF‐900, a) B 1s, b) N 1s, c) C 1s, d) O 1s, e) Co 2p. f) Co‐K‐edge X‐ray absorption near‐edge structure (XANES) spectrum and references. g) The magnified pre‐edge XANES region. h) Fourier transformation of the extended X‐ray absorption fine structure spectrum and references. i) First‐shell fitting of Fourier transformations of EXAFS spectrum. j) Wavelet transform of and references for CoBNPCF‐900.

Moreover, the valence states and surface chemical composition of CoBNPCFs‐T catalysts were further investigated using X‐ray photoelectron spectroscopy (XPS) measurements. The survey spectrum in Figure S13 (Supporting Information) of CoBNPCFs‐T verifies the existence of B, N, C, O, and Co elements without any impurity, consistent with the above EDS results. The high‐resolution B 1s spectrum displays three characteristic peaks, including BC_3_ (≈190.8), B‐N (≈192.3), and BCO/B‐O (≈193.4) (**Figure** **2**a; Figure S14, Supporting Information).^[^ ^35^ ^]^ CoBNPCF‐900 has the highest total content of B element, and the calculative content of BC_3_ of 2.03 atom%, confirms the successful incorporation of B heteroatom with relatively low electronegativity (Table S1, Supporting Information). The high‐resolution N 1s spectrum is fitted into three peaks located at 398.4, 399.6, and 401.5 eV, attributed to pyridinic‐N, pyrrolic‐N, and graphitic‐N, respectively, indicates the existence of asymmetric B/N‐coordination carbon in CoBNPCF‐900 (Figure 2b; Figure S15, Supporting Information).^[^ ^36^, ^37^ ^]^ These heteroatom dopants can optimize the local coordination environment around active metal centers, thereby enhancing the catalytic activity for ORR/OER.^[^ ^38^ ^]^ The detailed comparison of the contents of various N species in Table S2 (Supporting Information), shows the highest concentration in CoBNPCF‐900 that ensures its preferable electrocatalytic activities. The High‐resolution of C 1s spectrum can be deconvoluted into C─C sp^2^ (≈284.8 eV), C─C sp^3^ /C─N sp^2^ (≈285.8 eV), C─O (≈287.4 eV), and Satellite (288.8 eV), respectively (Figure 2c; Figure S16, Supporting Information).^[^ ^39^ ^]^ In addition, three peaks at 531.5, 532.5, and 533.5 eV, can be assigned to M─O, ─OH, and C─O bonds in the O 1s spectrum, proving that the strong adsorption capacity for the oxygenated species and the slight oxidation occur on the metal surface during synthesis, which leads to the metal surface reconstruction, and thus promoting the OER/ORR performance (Figure 2d; Figure S17, Supporting Information).^[^ ^40^, ^41^, ^42^ ^]^ The high‐resolution Co 2p spectrum can be deconvoluted into metallic Co (at ≈778.1 and ≈793.6 eV), Co^2+^ (at ≈781.6 and ≈797.8 eV), and Satellite (at ≈787.5 and ≈803.2 eV) (Figure 2e; Figure S18, Supporting Information).^[^ ^37^, ^43^ ^]^ In addition, to verify the existence of Co─B/N─C structures, the electron energy loss spectroscopy (EELS) was further performed. For comparison, the EELS spectra collected around Co particles (red line) and those from Co‐free regions (blue line) (Figure S19a, Supporting Information). The EDS mapping exhibits the existence of Co particles and the uniform distribution of B, N, and C elements in Figure S19b–e (Supporting Information). As shown in Figure S19f–i (Supporting Information), the K‐edges of B, N, and C are detected in the vicinity of the metal particles, demonstrating a mixed B/N‐C coordination environment for the Co particles.

To investigated the coordination environments and local electronic structures of the CoBNPCF‐900, X‐ray absorption spectroscopy (XAS) measurements is carried out. As indicated in Co K‐edge X‐ray absorption near‐edge structure (XANES) spectrum (Figure 2f,g), the adsorption edge of CoBNPCF‐900 is higher than that of Co foil but lower than those of Cobalt Phthalocyanine (Co─Pc) and CoO, revealing partial oxidation of Co species, and its adsorption edge is located between Co foil and CoO uncover an electron‐deficient characteristics of the Co species.^[^ ^44^ ^]^ As shown in Figure 2h, Fourier‐transformed k^3^‐weighted EXAFS (FT‐EXAFS) spectrum of CoBNPCF‐900 with a weak peak at 2.08 Å that corresponds to the Co─Co bond, which is ≈0.1 Å smaller than the peak position of the Co─Co bond in Co foil (2.18 Å), indicating an obvious discrepancy of the bond length between Co and coordination atoms after B, N atom doping in CoBNPCF‐900.^[^ ^45^ ^]^ In CoPc, the primary Fourier transform (FT) peak at ≈1.5 Å corresponds to Co─N bonds. For CoBNPCF‐900, a secondary FT peak detected at ≈1.48 Å is assigned to Co─B/N coordination bonds. Boron incorporation induces a slightly shift in this peak, indicative of altered coordination environment around the Co centers.^[^ ^46^, ^47^ ^]^ Particularly, the fitting curve of CoBNPCF‐900 using Co species is perfectly matches the EXAFS data (Figure 2i; Figure S20, Supporting Information), and these results were further verified by analyzing the discrepancy in wavelet transforms among the obtained catalysts.^[^ ^48^ ^]^ The distinct peaks in Figure 2j demonstrate the Co foil (2.2 Å, 7.9 Å^−1^), CoBNPCF‐900 (2.09 Å, 7.48 Å^−1^), Co‐Pc (1.51 Å, 7.02 Å^−1^), CoO (2.61 Å, 7.41 Å^−1^), and shown the slight distance differences for the k‐space frequency peak, further verifying the partial bonding of Co‐B/N in CoBNPCF‐900. In addition, this lower k‐space frequency and the asymmetrical peak in CoBNPCF‐900 can be explained by the different distances between the Co atom and B/N atoms. Specifically, N sites within the coordination environment are partial substituted by B atoms, leading to the formation of asymmetrical Co‐B/N bond in CoBNPCF‐900.^[^ ^36^, ^37^ ^]^ Based on the above analysis, the B, N, C, O, and Co element, and further asymmetric coordination of B/N with the Co atom are confirmed and consistent with XPS and EDS results.

**Figure 3:**
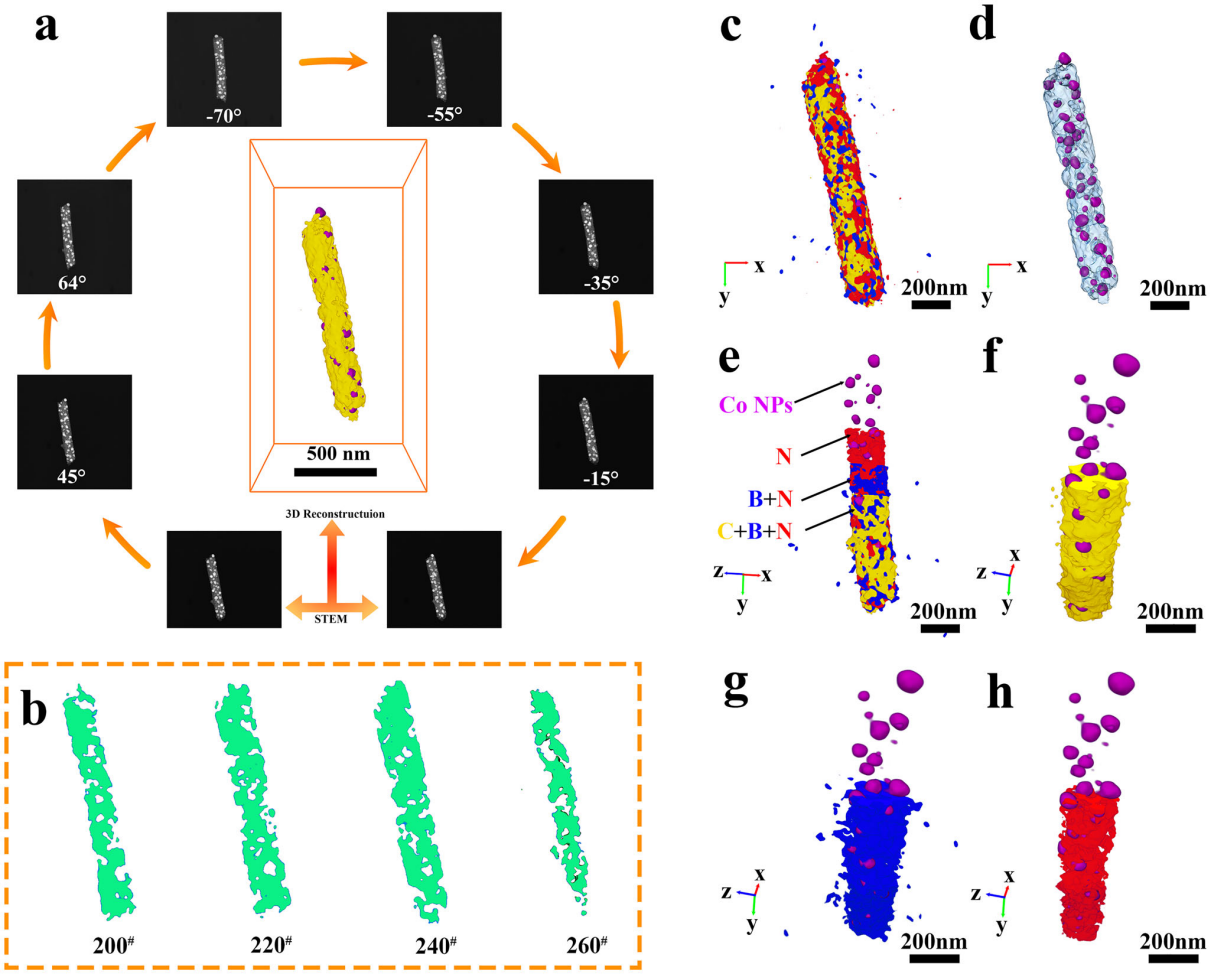
a) STEM‐tomography images at different rotation angle and the corresponding reconstructed model of CoBNPCF‐900. b) Ortho slices at No. 200, 220, 240, 260. c) Co NPs and B, N elements, d) Distributions of Co NPs in carbon fiber. e) The core‐shell structure of Co@BNC. f) Co@C, g) Co@B, h) Co@N elements distribution model.

To gain insight into the internal microenvironment of CoBNPCF‐900, 3D tomograph reconstruction was conducted. Movie S1 and Figures S21–S25 (Supporting Information) display the ortho slices of CoBNPCF‐900 acquired from −70° to 64° by STEM tomography, and then to arrange the based on the intensity threshold to order obtain the reconstructed 3D model (**Figure** **3**a; Figure S26, Supporting Information). The CoBNPCF‐900 model exhibits a typically fiber‐like construction with numerous encapsulated nanoparticles and displays a high degree of similarity to the obtained HAADF images from −15° to 45°, indicating the availability and authenticity of this reconstruction model. The typical ortho slices at No. 200, 220, 240, and 260 (Figure 3b) further illustrate the internal porous structure, corresponding the slice position shown in Figure S27 (Supporting Information). Moreover, as shown in Movie S2 (Supporting Information), the 3D distributions of the elements (including C, B, N, and Co) and the bicontinuous porous structure of CoBNPCF‐900 are disclosed.^[^ ^49^ ^]^ Numerous defects in the carbon nanofiber and the encapsulated Co NPs are directly observed in Figure 3c,d. Meanwhile, the uniformly distribution of N and B elements is shown in Figure 3e and Figure S28 (Supporting Information), matching accurately with the EDS mapping results. Equally, Figure 3f–h and Figure S29 (Supporting Information) shows that the formatted core‐shell structure, consist of Co species and asymmetric B/N‐coordination, has an abundant carbon defects, which enables us to draw the following conclusions: 1) The bicontinuous pores and plentiful defects lead to a high specific surface area of carbon nanofiber; 2) Co NPs with widely‐distributed sizes are randomly multistate dispersion and densely encapsulated by C, B, and N elements; 3) The asymmetric B/N‐coordination carbon nanofiber can optimize the local coordination environment of the Co active metal centers.

**Figure 4:**
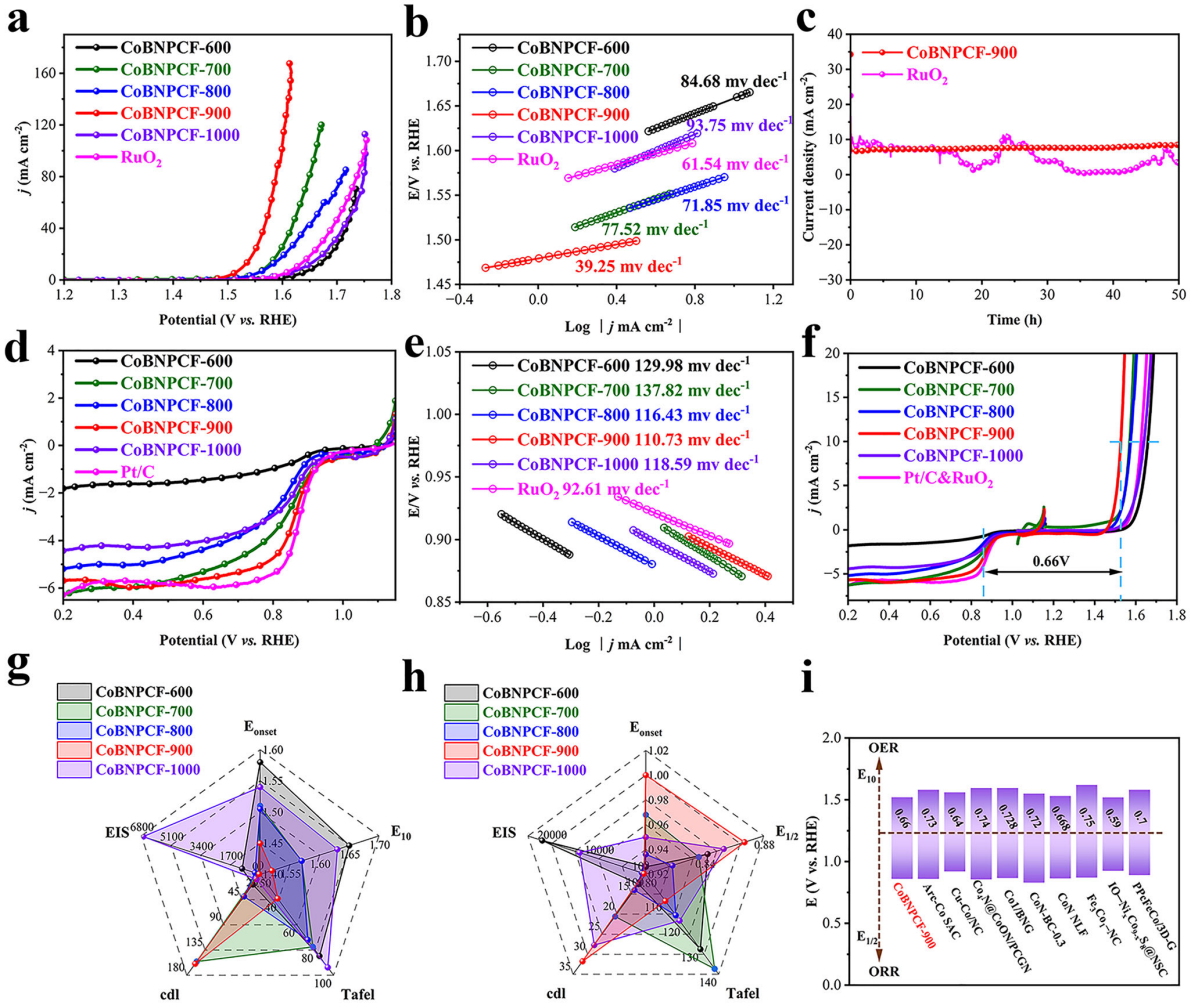
a) The LSV curves with iR correction, b) Tafel plots, and c) the chronoamperometric responses (I‐T) of CoBNPCFs‐T (T = 600, 700, 800, 900, 1000) toward OER. d) ORR LSV polarization curves with iR correction, and e) Tafel plots. f) Composite LSV curves for determination of ΔE. g) OER and h) ORR performance metrics statistics. i) Comparison potential gap ΔE values of our catalyst with the researches in recent years.

Furthermore, the electrocatalytic capability of CoBNPCFs‐T catalysts is further evaluated at alkaline environment. The OER activity and their linear sweep voltammetry (LSV) curves are performed in 1 M KOH by the rotating disk electrode method (**Figure** **4**a; Figures S30 and S31a, Supporting Information). CoBNPCF‐900 has a gratifying onset potential (E_onset_) and E_10_ (10 mA cm^−2^) value of 1.45 and 1.52 V, respectively, which is also significantly small than those of CoBNPCF‐600 (1.58 V, 1.65 V), CoBNPCF‐700 (1.51 V, 1.57 V), CoBNPCF‐800 (1.505 V, 1.57 V), and CoBNPCF‐1000 (1.54 V, 1.63 V), and are 80 and 100 mV more negative in comparison to that of the commercial RuO_2_ (1.53 V, 1.62 V), confirming its prominent OER catalytic activity. The charge transport kinetics is also evaluated using Tafel slope in Figure 4b, CoBNPCF‐900 possesses a smaller slope value of 39.25 mV dec^−1^ in contrast to that of CoBNPCF‐600 (84.68 mV dec^−1^), CoBNPCF‐700 (77.52 mV dec^−1^), CoBNPCF‐800 (71.85 mV dec^−1^), CoBNPCF‐1000 (93.75 mV dec^−1^), and RuO_2_ (61.54 mV dec^−1^). Additionally, the electrochemical impedance spectroscopy (EIS, recorded at +1.573 V, ranging from 100 kHz to 0.01 Hz) shows a minimum impedance value of 60 Ω for CoBNPCF‐900 (Figure S31b, Supporting Information), indicating its fast charge transport and accelerated OER kinetics.

Figure S32 (Supporting Information) displays the double‐layer capacitance (C_dl_) value and the corresponding continuous cyclic voltammetry (CV) at different scan rates. The C_dl_ values of CoBNPCFs‐T catalysts decreased in the order of CoBNPCF‐900 (159.42 mF cm^−2^) > CoBNPCF‐700 (155.55 mF cm^−2^) > CoBNPCF‐800 (39.55 mF cm^−2^) > CoBNPCF‐600 (18.14 mF cm^−2^) > CoBNPCF‐1000 (8.82 mF cm^−2^), demonstrating largest electrochemically active area (ECSA) and more exposed active sites in CoBNPCF‐900. Besides, the prolonged stability is also a factor that should be taken into account in OER. Figure 4c shows the OER polarization undergoing at 1.573 V and no obvious significant reduction after 50 h, during which no obvious significant current reduction is observed, indicating its long stability for CoBNPCF‐900. On the contrary, the commercial RuO_2_ displays a noticeable fluctuation relatedly to its poor durability. Therefore, the accelerated catalytic kinetics and largest C_dl_ values ensured CoBNPCF‐900 have a most expedited electron transport and best OER performance among a series of CoBNPCFs‐T catalysts (Figure 4g).

On the other hand, the ORR activities of CoBNPCFs‐T catalysts and the commercial Pt/C (20% wt.) are explored in 0.1 M KOH. In Figure 4d and Figures S33 and S34a (Supporting Information), CoBNPCF‐900 has an excellent ORR catalytic performance with a half‐wave potential (E*_1/2_*) of 0.867 V, which is superior than that of CoBNPCF‐600 (0.842 V), CoBNPCF‐700 (0.836 V), CoBNPCF‐800 (0.818 V), and CoBNPCF‐1000 (0.853 V), and required a small gap of 11 mV compared to Pt/C (0.878 V), demonstrating its excellent ORR performance. The CoBNPCF‐900 also display a favorable ORR kinetics with a Tafel slope of 110.73 mV dec^−1^, which is the smallest among CoBNPCFs‐T samples and close to Pt/C (92.61 mV dec^−1^), suggesting its favorable thermodynamically kinetic (Figure 4e). Meanwhile, the obtained EIS value of CoBNPCF‐900 is 373.9 Ω, which is lower than those of CoBNPCF‐600 (21 kΩ), CoBNPCF‐700 (427.6 Ω), CoBNPCF‐800 (837.9 Ω), and CoBNPCF‐1000 (14 kΩ), verifying its accelerated electron transfer efficiency at the solid‐liquid interface (Figure S34b, Supporting Information). Furthermore, the C_dl_ value of CoBNPCF‐900 (31.76 mF cm^−2^) is largest in comparison with the other CoBNPCFs‐T catalysts reflects its largest ECSA in ORR process (Figure S35, Supporting Information). As shown in Figure S36 (Supporting Information), just a current reduction of 11.4% is observed in CoBNPCF‐900 after a long‐term stability test of 50 h, reveals exceptional durability in practicality application. The above analysis shows that CoBNPCF‐900 possesses the best catalytic performance among CoBNPCFs‐T catalysts and thus effectively enhance ORR process (Figure 4h). Meanwhile, the bifunctional activity parameter ΔE (ΔE = E_1/2_–E_10_) of CoBNPCF‐900 is then evaluated (Figure 4f), a voltage gap of 0.66 V is completely related to its catalytic reversibility of the oxygen redox reactions, and smaller than most recently reported bifunctional catalysts (Figure 4i and Table S3, Supporting Information), reflecting its outstanding OER/ORR performances.

**Figure 5:**
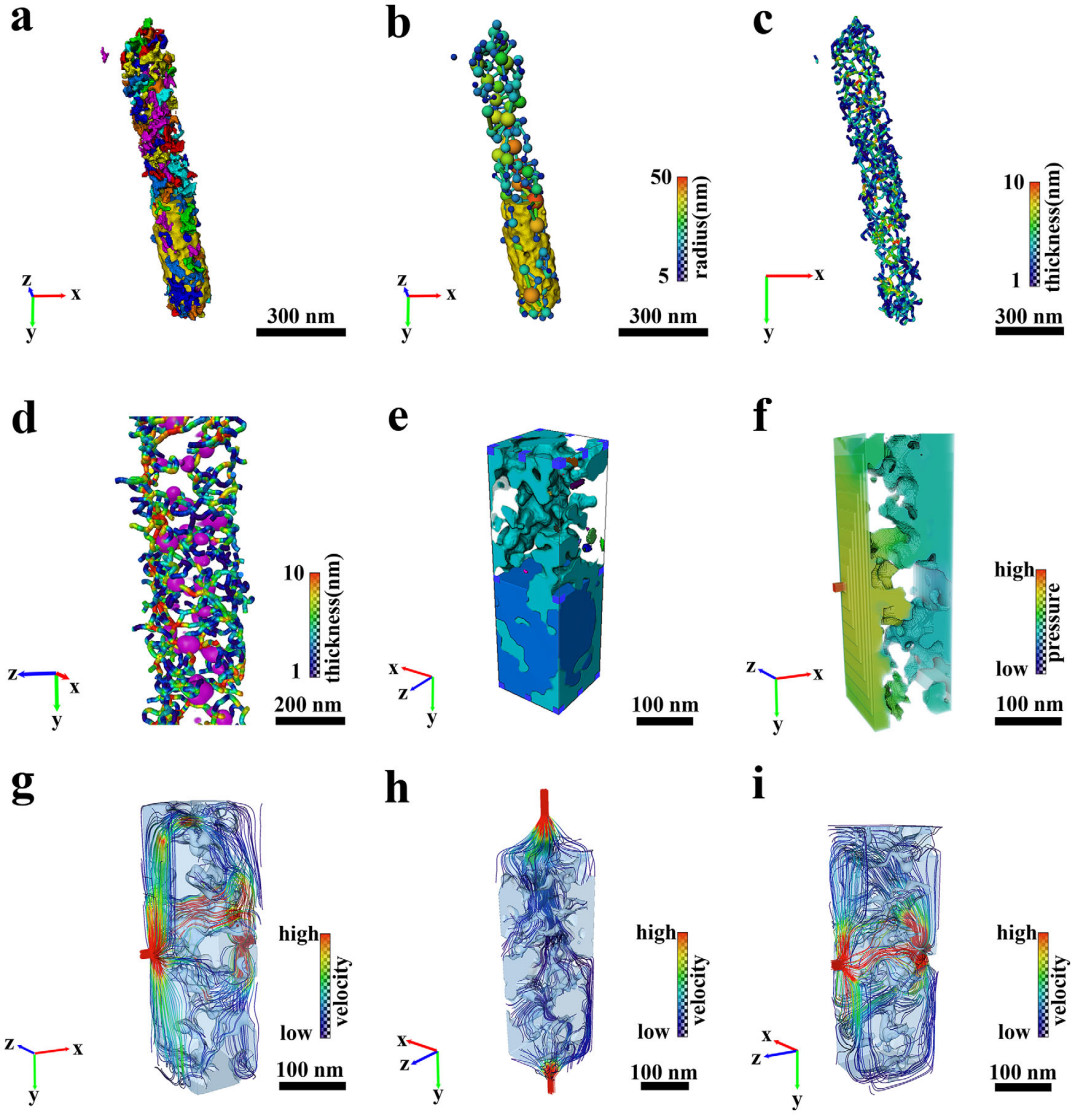
a) 3D reconstruction performed internal pores distribution. b) Pores and throat distribution in carbon fiber. c) The skeleton of channels. d) Location of Co nanoparticles and channels. e) Pores and carbon matrix from extraction sub‐volume. f) Fluid pressure distribution. Absolute permeability experiment simulated streamline in g) x direction, h) y direction, and i) z direction.

To further gain insight into the internal microenvironment of CoBNPCF‐900, 3D tomograph reconstruction and absolute permeability simulation experiment were carried out. Movie S3 (Supporting Information) presents the distinctive bicontinuous porous construction in the simulative CoBNPCF‐900 model. **Figures** **5**a and S37 (Supporting Information) depict all pores of isolated and connected ones in a unit view and reflect the variate location of the sub‐pore network using diverse colors, in which the reconstructed authenticity is guaranteed by the precise segmentation of pores and the carbon matrix (Figure S37f–h, Supporting Information). The connected pores are particularly emphasized in Figure 5b and Figure S38 (Supporting Information) that constitute the interconnected channels inside the CoBNPCF‐900 to promote the electrolyte transfer and bubble escape. The bubble nucleation is tend to occur at the top part of the catalyst, thereby first translate maintaining the effectively catalytic activity and stability of CoBNPCF‐900, thus the internal pores and throats are further depicted by a ball‐stick model to intuition reflects this interconnected porous structure, in which the balls and sticks respectively represented the practical pores and throats, and the analysis for the size of the pores and throats, and the coordination number is shown in Figure S39 (Supporting Information). The simulative pores have an average size of 23.423 nm with a radius ranging from 15 to 30 nm, and the interconnected pores characterized by an average coordination number of 3.23, indicating the existing adjacent crosslinked network and its high distribution frequency. As a great passage for fluid transport, the size of the throat was also evaluated, which have an average radius of 9.56 nm with the radius ranging from 1 to 26 nm, and a mean throat length of 72.15 nm, indicating its open channels coupled with abundant mesopores that are crucial for the effective transportation and appropriate surface functionalities. Thus, the hierarchical pores and interconnected throats together form an interwoven microenvironment and providing an effective bicontinuous cavity for OER/ORR.

Additionally, the movement trajectory of the intricate channels is also simulated in Figure 5c, which also offering outstanding pathways for liquid transport and gas release. As shown in Figure 5d and Figure S40 (Supporting Information), some Co NPs are surround by the channels and most of Co NPs are located at the channel junction that features a more dispersing with a high loading, this distinctive channel architecture possess a enhancing advantageous for mass transport. Compared with the above characterization, this outstanding Co@BNC and bicontinuous internal channels highly enables the active site to the electrolyte and then guaranteeing a high catalytic efficiency.

To further validate the fluid transport process in this bicontinuous cavity, the absolute permeability simulation experiment is carried out. First, an internal sub‐volume cube (volume: 60 × 200 × 60 nm) was extracted from the reconstructed model (Figure S41a, Supporting Information), and the range of interactive thresholding have been adjusted to segmented the pores and the carbon matrix (Figure S42, Supporting Information). Figure 5e and Figure S41b–f (Supporting Information) present the segmentation of sub‐volume cube and the corresponding isolated and connected pores. Movie S4 (Supporting Information) and Figure 5g–i discloses the simulated streaming process of the electrolyte in the x, y, and z directions, which distinctly illustrate the flow path and velocity of the electrolyte access the cavity of CoBNPCF‐900. Meanwhile, Figure 5f and Figure S43 (Supporting Information) shows the pressure distribution of the electrolyte that exhibits a lower pressure in the z direction compared to that of the x and y directions. Furthermore, the limiting factor of the mass transfer process is conducted using the volume fraction that assigned to the volume ratio of the pore to carbon matrix in each ortho slice, which is able to reveals the presence state of the pores in various directions. The volume fraction curve of the yz plane displays a rapid variation tendency between the one slice to the adjacent plane in the x direction, while a minor change tendency is appeared for the xy plane in the z direction (Figure S44, Supporting Information). The significant differences for the volume fraction, reflecting the substantial alterations of the electrolyte pressure, and thus further affect the electrolyte flow. Specifically, CoBNPCF‐900 have more permeable to the electrolyte in the radial direction compared to the axial direction, implying its high transfer kinetic and outstanding catalytic durability. In general, the formation of this bicontinuous nanostructure is directly evidenced by the unique intertwined microenvironment, the advanced electrolyte flow simulation, and the distinct volume fraction, which enhances the electrolyte transport during catalysis, thereby creating an extensive active interface for OER/ORR. As a superior ion/electron transport channel, this intertwined nanostructure provides a localized microenvironment with regionalization confinement and accumulation effects, thereby improving mass transport and optimizing the transformation/adsorption for the reactive intermediates.^[^ ^50^ ^]^ Therefore, CoBNPCF‐900 characterized by the superior OER/ORR catalytic activity and durable stability attributed to its distinctive bicontinuous structure, which is expected to avoid the accumulation in the catalytic process and enhance the release competence for the bubble.

**Figure 6:**
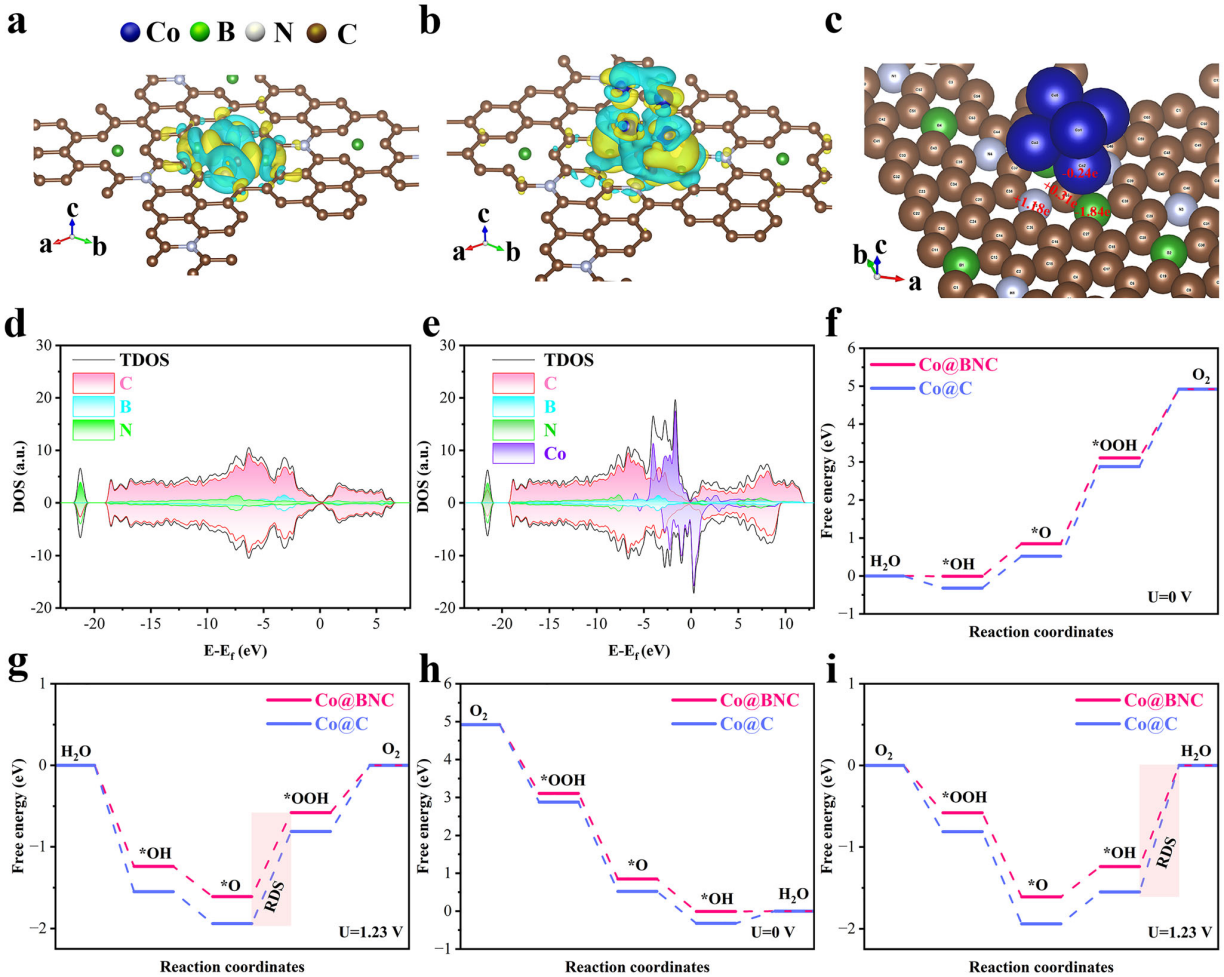
a,b) Differential charge density of BNC and Co@BNC. c) Bader charge analysis of Co@BNC. d) DOS of BNC. e) DOS of CoBNC. f,g) Gibbs Free energy diagrams of the OER processes on BNC and Co@BNC at U = 0 V and U = 1.23 V. h,i) Gibbs Free energy diagrams of the ORR processes on BNC and Co@BNC at U = 0 V and U = 1.23 V.

To understand insight into the enhanced OER/ORR activity of such a configuration for CoBNPCF‐900, the catalytic process on Co‐centered moieties embedding in BNC is conducted by the density functional theory (DFT) calculation. Two model structures, including unsymmetrical Co@BNC (Co metal center with the asymmetric B/N‐coordination carbon) and BNC are built with structural characteristics demonstrated from XAS results, and the charge density distributions are carried out to analyze the electronic properties difference between BNC (**Figure** **6**a) and Co@BNC (Figure 6b). For the BNC model, an obvious charge‐transfer from B, N atom to C atom, but an electronic rearrange and the flow of electrons from Co, B atom to N, C atom after embedding the Co‐centered moieties into the BNC model due to the low electron affinity of Co and B atom. Additionally, Figure S45 (Supporting Information) shows the Bader charge value of B, N, and C is −1.93e, +1.17e and +0.28e, respectively, indicating that B is prone to lose the electron density, while N and C trend to accumulate electron density. After introducing the Co‐centered moieties into BNC, the changed Bader charge value displays an electronic rearrange based on the charge decrease surrounding the Co and B atoms and the corresponding charge increase in the N and C atoms (Figure 6c). Compared to the BNC model, the charge transfer of Co@BNC increases dramatically (Figure S46, Supporting Information), demonstrating that the Co clusters/NPs will strengthen the electronic coupling at the heterojunction interface that facilitating the adsorption/activation of the oxygenic species.^[^ ^51^ ^]^ The calculated density of states (DOS) of BNC and Co@BNC is shown in Figure 6d,e, further revealing an asymmetric electronic structure between BNC and Co@BNC models, in which a significant spin polarization is obtained in the Co@BNC model. Particularly, the spin‐down electrons in the Co atom make a smaller TDOS that is close to the Fermi level, while the TDOS of the other electrons in the spin‐up part is weak, which will lead to a lower activation barrier for the transfer of electrons to the surface adsorbates.^[^ ^52^ ^]^ This suggests that Co serves as the primary catalytic active center in the Co@BNC, and the embedded Co‐centered moieties enhance the conductivity and lower the reaction barriers.^[^ ^53^ ^]^

To clarify the active sites of Co@BNC and the origin of its enhanced activity upon B‐N co‐doping, we conducted first‐principles calculations to explore the stepwise OER and ORR pathways. Figure 6f,g presents the calculated OER free energy diagrams for Co@BNC and Co@C, while optimized geometries with adsorbed oxygen intermediates are detailed in Figures S47 and S48 (Supporting Information). At U = 0 V, the rate‐determining step (RDS) for the two models is *O species bonds with OH^−^ to form *OOH, with Co@BNC and Co@C exhibiting the energy barrier of 2.26 and 2.36 eV, respectively (Figure 6f and Table S4, Supporting Information). This demonstrates that the synergistic effect between B‐N and the cobalt center effectively reduces the OER energy barrier. At U = 1.23 V, the RDS remains *OOH formation, with energy barriers of 1.03 eV for Co@BNC and 1.13 eV for Co@C (Figure 6g and Table S5, Supporting Information). These results confirm that Co@BNC active sites significantly reduce the OER energy barrier, thereby enhancing intrinsic activity.^[^ ^54^, ^55^, ^56^ ^]^ Moreover, DFT calculations further probed the mechanistic origins of the ORR performance on Co@BNC and Co@C active sites (Figure 6h,i; Figures S49 and S50, Supporting Information). Co@BNC exhibits an ultralow theoretical limiting potential of 0.01 V for the ORR, outperforming Co@C (0.32 V; Figure 6h and Table S6, Supporting Information), suggesting the Co@BNC has a better electrocatalytic activity for ORR. At the equilibrium potential (U = 1.23 V), the protonation of *OH to H_2_O serves as the ORR rate‐determining step (RDS) on both Co@BNC and Co@C catalysts, with Co@BNC exhibiting a lower free energy barrier (1.24 eV vs 1.55 eV for Co@C; Figure 6i and Table S7, Supporting Information) due to synergistic B,N‐coordination enhancing ORR activity. Taken together, these results establish Co@BNC as a high‐performance bifunctional oxygen electrocatalyst, exhibiting exceptional activity toward both OER and ORR due to synergistic B,N‐coordination at the cobalt‐active sites.^[^ ^57^, ^58^ ^]^

**Figure 7:**
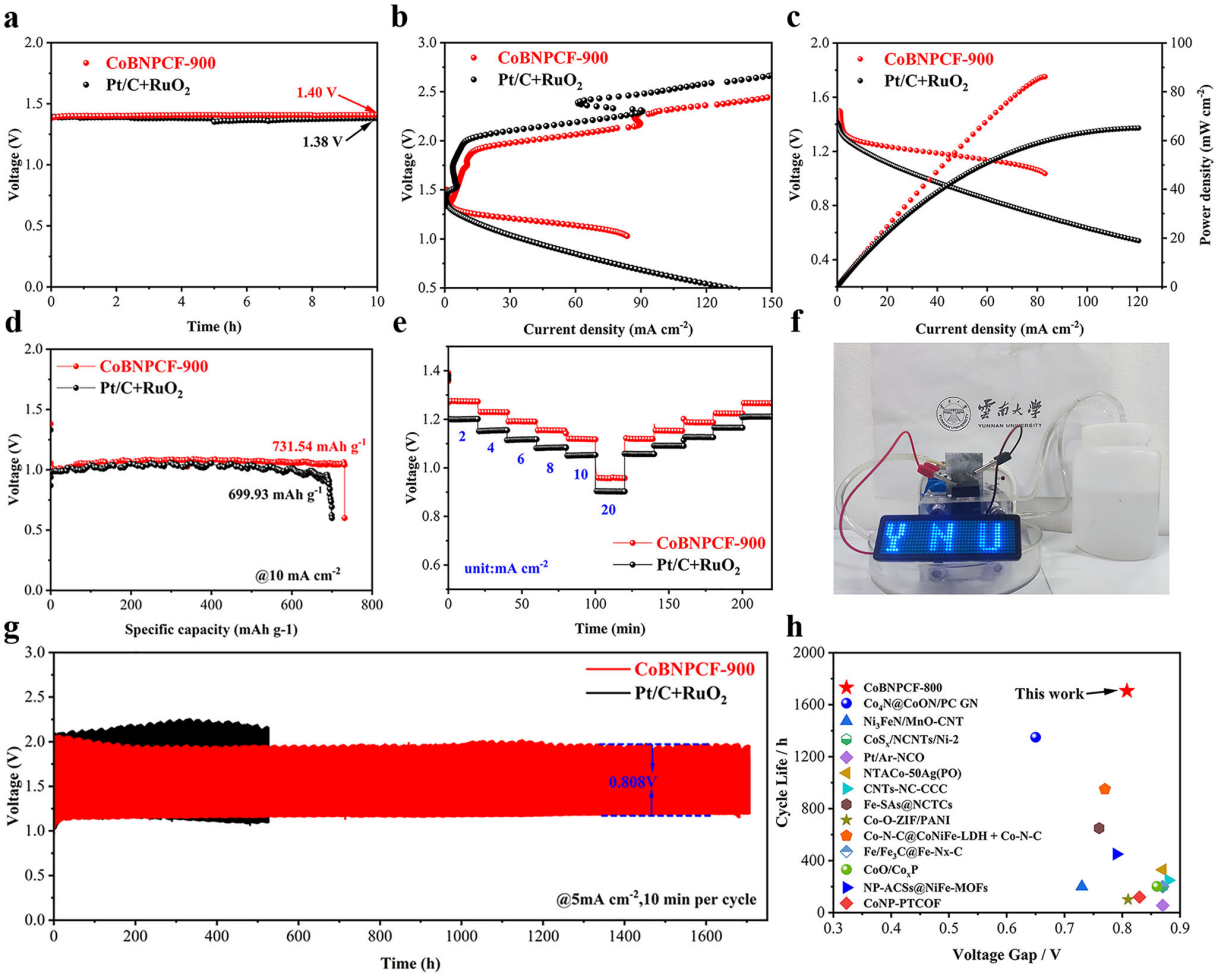
The assembled zinc‐air batteries of CoBNPCF‐900 and RuO_2_+Pt/C: a) Open circuit voltage. b) Charge and discharge curve. c) Power density curves. d) galvanostatic discharge curves. e) Rate‐capability at 2, 4, 6, 8,10, and 20 mA cm^−2^. f) LED light based on ZAB using CoBNPCF‐900. g) Charge and discharge stability plot at a current density of 5 mA cm^−2^. h) Zn‐air battery performance comparison of CoBNPCF‐900 with recently reported zinc‐air battery.

Owing to the remarkable bifunctional ORR/OER performance of CoBNPCF‐900, a fabricated zinc‐air battery with the CoBNPCF‐900 serves as the air cathode and zinc plate as anode to assess its practicability in energy devices. The open circuit voltage of the assembled zinc‐air battery is 1.40 V (**Figure** **7**a), which is 20 mV higher than that of the Pt/C+RuO_2_ electrode. Meanwhile, the voltage gap between the charge and discharge curves of CoBNPCF‐900 is significantly smaller than that of the Pt/C+RuO_2_ electrode with increased current density (Figure 7b), suggesting a higher output voltage and electrochemical stability. Figure 7c depicts the CoBNPCF‐900‐based zinc‐air battery has a peak power density of 86.18 mW cm^−2^, which is superior to the power density of 65.13 mW cm^−2^ of the Pt/C+RuO_2_ electrode. The discharge‐specific capacity of the CoBNPCF‐900‐based zinc‐air battery is 731.54 mAh g^−1^ at a constant current discharge of 10 mA cm^−2^, which is also better than that of the Pt/C+RuO_2_ electrode (699.93 mAh g^−1^), indicating its great potential for operable secondary power sources (Figure 7d). Figure 7e shows the actual discharge voltage recorded at various current densities (2, 4, 6, 8, 10, and 20 mA cm^−2^), in which the CoBNPCF‐900 based zinc‐air battery demonstrates a higher charge‐discharge voltage than that of the Pt/C+RuO_2_ electrode during the various current density (even at a high current density of 20 mA cm^−2^), suggesting its high practicability again. Furthermore, a single CoBNPCF‐900‐based zinc‐air battery could drive an LED screen, demonstrating its applicability in the application prospect (Figure 7f). Significantly, the CoBNPCF‐900‐based zinc‐air battery shows an ultralong stability for 1706.6 h at a charge‐discharge cycling of 5 mA cm^−2^, with only a 0.808 V voltage gap (Figure 7g), which exhibits the superior cycle stability and a small voltage gap compared to the most recently reported RZABs (Figure 7h and Table S8, Supporting Information).

## 3 Conclusion

In summary, a bicontinuous constructed cobalt active centers/asymmetric B/N‐coordination carbon catalyst was developed, which serves as a highly efficient and robust catalyst for catalyzing ORR and OER in alkaline media. Based on this, an ultra‐efficiency and ultra‐stability air cathode was fabricated for rechargeable ZABs. The 3D reconstruction visualization of CoBNPCF‐900 reveals abundant active sites and reaction regions for catalysis, also boosts mass and electron transfer. Simulations of the absolute permeability experiment indicated that the catalytic active site was highly exposed to the liquid electrolyte, guaranteeing a high catalytic efficiency. Equally, DFT calculation further validates that the spin‐polarized Co serves as the primary catalytic active center. The formation of Co@BNC strengthens the electronic coupling at the interface, which is conducive to the adsorption and activation of molecular oxygen during OER and ORR reactions. The advanced catalyst demonstrates a catalytic reversibility of the oxygen redox reactions with a 0.66 voltage gap. Notably, the ZAB assembled with CoBNPCF‐900 air cathode exhibits outstanding cycle stability (1706.6 h) and a narrow voltage gap (0.808 V), suggesting the promising application prospects of the catalysts. This study not only provides new insights into the construction of catalysts featuring 3D interfaces but also offers robust references for the advancement of energy storage and conversion electrocatalysts with practical applications.

## 4 Experimental Section

### Synthesis of the Cobalt Acetate [Co(AC)_2_] / Hydroxy Benzene Boronic Acid [HBBA]/ Polyvinylpyrrolidone [PVP] Precursor Membranes

Figure 1a displays the synthesis procedure, first, 3.0 g of HBBA and 9.0 g of PVP powders were added into 90 mL of N, N‐dimethylformamide [DMF] to obtain the HBBA‐PVP‐DMF solution. Then, 3.8 g Co(AC)_2_·4H_2_O was dissolved in the HBBA‐PVP‐DMF solution and whisked for 8 h to produce a uniformly distributed precursor solution. Finally, weaved the HBBA‐PVP‐DMF‐Co(AC)_2_ solution into Co(AC)_2_ /HBBA/PVP precursor membranes using electrospinning technique. For electrospinning, the flow rate of precursor solution was set at 5 mL h^−1^ and the voltage was 22 kV.

### Synthesis of the Porous CoBNPCFs‐T (T = 600, 700, 800, 900, 1000 °C) Nanofibers

The Co(AC)_2_ /HBBA/PVP precursor membranes were first preoxidation at 270 °C for 2 h in air, then ultrafast flash joule heating was used to pyrolyzed the preoxided‐ Co(AC)_2_ /HBBA/PVP precursor membranes for 5 min at 600, 700, 800, 900, 1000 °C, respectively. Use carbon boat to collect preoxided‐precursor membranes, and the voltage range was set maximum at 200 V, setting current value at 40 A. The voltage and current values were automatically regulated within a preset range to achieve the appropriate temperature.

### 3D Tomograph Reconstruction and Absolute Permeability Experiment Simulations

A series of HAADF images and corresponding EDS mapping images collected by STEM tomography was imported into Inspect 3D software (Thermo Fisher). Use Gauss filter to get precise samples slices of the obtained images and EDS results in all directions. Then, examine each slice and adjust tilt axis and stack alignment through five iterations, to ensure the valid of tilt axis before reconstructing the volume. Then reconstructed the model based on the examined slices of both HAADF and EDS results. The resulted model data was further imported into Avizo software (Thermo Fisher) to get a rendered model.

## Conflict of Interest

The authors declare no conflict of interest.

## Supporting information



Supporting Information

Supplemental Movie 1

Supplemental Movie 2

Supplemental Movie 3

Supplemental Movie 4

## Data Availability

The data that support the findings of this study are openly available in Advanced Science at https://doi.org/10.1002/advs.202514619, reference number 1.
