# Supplementary material for: Desirable Pore Connectivity Effects in Multiscale Interactive Cobalt Centers with Asymmetric B/N‐Coordination Carbon for Promoting Zn‐Air Batteries
Source: Adv Sci (Weinh). 2025 Oct 16;12(48):e14619. doi: 10.1002/advs.202514619 (PMC12752573; doi:10.1002/advs.202514619)
Supplement: Supplementary file 1 — Supporting Information [file ADVS-12-e14619-s002.docx]

Supporting Information

**Desirable Pore Connectivity Effects in Multiscale Interactive Cobalt Centers with Asymmetric B/N-Coordination Carbon for Promoting Zn-Air Batteries**

Tingzheng Fu ^a,#^, Hongbiao Xiao ^a,#^, Qiusen Liu ^a^, Ye Yu ^a^, Zhiqing Che ^a^, Yixing Zhang ^a^, Anran Chen ^ab^*, Mian Li ^c^*, Tingting Liu ^ab^*.

^a^ School of Materials and Energy, Yunnan University, No. 2, Green Lake North Road, Kunming 650091, PR China.

^b^ Electron Microscopy Center, Yunnan University, No. 2, Green Lake North Road, Kunming 650091, PR China

^c^ National and Local Joint Engineering Center for Lithium-ion Batteries and Materials Preparation Technology, Faculty of Metallurgical and Energy Engineering, Kunming University of Science and Technology, Kunming 650093, PR China.

E-mail: anran@ynu.edu.cn, mianzi2009@126.com, liutt133@ynu.edu.cn.

^#^ Tingzheng Fu and Hongbiao Xiao contributed equally to this work.

**Methods**

**1.Chemicals and apparatus**

Chemicals in this work were bought from Shanghai Macklin Biochemical Technology Co., Ltd. including cobalt acetate [Co(AC)_2_], N, N-dimethylformamide [DMF], polyvinylpyrrolidone [PVP], hydroxy benzene boronic acid [HBBA], potassium hydroxide [KOH], commercial RuO_2_, and commercial 20 wt% Pt/C. All chemicals without any further purification. Use ultrapure water to wash and prepare the 0.1 M and 1 M KOH electrolyte aqueous. The purity N_2_ (99.99%) and O_2_ (99.9%) were bought from Kunming Pengyida Gas Products Co. Ltd. Electrochemical tests were conducted by CHI 760E electrochemical workstation (CHI Instruments, Inc., Shanghai) and RRDE-3A Rotating Ring Disk Electrode Apparatus Ver.3.0 (Japan). Ultrafast flash joule heating (FJH) was performed in CIS-JH3.3-P (Hefei In-situ Technology. Co., Ltd.).

**2.Physical characterization of the precursor membranes and resultant CoBNPCFs-T nanofibers**

For the physical characterization of precursor network and series of CoBNPCFs-T nanofibers, the scanning electron microscopy (SEM) was characterized using a field emission Zeiss Gemini 500 at 3 kV. The transmission electron microscopy (TEM), high-angle annular dark field scanning transmission electron microscopy (HAADF), electron dispersive spectroscopy (EDS) analysis, scanning transmission electron microscopy (STEM) and BF-S images were performed by Talos F200X (Thermo Fisher scientific). Spherical Aberration Corrected Transmission Electron Microscope (AC-TEM) images were acquired by Spectra 300 (Thermo Fisher scientific). The representation of wide-angle X-ray diffraction (XRD) was using Smart Lab SE (Rigaku) operated at 40 kV with Cu-Kα radiation with λ=1.54 Å. Using Quantachrome Instruments (version 6.0) to perform the N_2_ adsorption-desorption isotherms. Before characterization, all samples were degassed in vacuum at 300 ℃ for 6 h. The BET specific surface areas were performed by Brunauer-Emmett-Teller (BET) method through absorption data. Use the Barrett-Joyner-Halenda (BJH) model to analysis the pore size distribution data by adsorption branches. The Raman spectroscopy patterns was characterized with a Renishaw Invia Raman spectrometer. The chemical valence analysis of samples using X-ray photoelectron spectroscopy (XPS) on a K-Alpha+ (Thermo Fisher) spectrometer. The binding energies were corrected with respect to the containment carbon (C1s 284.8 eV). The water and bubble contact angles were conducted on SDC-200S and LAUDA Scientific LSA100S-T, respectively.

**3. STEM tomography**

STEM tomography was performed using Talos F200X (Thermo Fisher Scientific) at 200 kV with a model 2021 analytical tomography holder (Fischione instruments). Before loading CoBNPCF-900 catalysts, the specimen holder were plasma-clean for 10 seconds, which can diminish carbon deposition and other contamination. Series of HAADF images and corresponding EDS results were acquired in 2° tilt increments from -70° to 64°. A -90° angle was automatically applied to the specimen during the acquisition.

**4. DFT calculation details**

All the calculations are executed in the framework of the density functional theory with the projector augmented plane-wave method, as implemented in the Vienna ab initio simulation package. Including spin polarization.^[1]^ The generalized gradient approximation (GGA) and Ernzerhof were used for the exchange-correlation potential.^[2]^ The van der Waals (vdW) interactions was described by DFT-D3 scheme of dispersion correction in molecule adsorption.^[3]^ The cut-off energy for plane wave was set to 600 eV. Set the energy criterion at 1E^-05^ eV in iterative solution of the Kohn-Sham equation. All the structures are unwinded until the residual forces on the atoms have decreased to less than 0.02 eV/Å. The electron smearing width of σ = 0.03 eV was employed based on the Gaussian smearing technique. The Brillouin zone integration is characterized by the homogeneity distributed dispersion of going through the Gamma point to select a 2x2x1 k-mesh in the Monkhorst-Pack grid to make structure optimization, and we select 4x4x1 k-mesh to do density of state (DOS) calculations.^[4]^ The charge density difference was evaluated using the formula Δρ = ρ(AB) - ρ(A) - ρ(B), then analyzed by using the VESTA code.^[5]^

**5. Electrochemical characterization of various CoBNPCFs-T (T =600, 700, 800, 900, and 1000 ℃) nanofibers toward OER and ORR in alkaline condition.**

Electrochemical tests were conducted at room temperature in the three-electrode system on the CHI 760E electrochemical workstation (CHI Instruments, Inc., Shanghai). Catalyst-modified rotating disk electrode (RDE; 3 mm in diameter with a geometric area of 0.07065 cm^2^) was used as the working electrode. Counter electrode and reference electrode using Pt wire and Ag/AgCl electrode respectively for both OER and ORR tests. 3 mg of as-composited catalyst powders were severally ultrasonically dispersed in 1 mL Nafion solution (0.5 wt%) for 30 min to produce the uniformly catalyst inks. Then, 10 μL of catalyst-Nafion solution was transferred to the RDE and dried at room temperature.

All potentials displayed in this study were converted to potential referred to the reversible hydrogen electrode (RHE). All potentials concerning Ag/AgCl were calculated through the following **Equation 1**:

$E_{RHE}=E_{\frac{Ag}{AgCl}}+0.059pH+0.197$ (1)

where V is the unit of potential, E_RHE_ is the potential in RHE scale, and E_Ag/AgCl_ is the potential referred to Ag/AgCl electrode. All current density (j) were the specific ratios of the measurement currents to the area of the RDE electrode. All potential data were collected in LSV curves with 90% iR compensation. In OER and ORR tests, Tafel plots resulting from log│j│ and ERHE (from LSV curves). By fitting the linear portions of Tafel plots to the Tafel **Equation 2**, the Tafel slope (b) were get to assess the OER and ORR kinetics.

$Ƞ=blog│j│+a$ (2)

**6. Measurements of Zn-air batteries**

A rechargeable aqueous Zn-air battery was assembled with a 1 mm thick polished zinc plate as the anode and the carbon paper/nickel foam/waterproof membrane composite substrates with the synthesized catalyst as the air cathode. Aqueous solutions of 6 M KOH with 0.2 M Zn(Ac)_2_ were synthesized as electrolytes. Using 400 μL ultrapure water containing 2 mg CoBNPCF-900 catalyst with ultrasonically scattered for 20 min, then coated on the composite substrates (1 cm^2^) and dried at room temperature as air cathode. The recharge and discharge cycle was recorded at a Neware CT-4008 battery testing system.


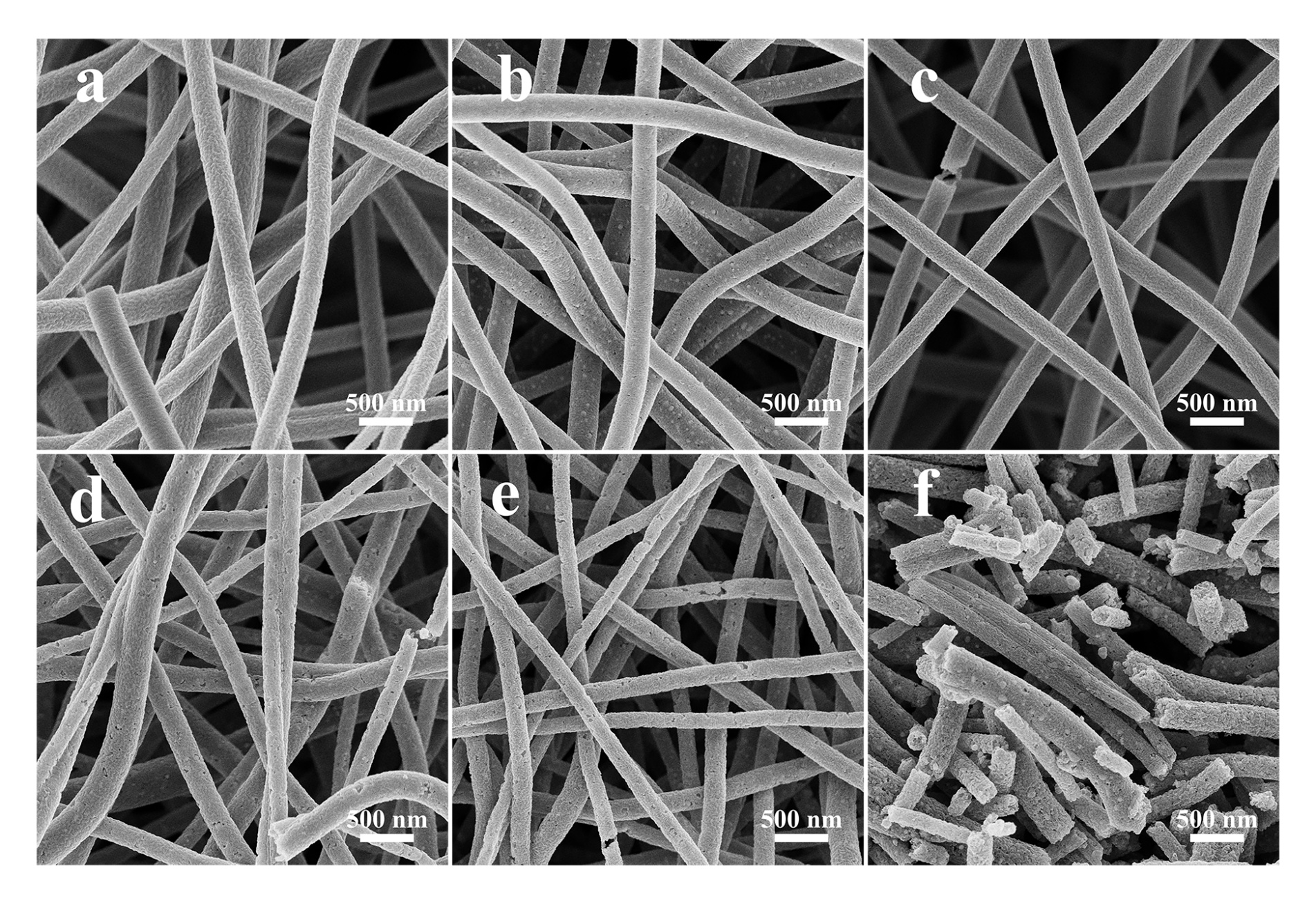


**Figure S1.** SEM images of a) precursor membrane, b) CoBNPCF-600, c) CoBNPCF-700, d) CoBNPCF-800, e) CoBNPCF-900, and f) CoBNPCF-1000.


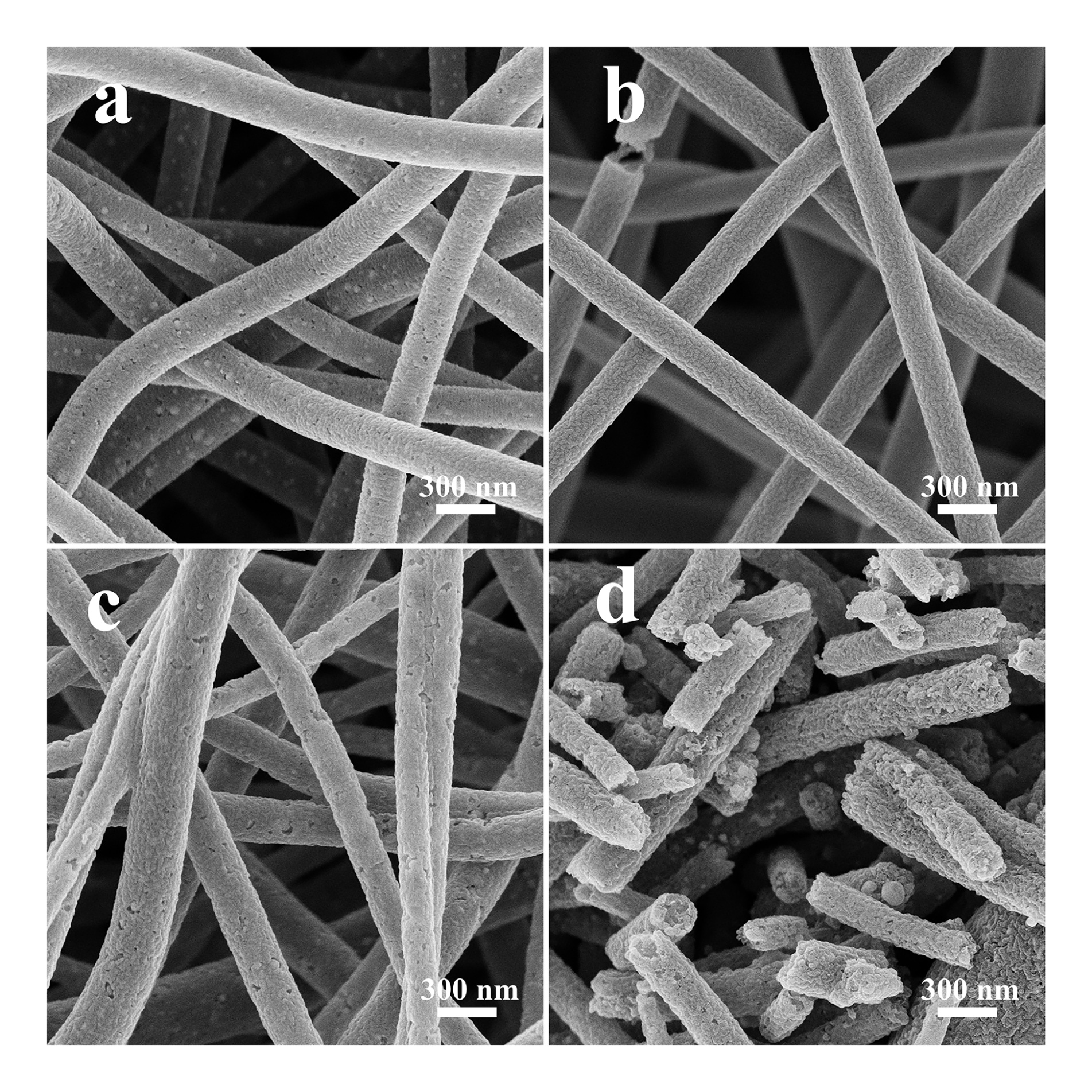


**Figure S2.** SEM images of a) CoBNPCF-600, b) CoBNPCF-700, c) CoBNPCF-800, and d) CoBNPCF-1000.


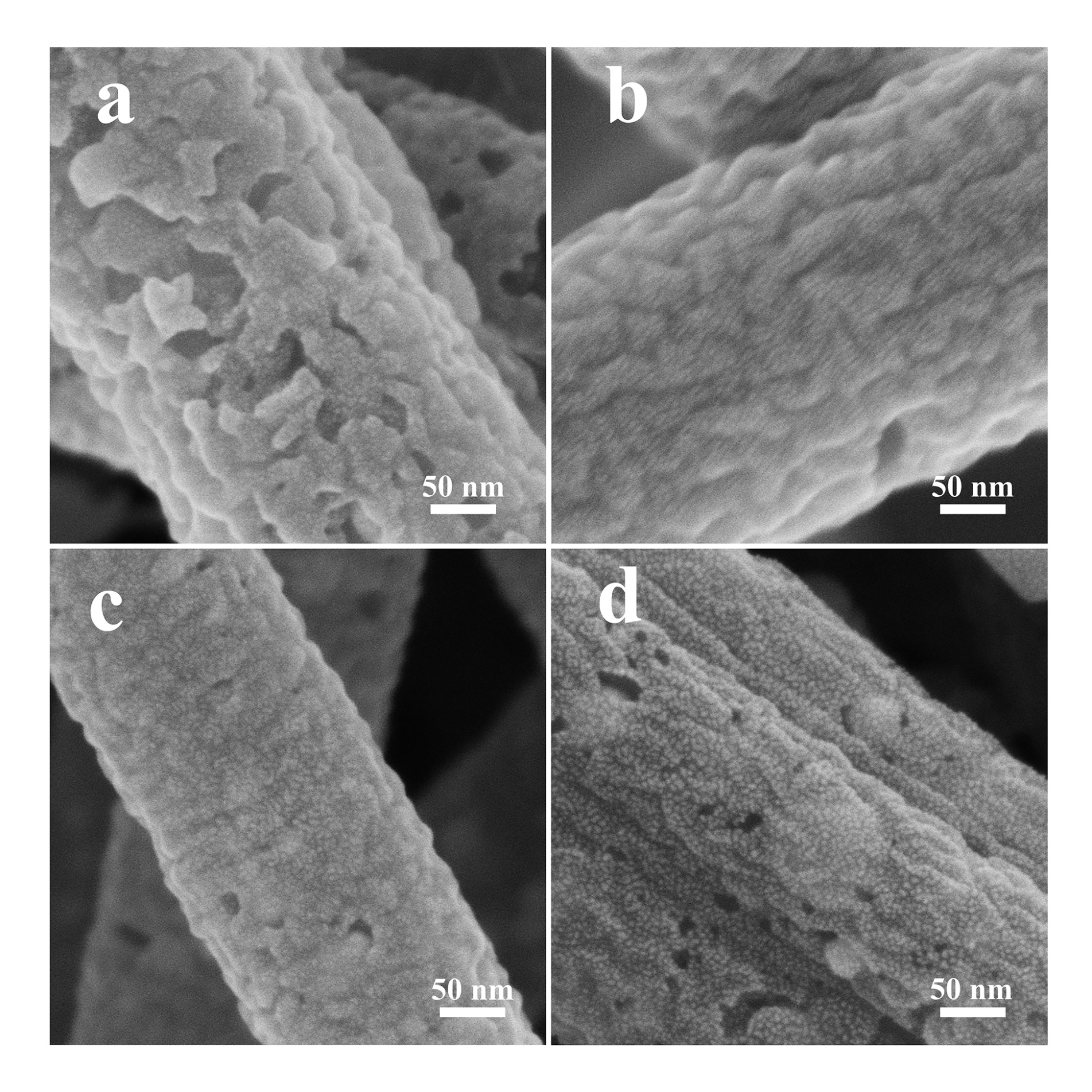


**Figure S3.** High magnificent SEM images of a) CoBNPCF-600, b) CoBNPCF-700, c) CoBNPCF-800, and d) CoBNPCF-1000.


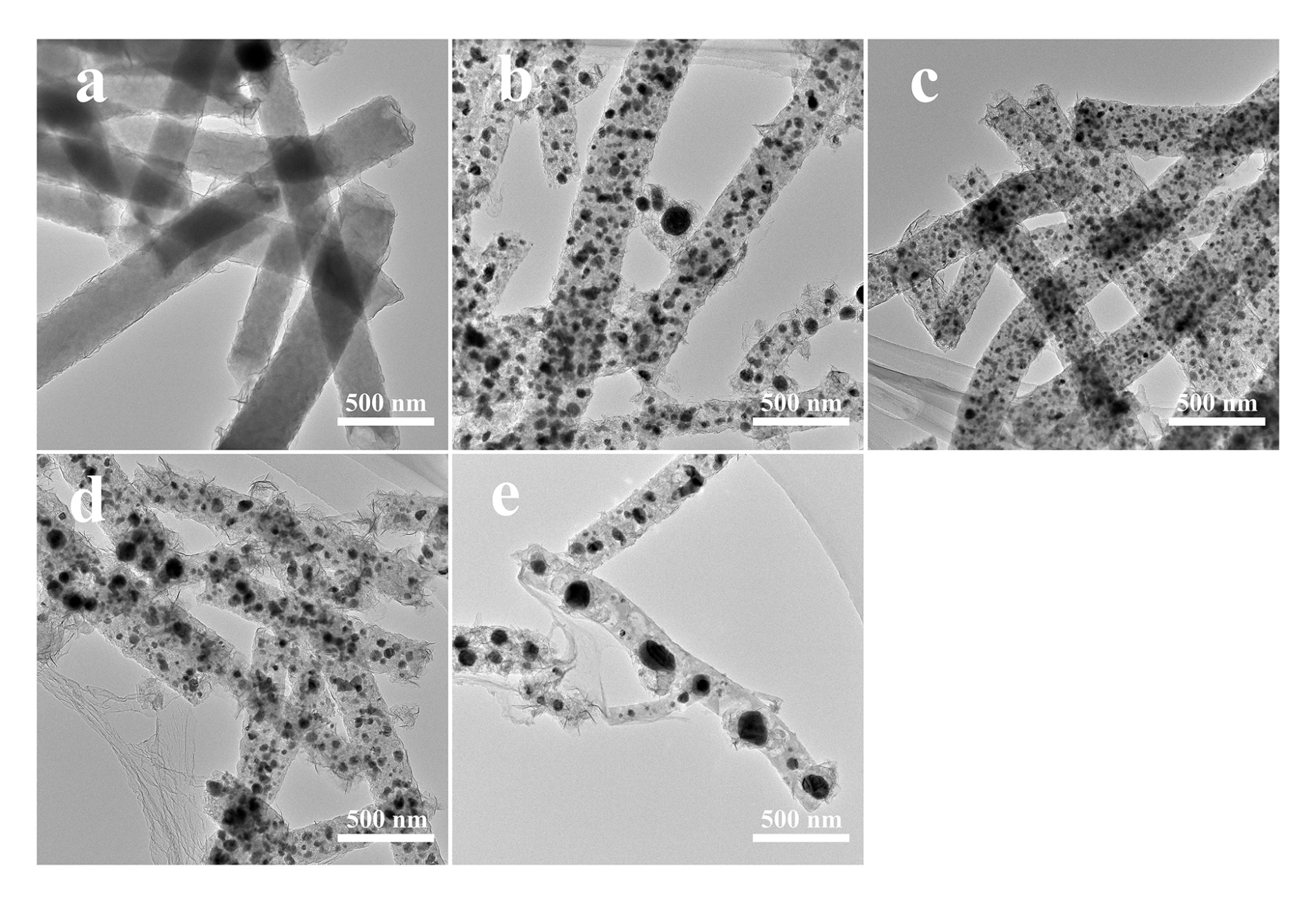


**Figure S4.** TEM images of a) CoBNPCF-600, b) CoBNPCF-700, c) CoBNPCF-800, d) CoBNPCF-900, e) CoBNPCF-900.


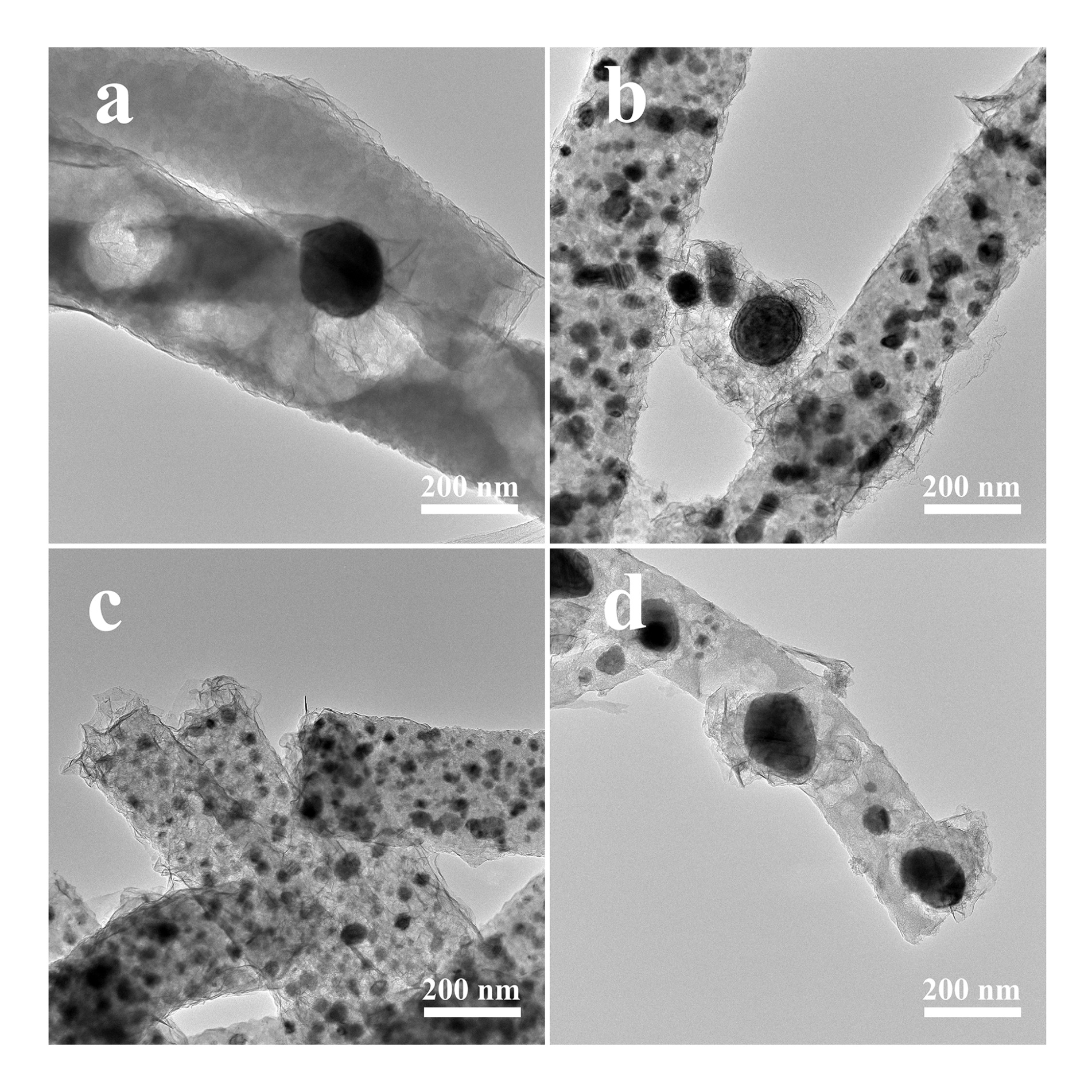


**Figure S5.** TEM images of a) CoBNPCF-600, b) CoBNPCF-700, c) CoBNPCF-800, and d) CoBNPCF-1000.


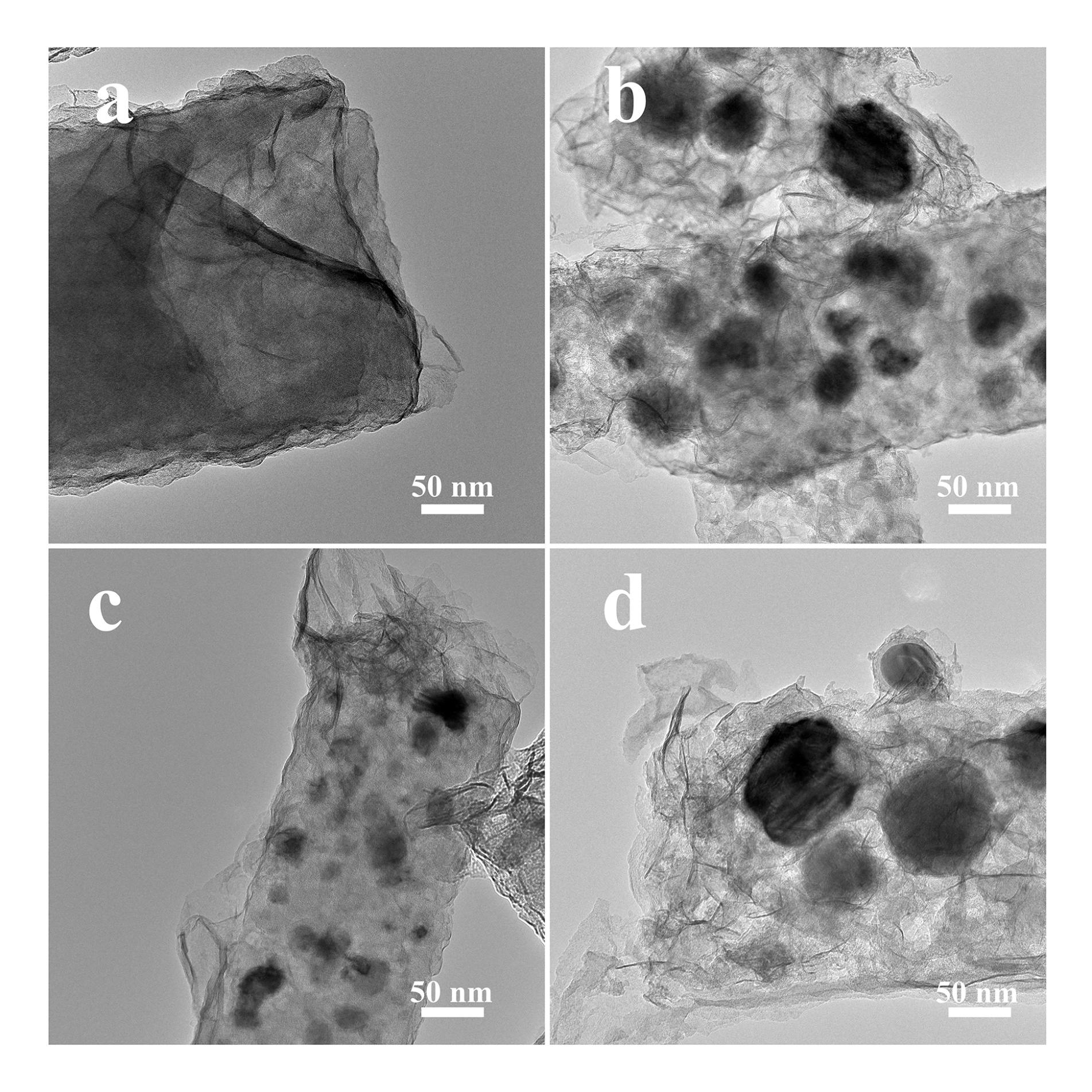


**Figure S6.** High magnificent TEM images of a) CoBNPCF-600, b) CoBNPCF-700, c) CoBNPCF-800, and d) CoBNPCF-1000.


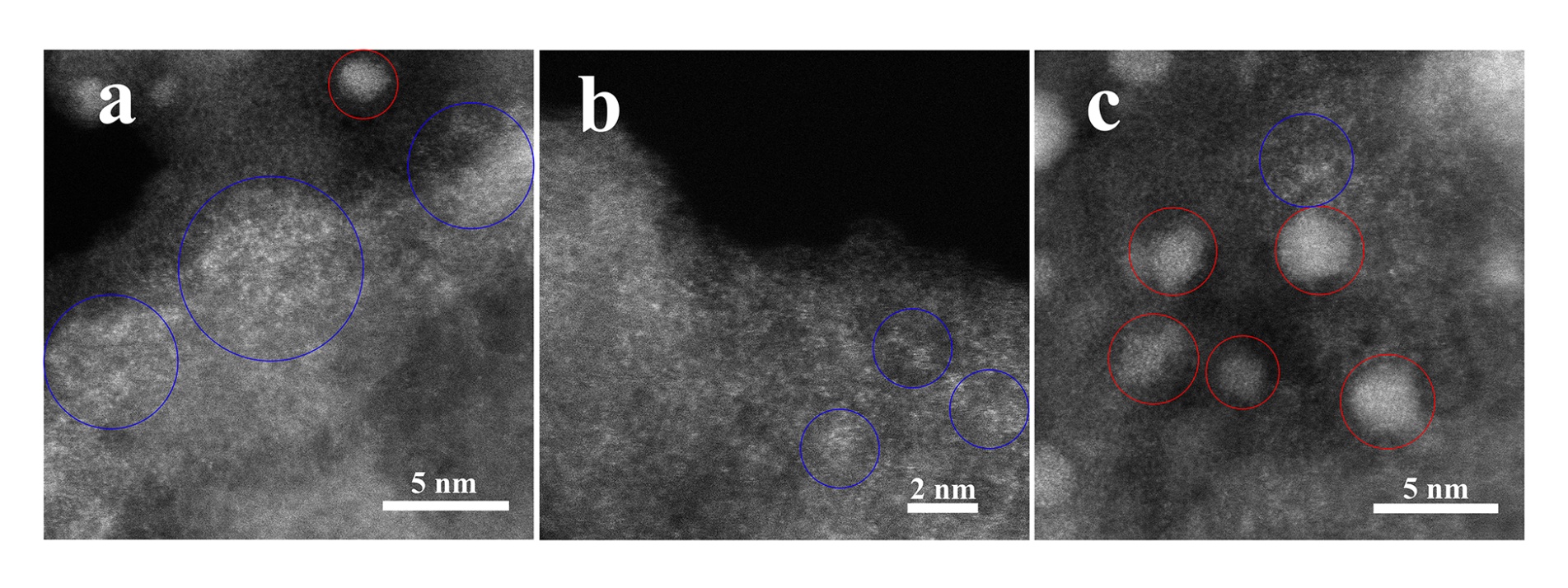


**Figure S7.** AC-TEM images of CoBNPCF-900.


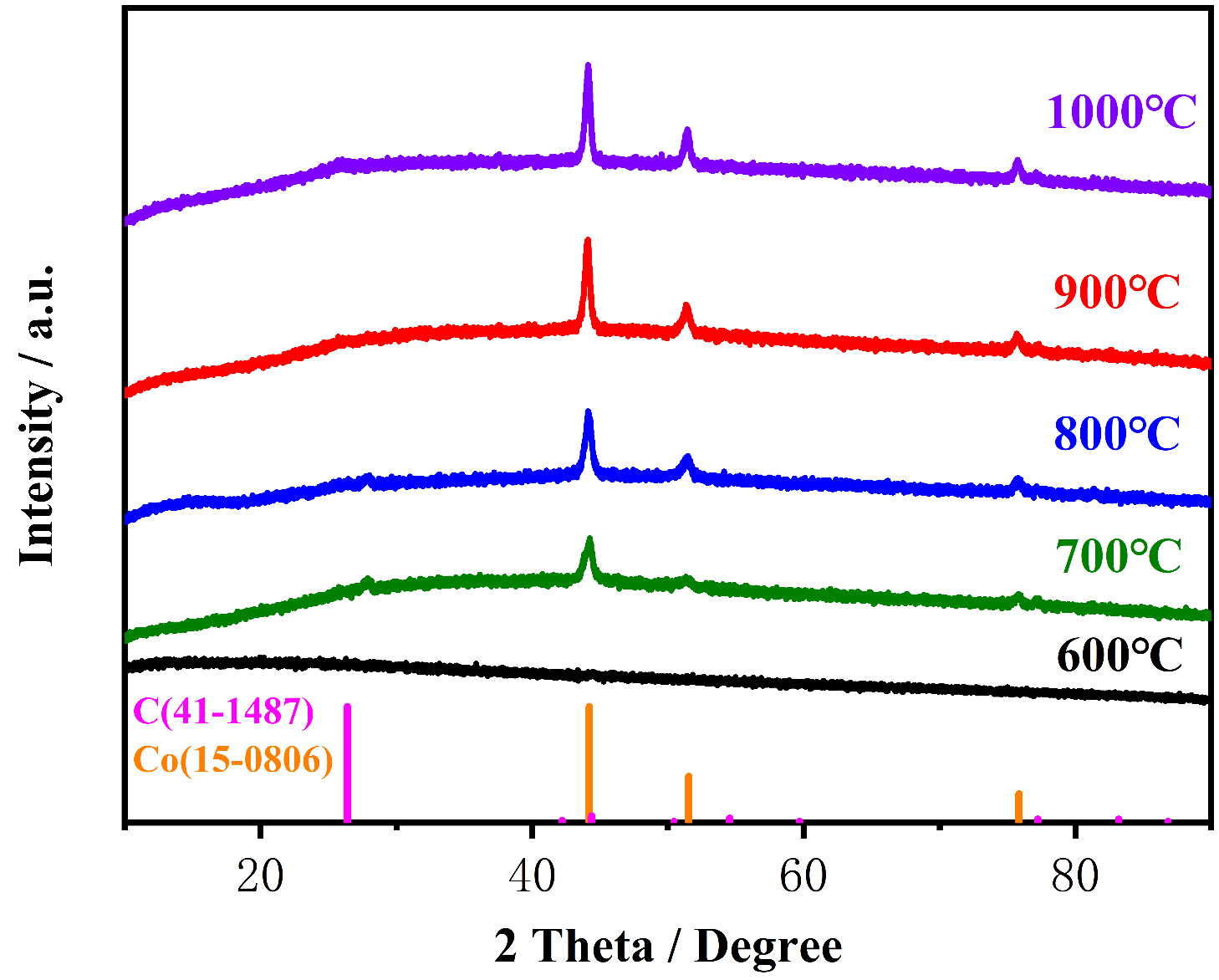


**Figure S8.** XRD patterns of CoBNPCFs-T.


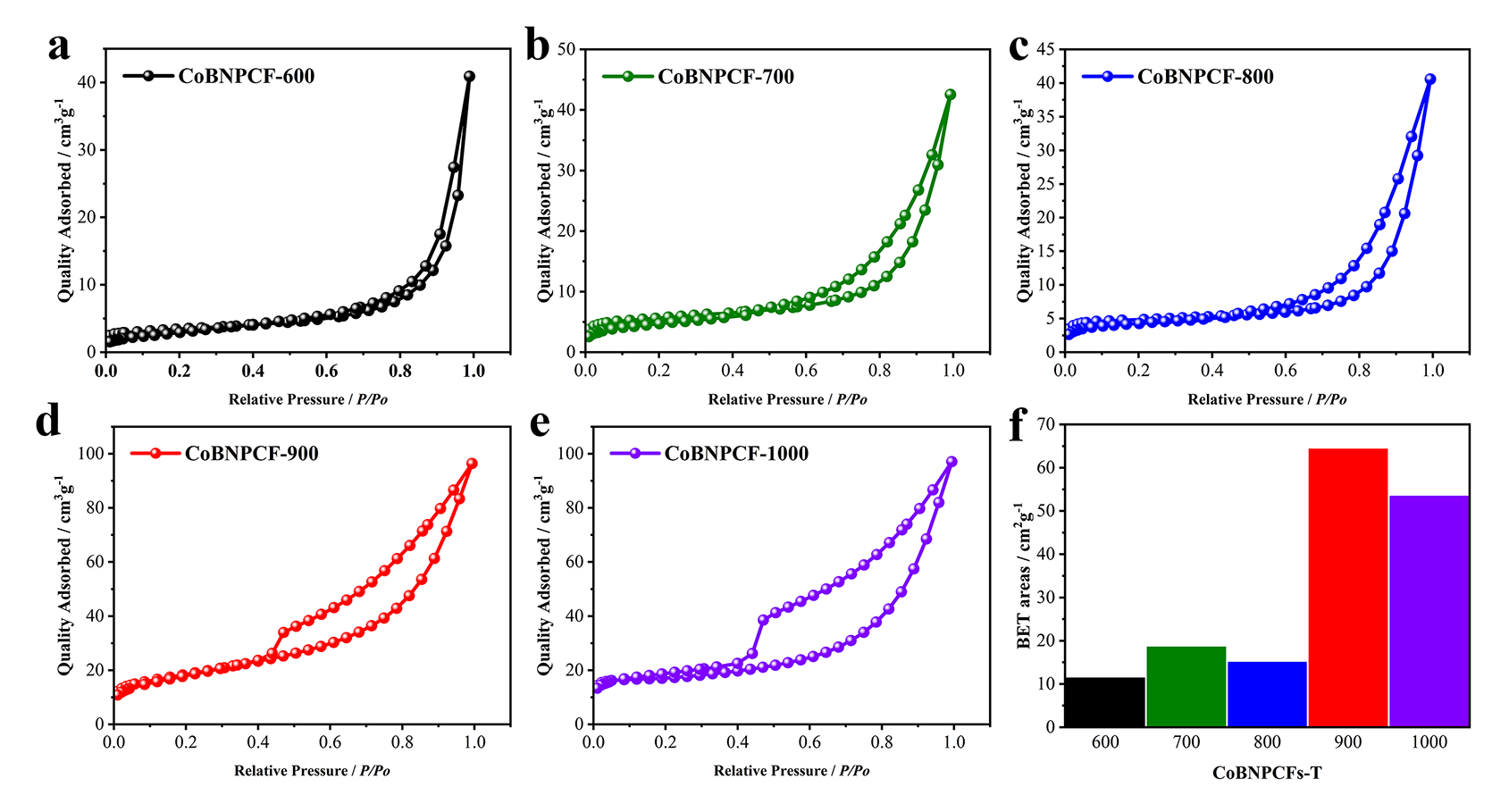


**Figure S9.** N_2_ adsorption-desorption isotherms of a) CoBNPCF-600, b) CoBNPCF-700, c) CoBNPCF-800, d) CoBNPCF-900, e) CoBNPCF-1000, and f) statistic of the BET surface areas.


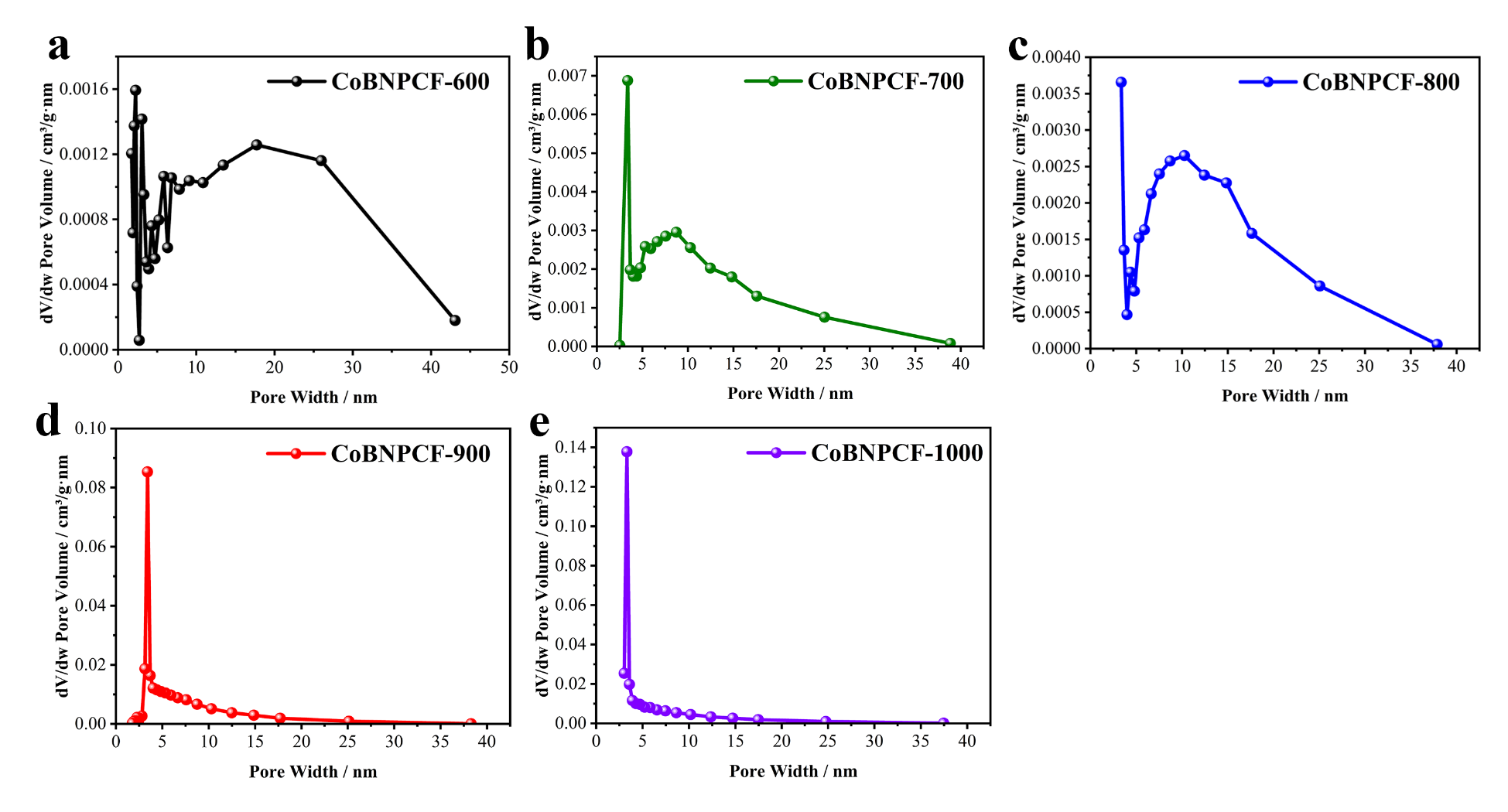


**Figure S10.** Pore size distribution plots of a) CoBNPCF-600, b) CoBNPCF-700, c) CoBNPCF-800, d) CoBNPCF-900, e) CoBNPCF-1000.


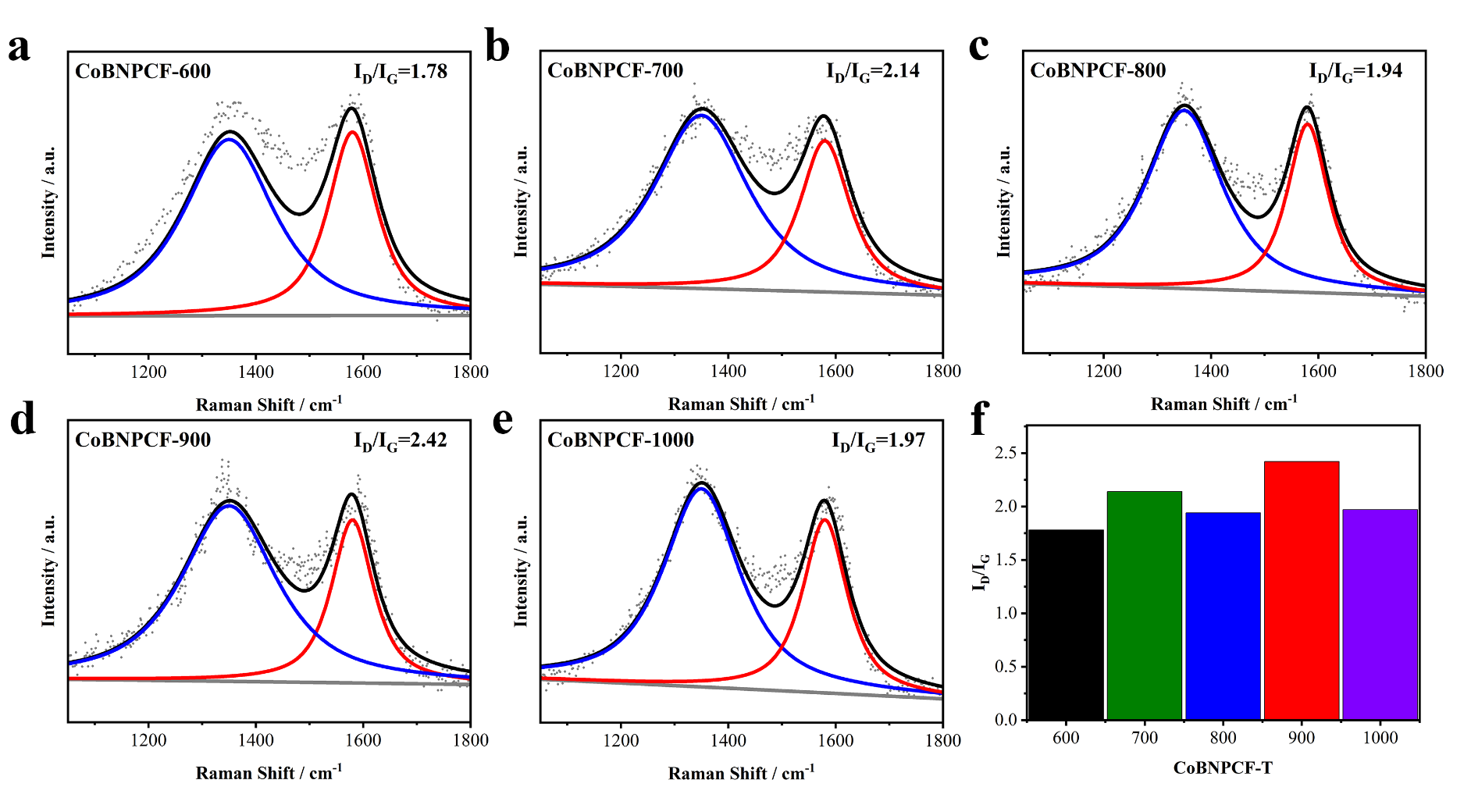


**Figure S11.** Raman spectrum of a) CoBNPCF-600, b) CoBNPCF-700, c) CoBNPCF-800, d) CoBNPCF-900, e) CoBNPCF-1000, and f) statistic of the I_D_/I_G_ values.


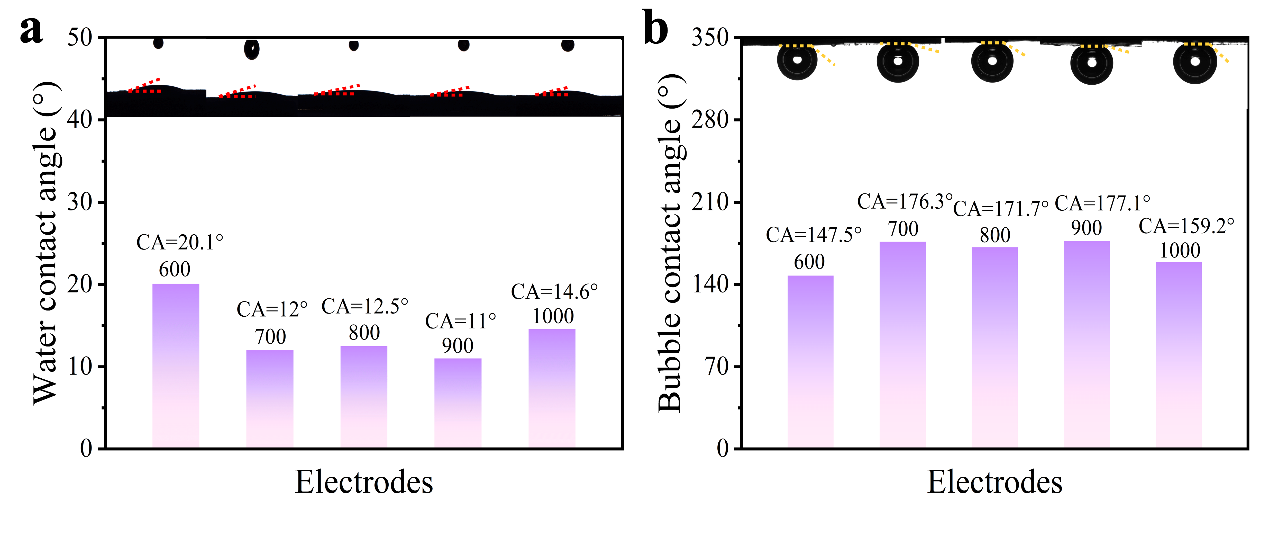


**Figure S12.** (a) The water contact angles, and (b) bubble contact angles of the catalysts.


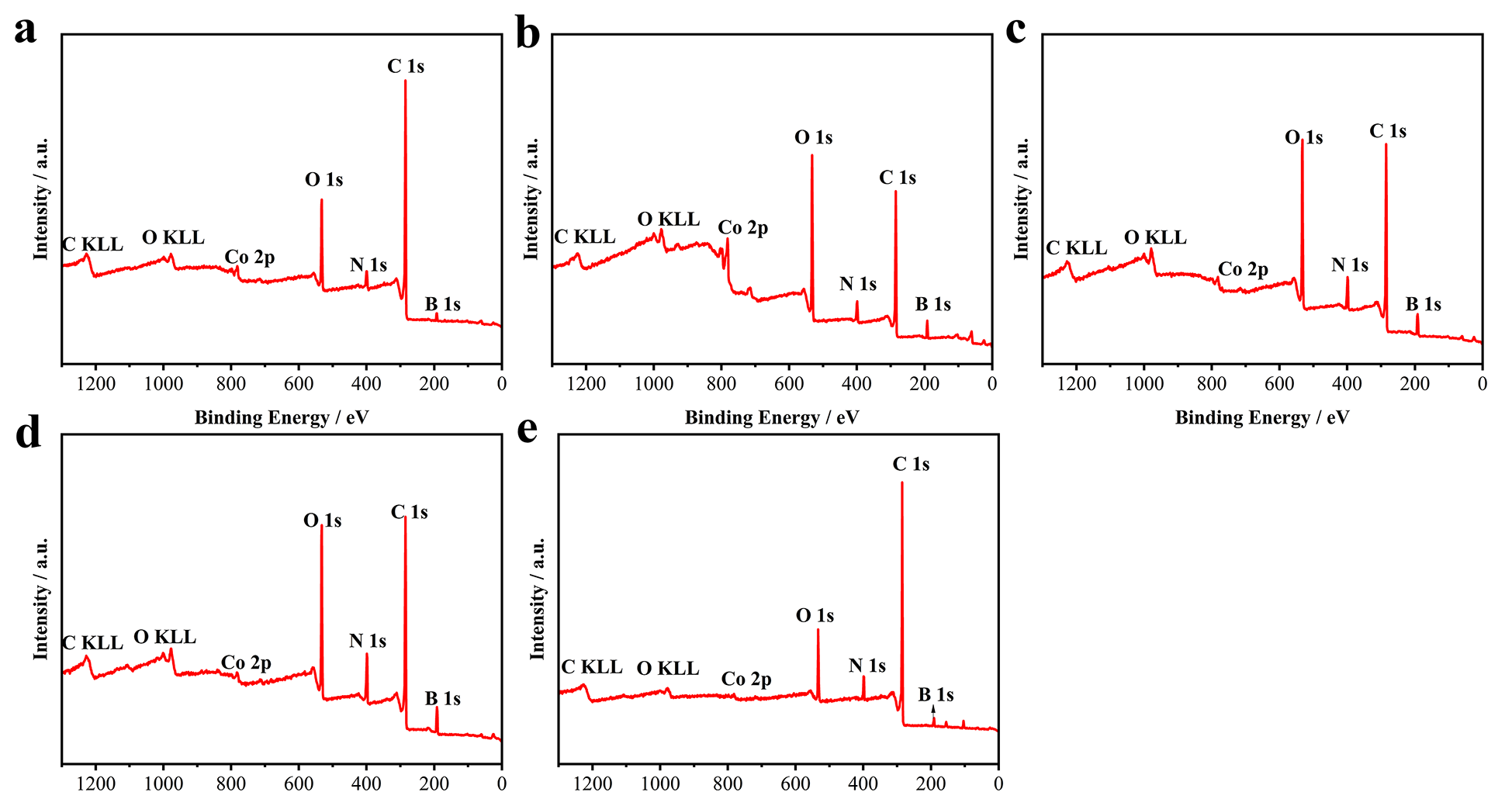


**Figure S13.** XPS survey of a) CoBNPCF-600, b) CoBNPCF-700, c) CoBNPCF-800, d) CoBNPCF-900, e) CoBNPCF-1000


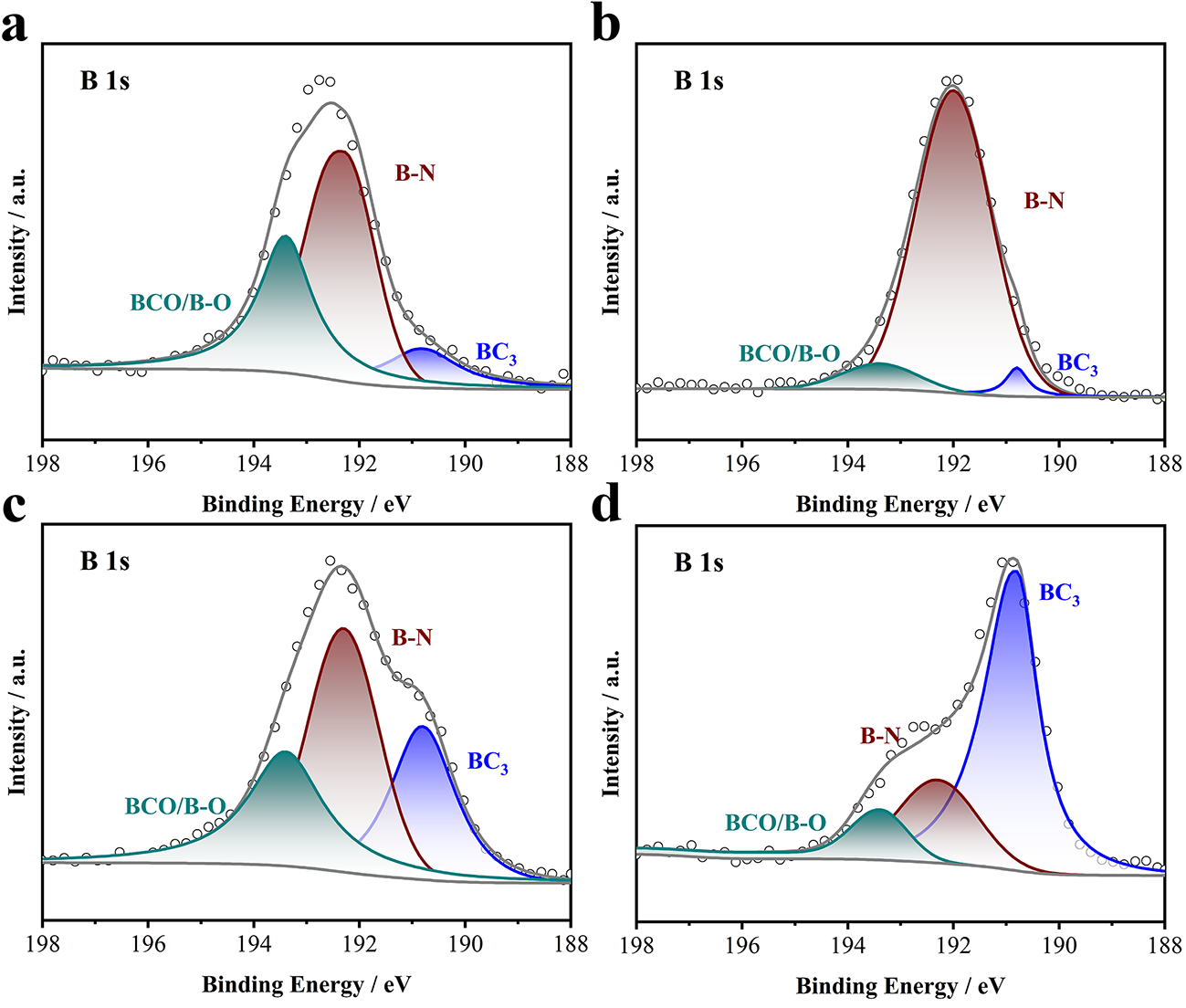


**Figure S14.** High-resolution B1s of a) CoBNPCF-600, b) CoBNPCF-700, c) CoBNPCF-800, and d) CoBNPCF-1000.

**Table S1.** The atomic of various B atoms for CoBNPCFs-T samples.

|  | 600 | 700 | 800 | 900 | 1000 |
| --- | --- | --- | --- | --- | --- |
| Total B | 1.75 | 2.52 | 4.47 | 5.05 | 2.47 |
| BC_3_ | 0.23 | 0.29 | 1.21 | 2.03 | 1.52 |
| B-N | 0.91 | 2.12 | 1.9 | 2.42 | 0.74 |
| B-O/BCO | 0.61 | 0.11 | 1.36 | 0.6 | 0.21 |


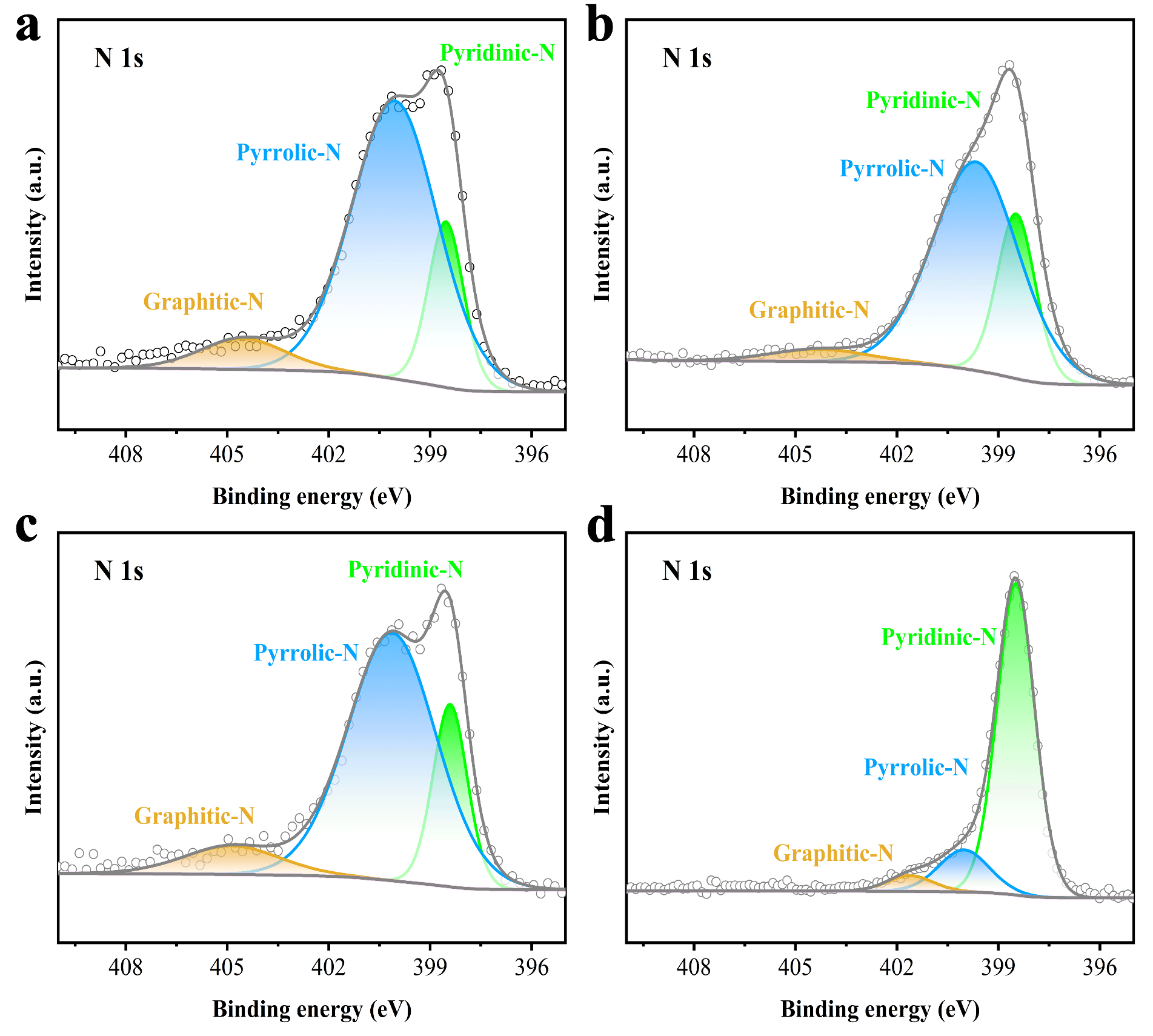


**Figure S15.** High-resolution N1s of a) CoBNPCF-600, b) CoBNPCF-700, c) CoBNPCF-800, and d) CoBNPCF-1000.

**Table S2.** The atomic of various N atoms for CoBNPCFs-T samples.

|  | 600 | 700 | 800 | 900 | 1000 |
| --- | --- | --- | --- | --- | --- |
| Total N | 6.89 | 10.17 | 6.07 | 14.76 | 6.95 |
| Pyridinic-N | 1.25 | 2.52 | 1.23 | 7.27 | 5.56 |
| Pyrrolic-N | 5.07 | 7.17 | 4.30 | 5.72 | 1.05 |
| Graphitic-N | 0.57 | 0.48 | 0.54 | 1.77 | 0.34 |


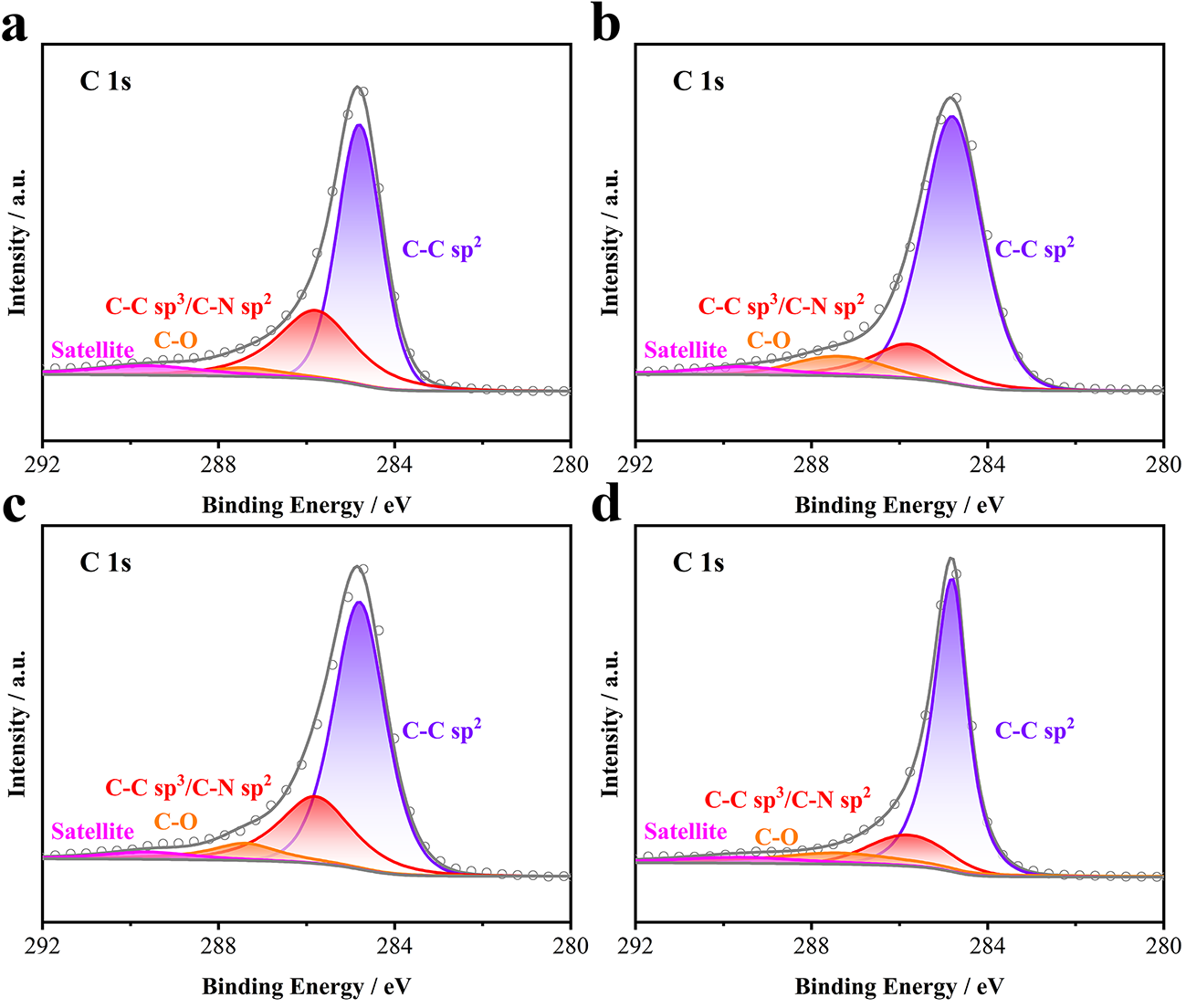


**Figure S16.** High-resolution C1s of a) CoBNPCF-600, b) CoBNPCF-700, c) CoBNPCF-800, and d) CoBNPCF-1000.


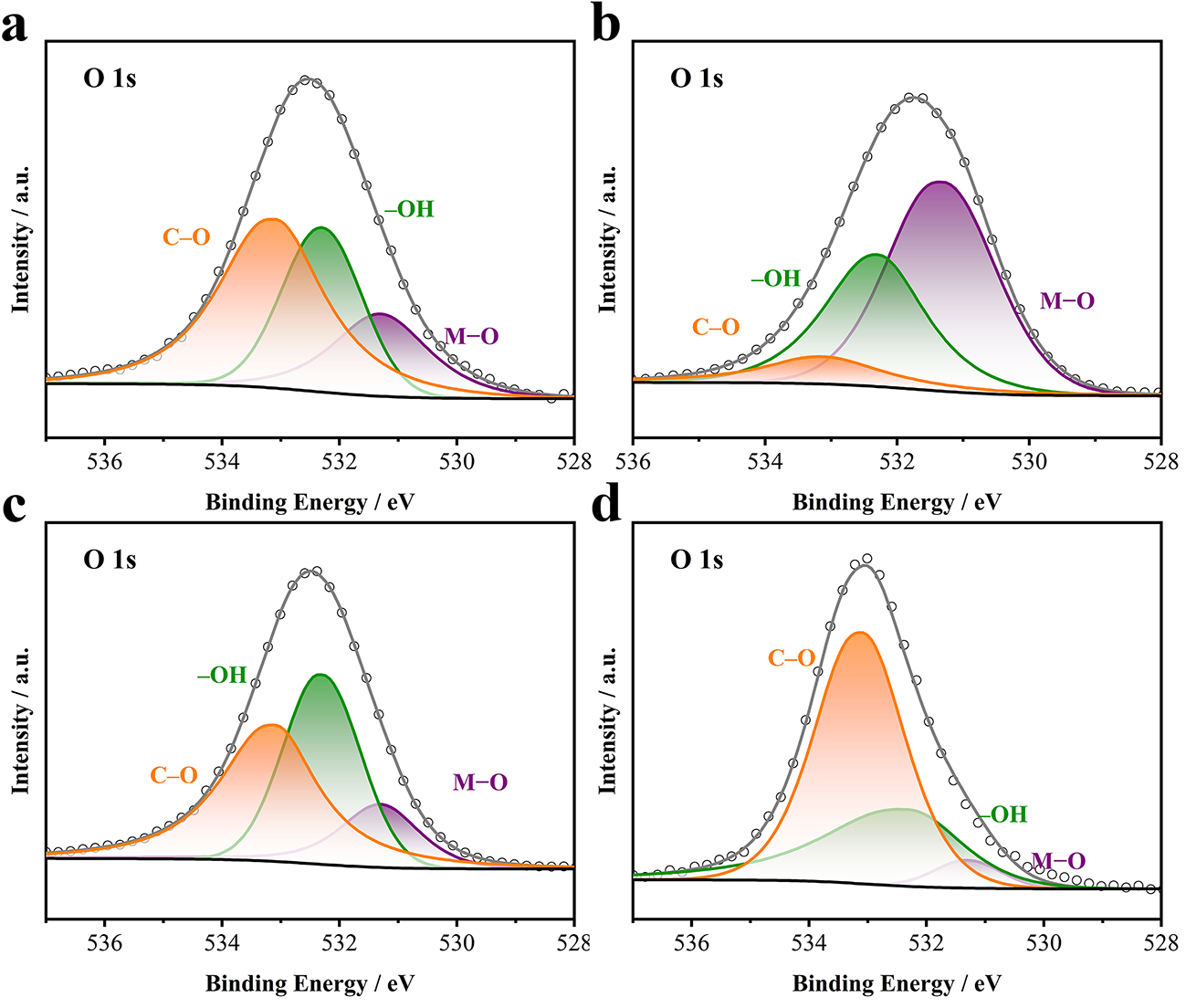


**Figure S17.** High-resolution O1s of a) CoBNPCF-600, b) CoBNPCF-700, c) CoBNPCF-800, and d) CoBNPCF-1000.


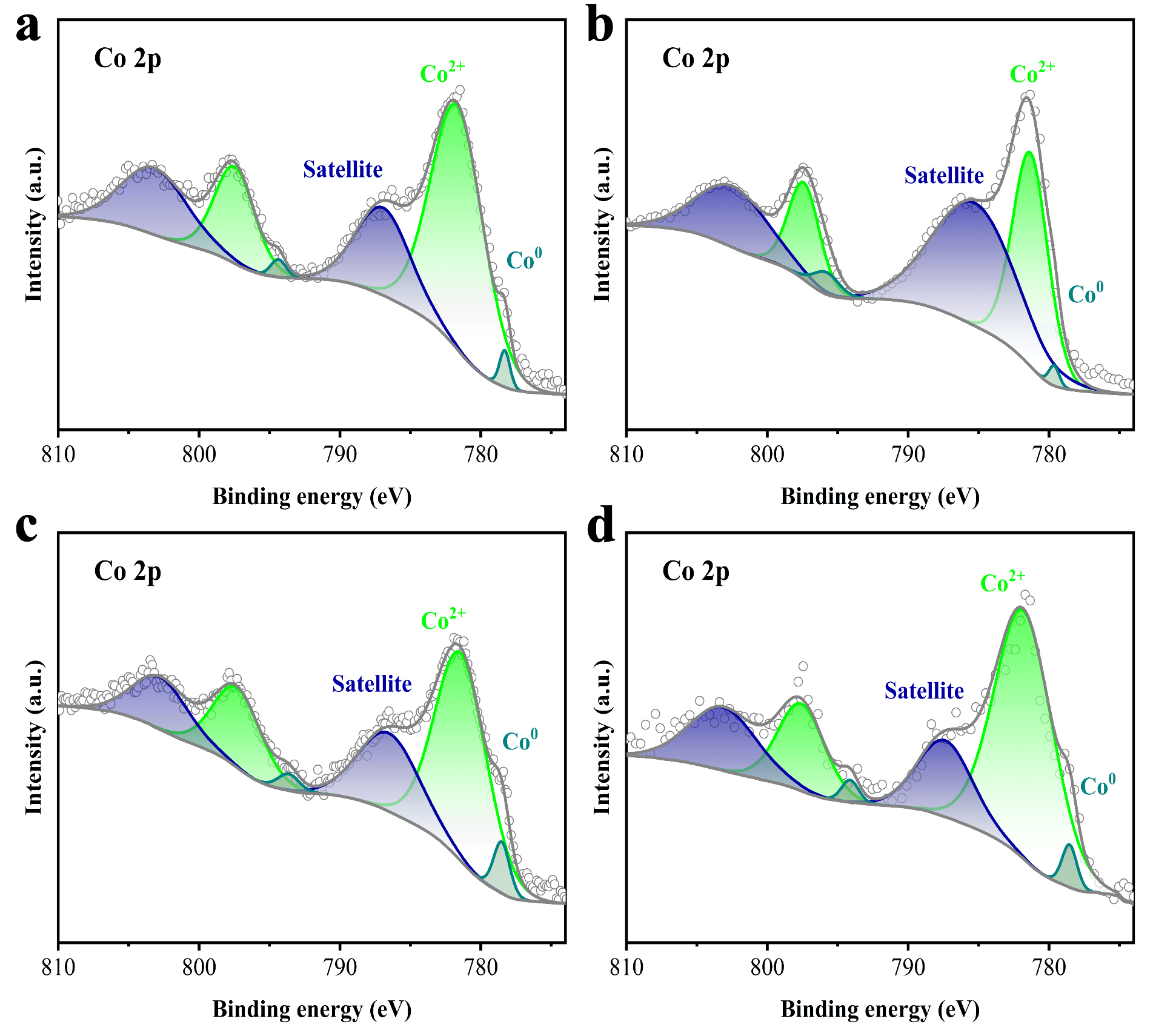


**Figure S18.** High-resolution Co2p of a) CoBNPCF-600, b) CoBNPCF-700, c) CoBNPCF-800, and d) CoBNPCF-1000.


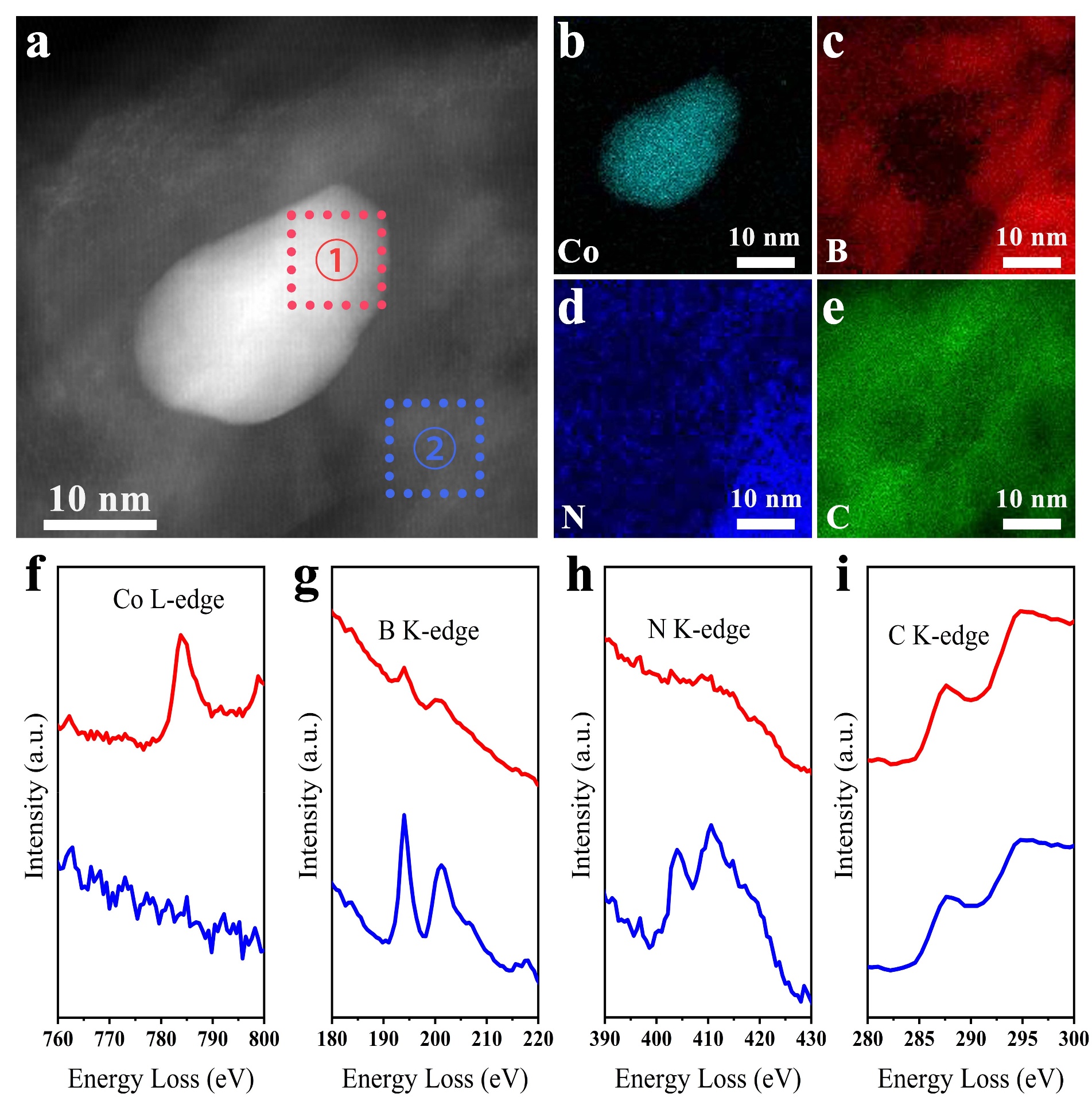


**Figure S19.** a) The EELS spectra for the Co particles (red line) and Co-free region (blue line), b-e) corresponding EDS mapping images, and the f) Co L-edge, g) B K-edge, h) N K-edge, and i) C K-edge of CoBNPCF-900.


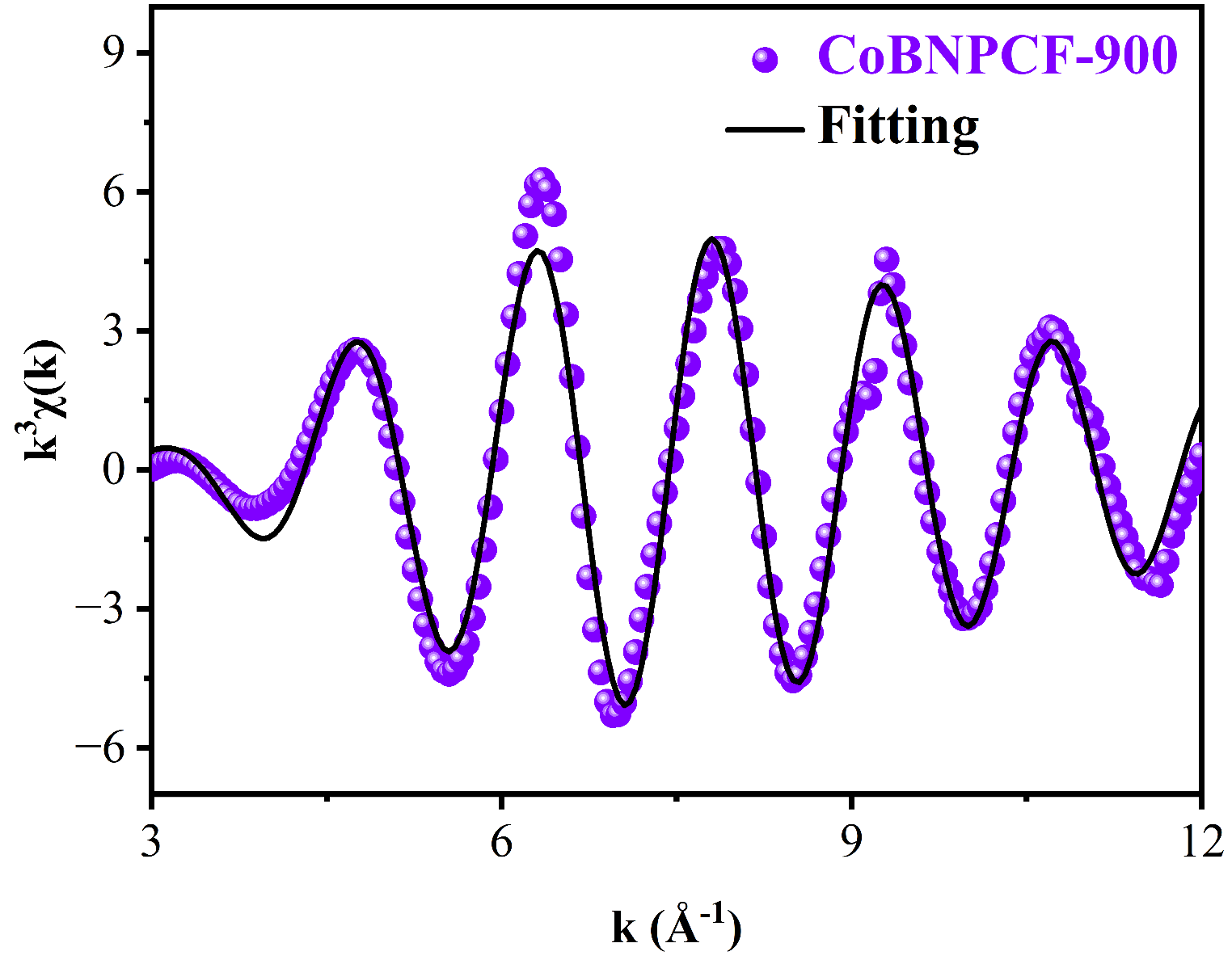


**Figure S20.** Fitting data of K^3^-weighted k-space EXAFS spectra for CoBNPCF-900.


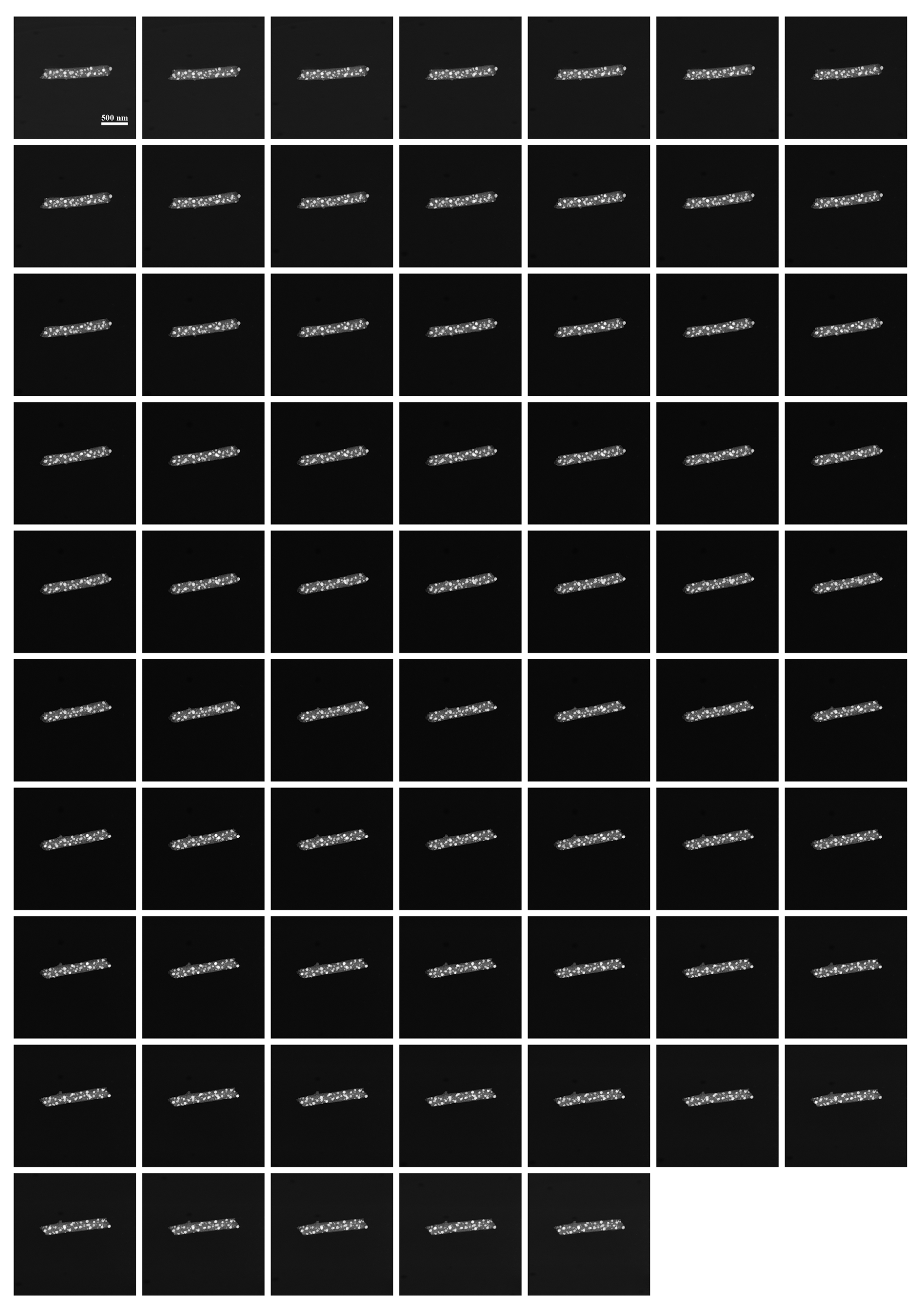


**Figure S21.** STEM tomography images acquired from -70° to 64°.


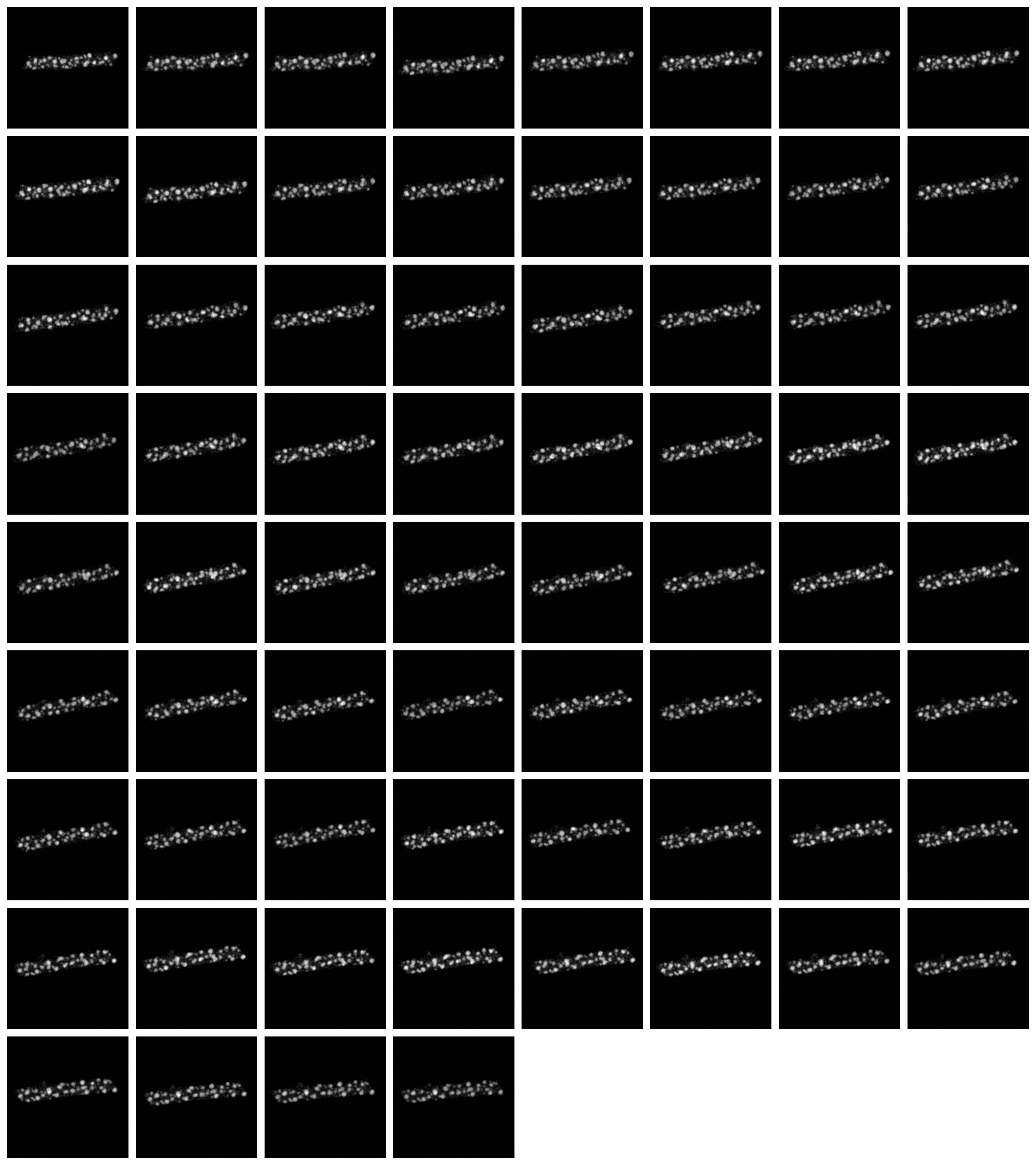


**Figure S22.** Co element tomography images acquired from -70° to 64°.


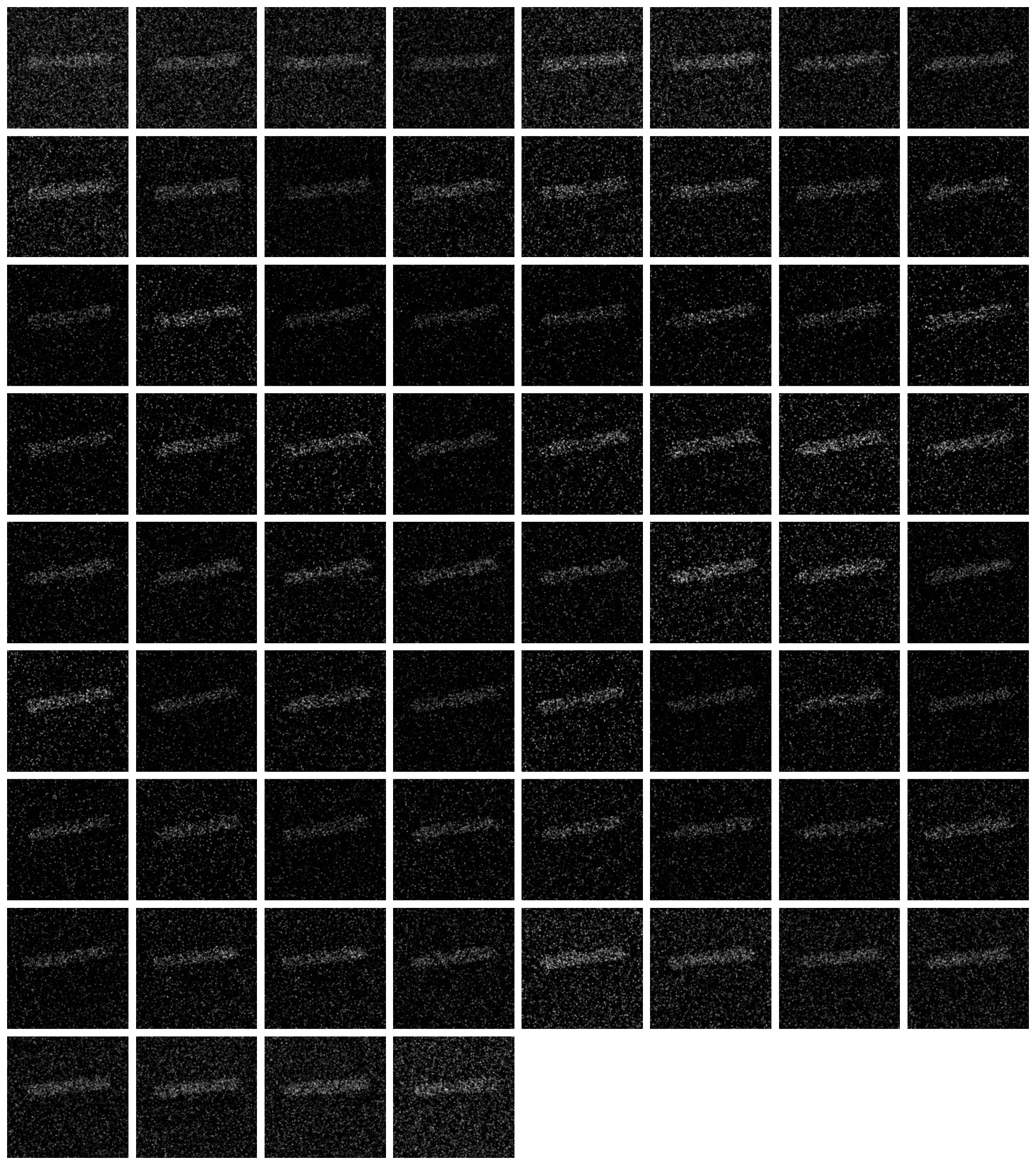


**Figure S23.** B element tomography images acquired from -70° to 64°.


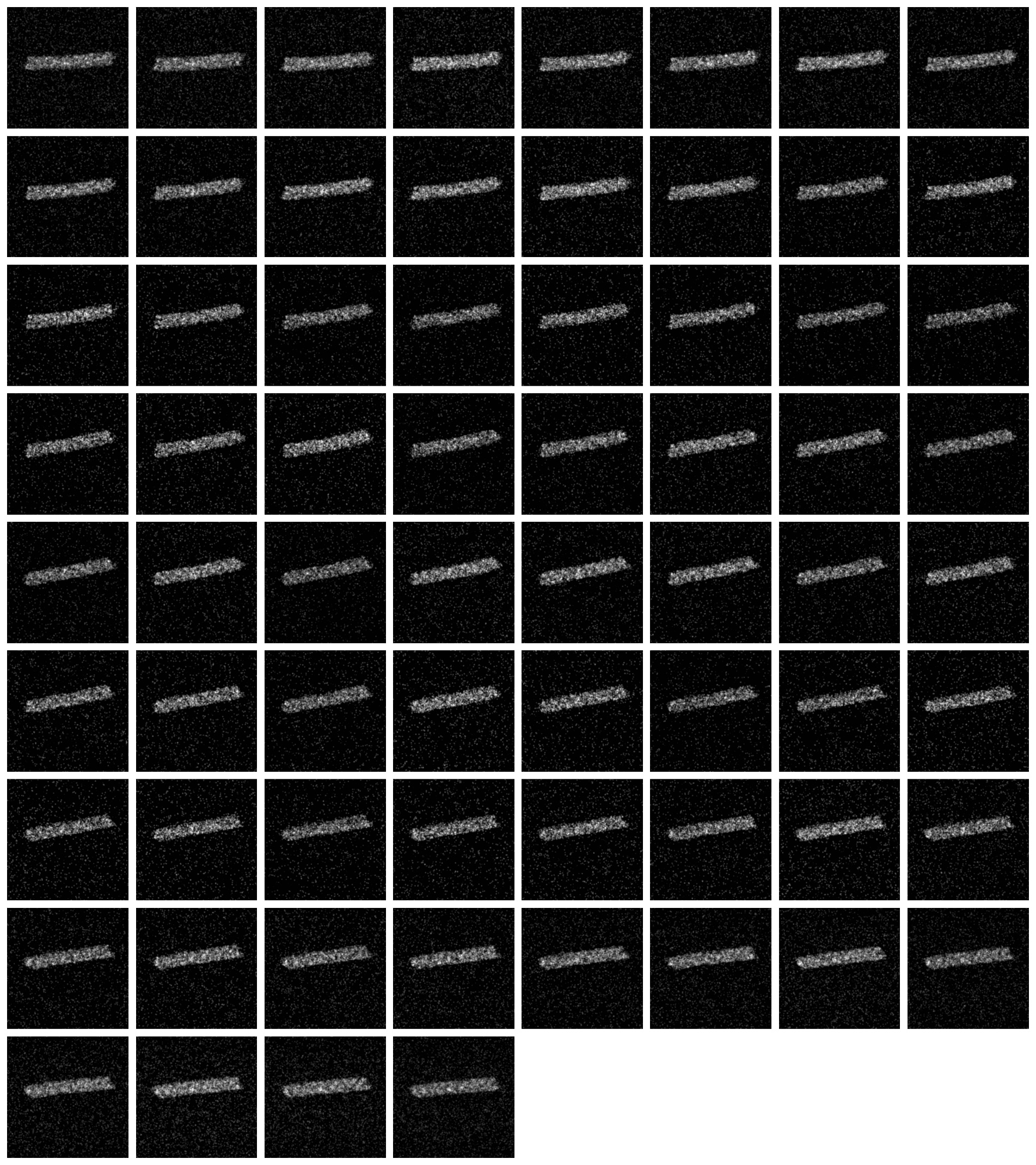


**Figure S24.** N element tomography images acquired from -70° to 64°.


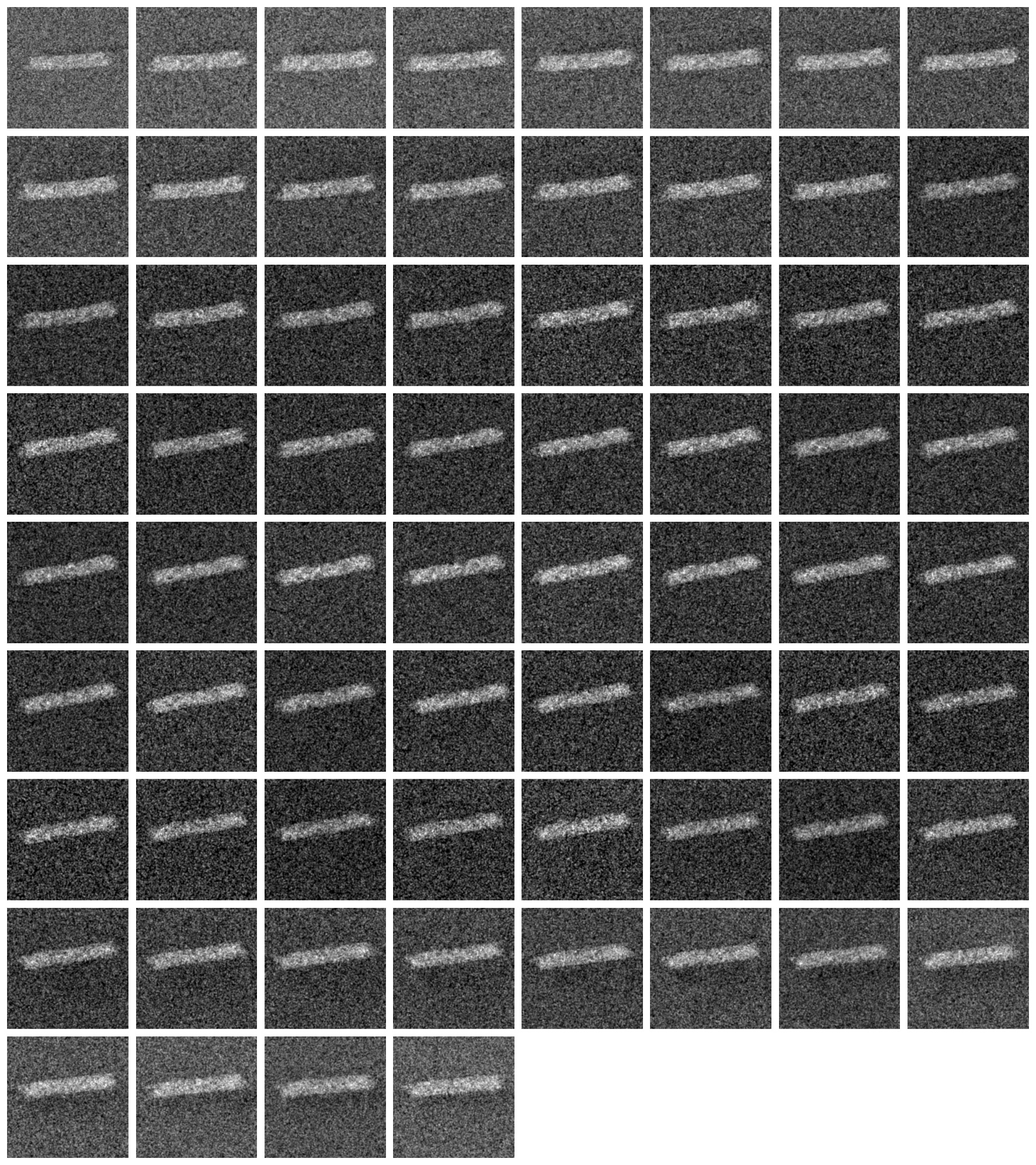


**Figure S25.** C element tomography images acquired from -70° to 64°.


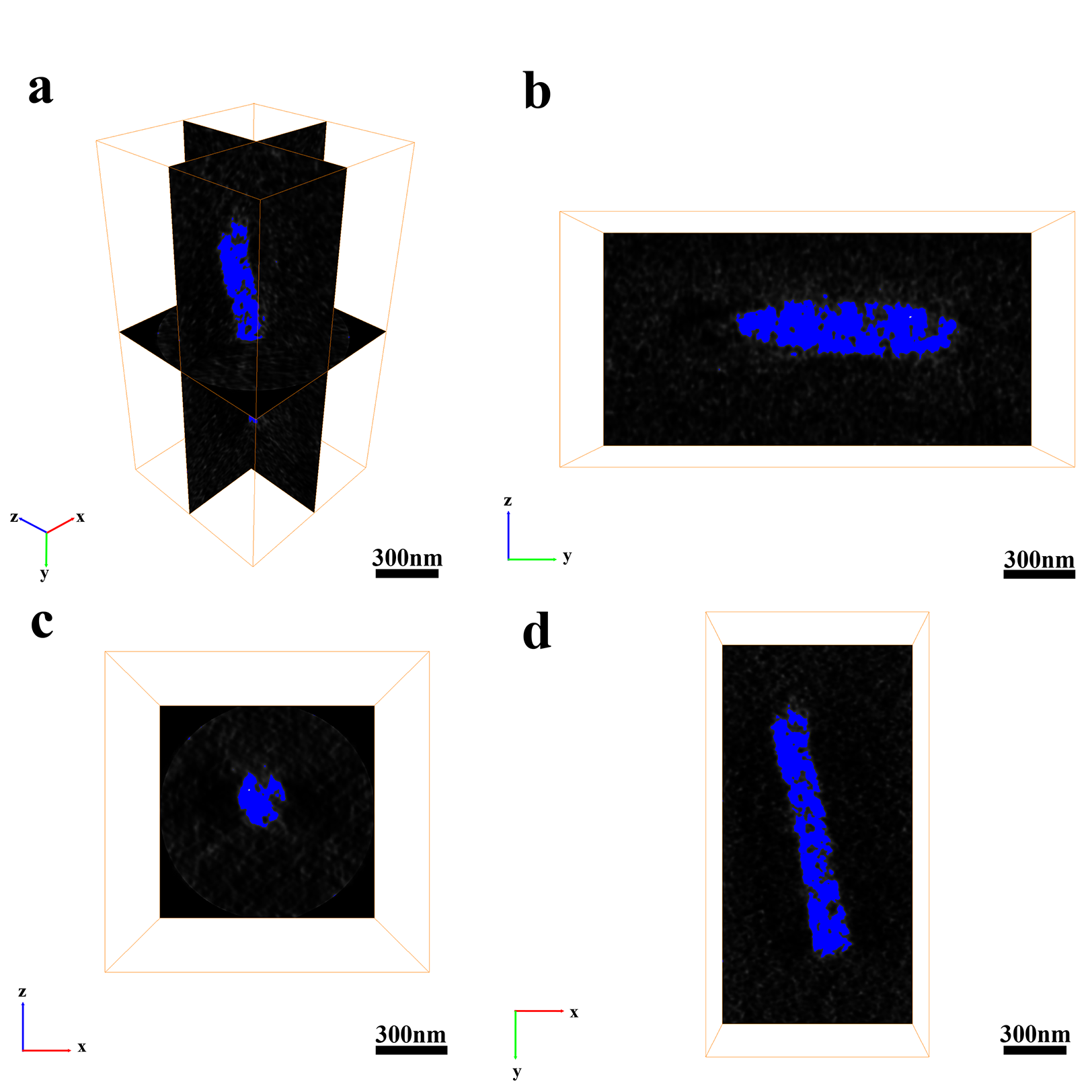


**Figure S26.** Ortho slice of 3D reconstruction in a b) side view, b) yz view, c) xz view, d) xy view.


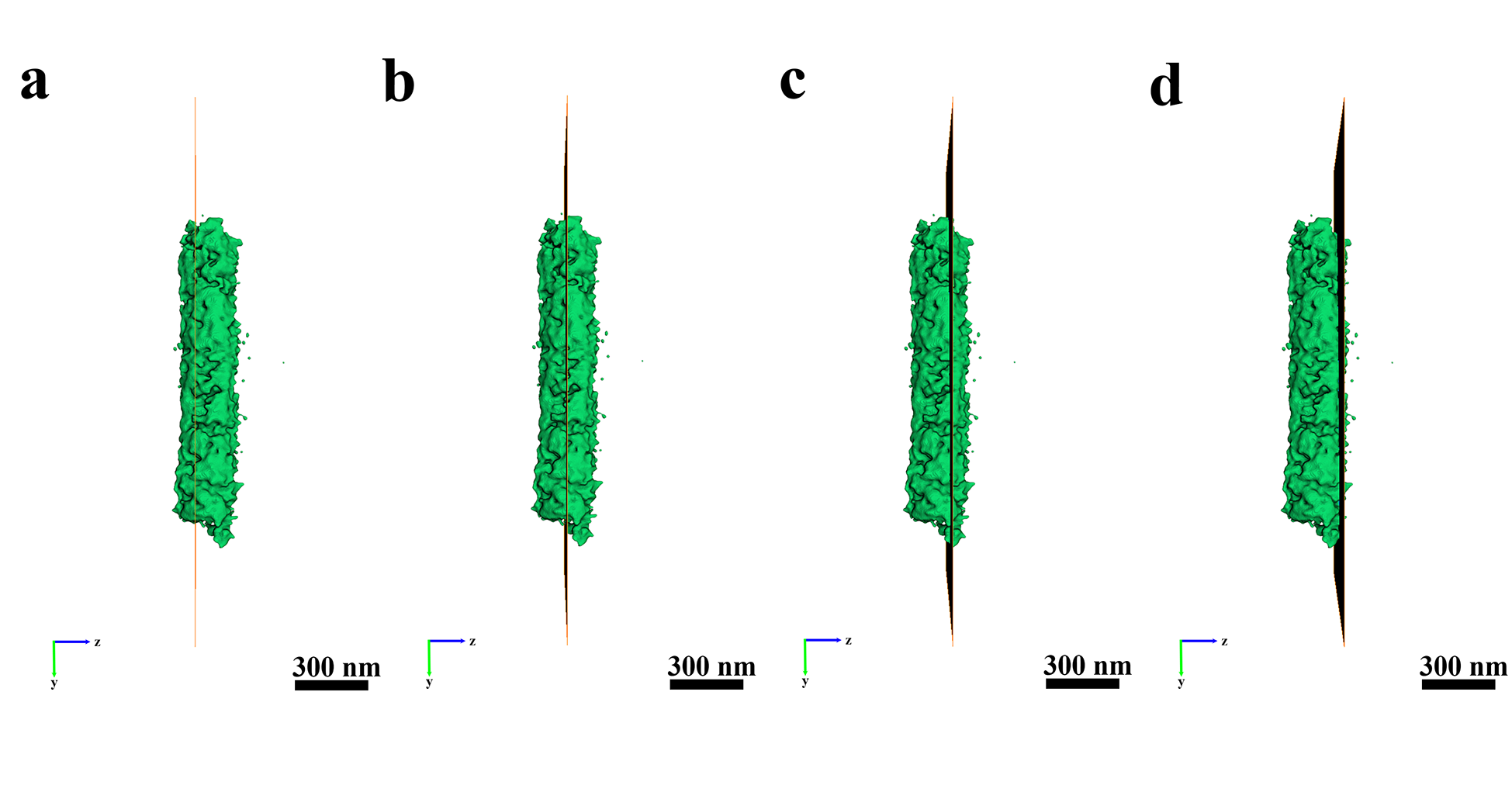


**Figure S27.** Slice images at No. a) 200, b) 220, c) 240, d) 260, and corresponding slice location.


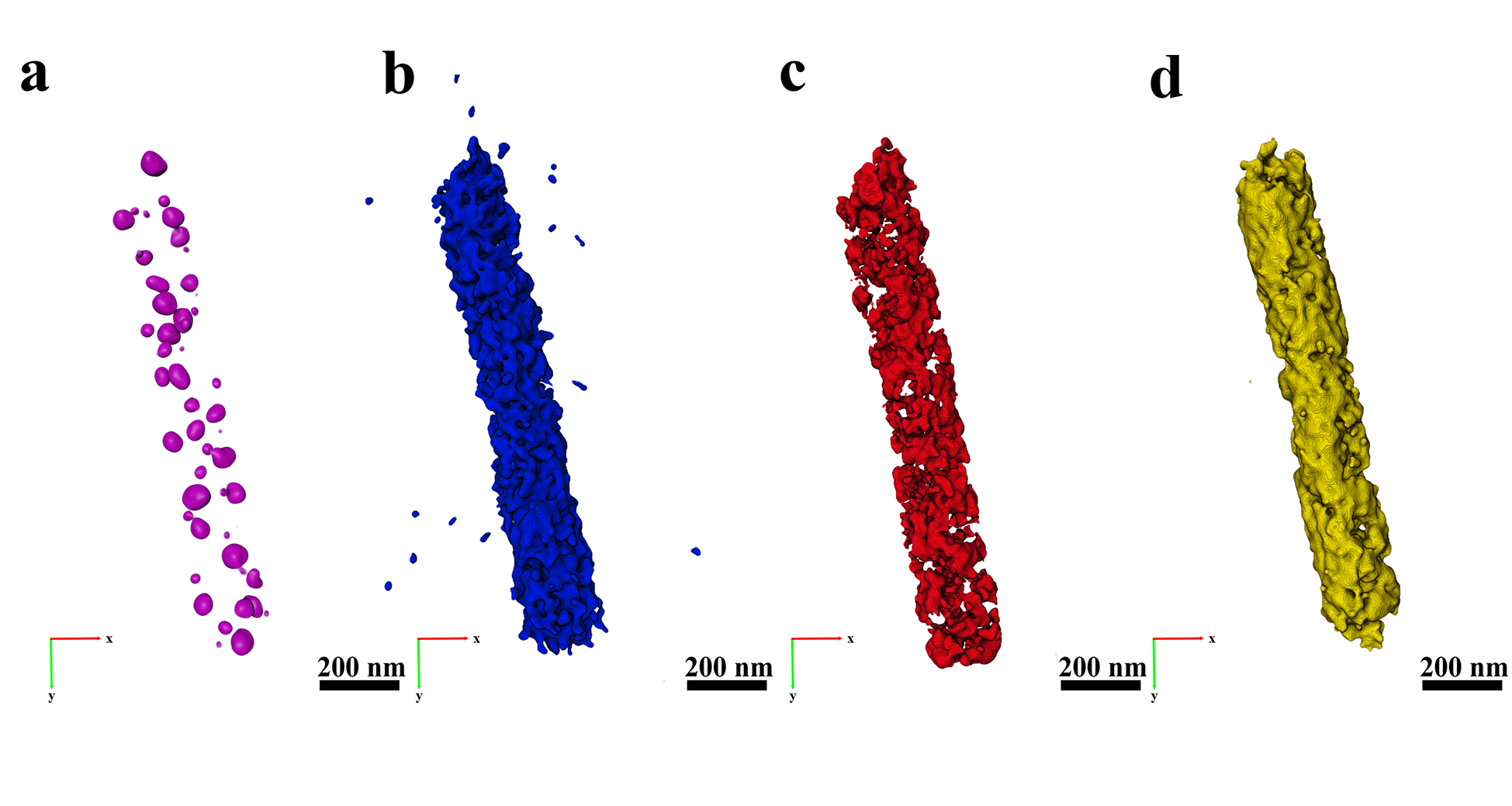


**Figure S28.** 3D reconstruction characterized element distribution of CoBNPCF-900, a) Co, b) B, c) N, and d) C elements.


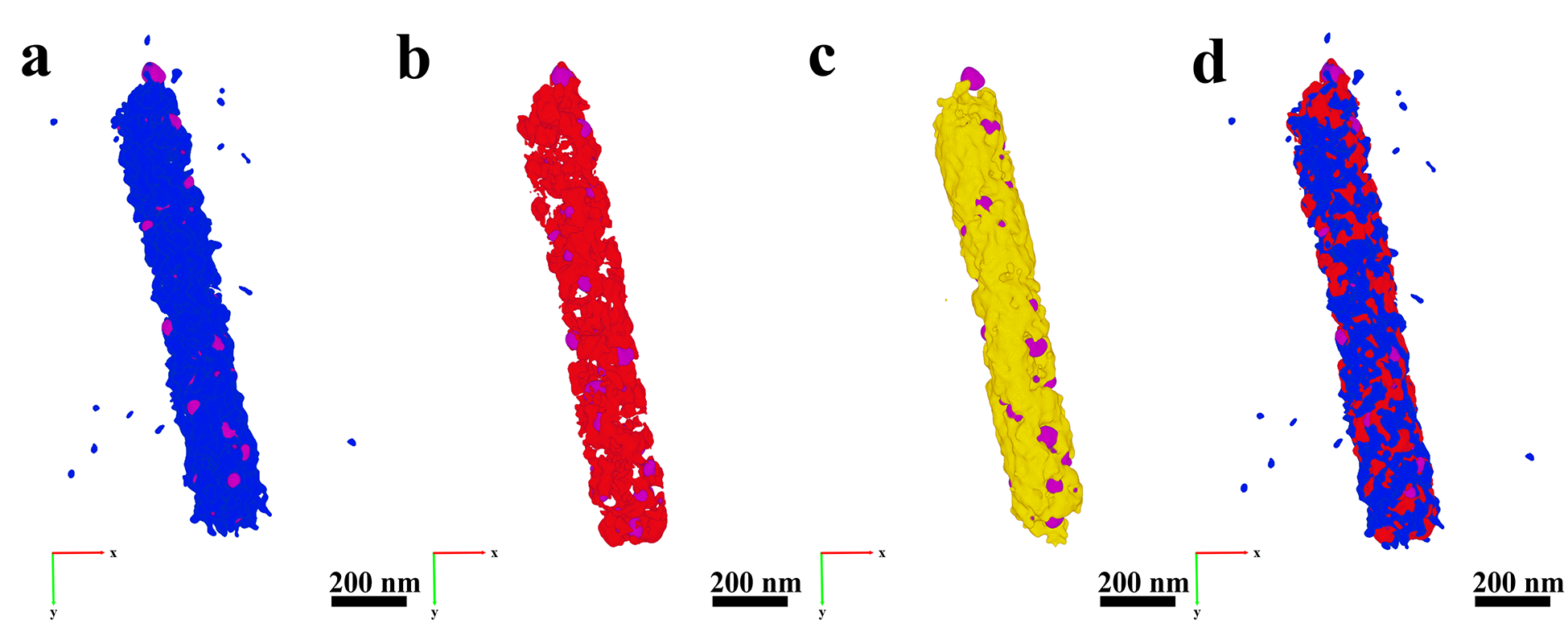


**Figure S29.** 3D reconstruction characterized elements distribution of Co@BNC core-shell structure, a) Co@B, b) Co@N, c) Co@C, d) Co@BN.


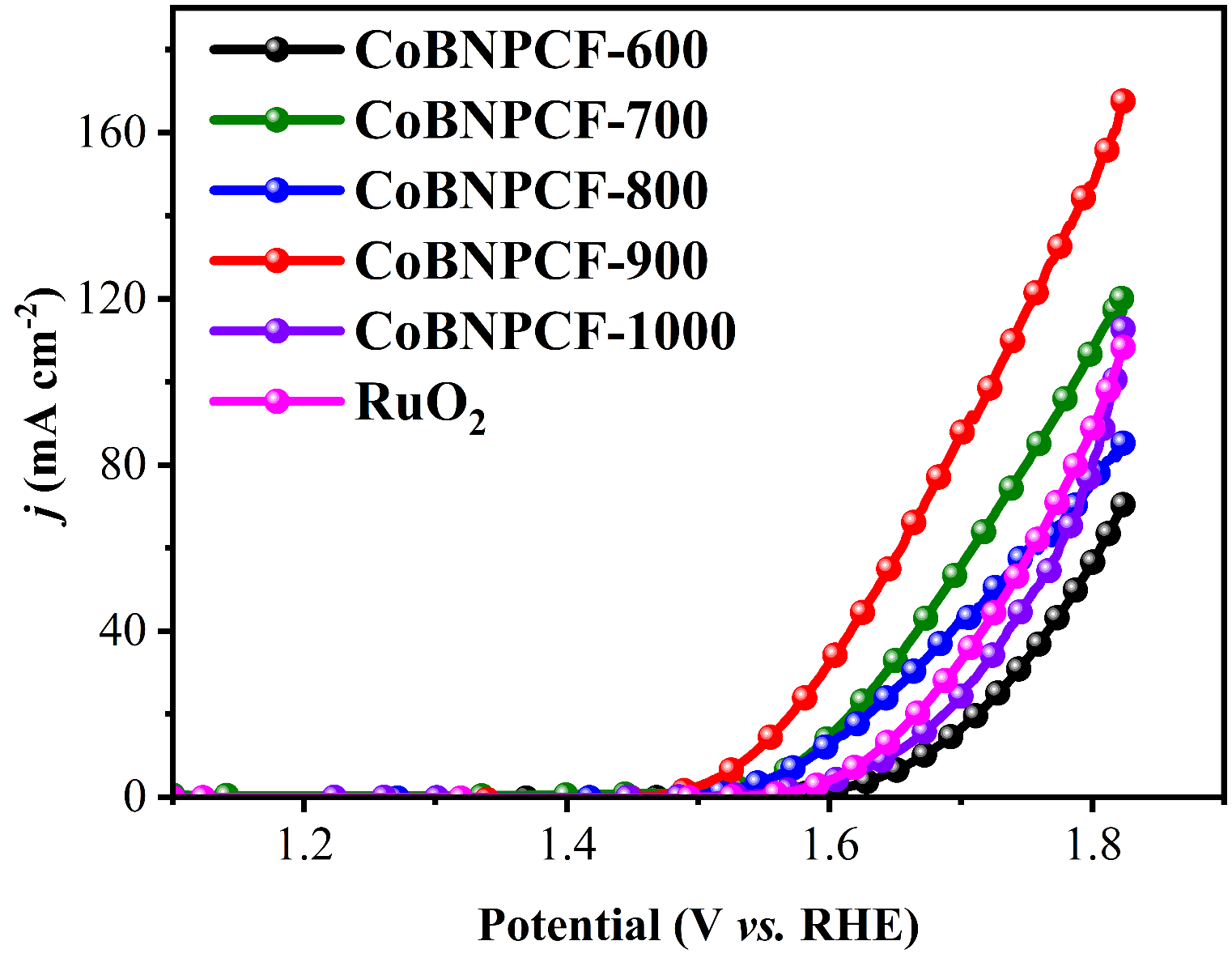


**Figure S30.** The LSV curves of OER without iR-correction.


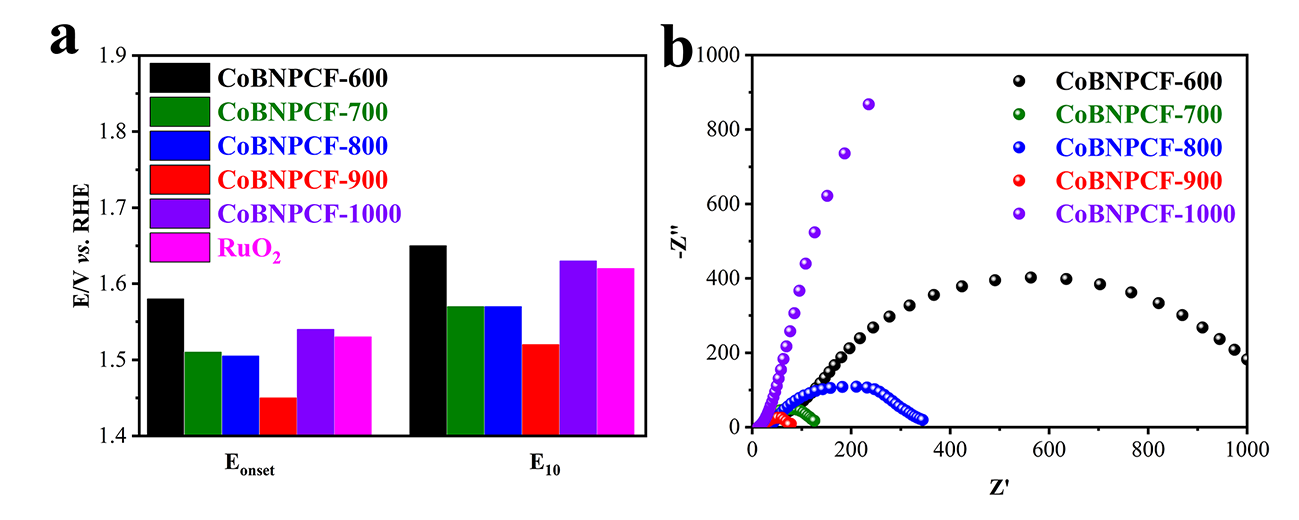


**Figure S31.** a) Histograms of E_onset_ and E_10_ values and b) EIS Nyquist plots.


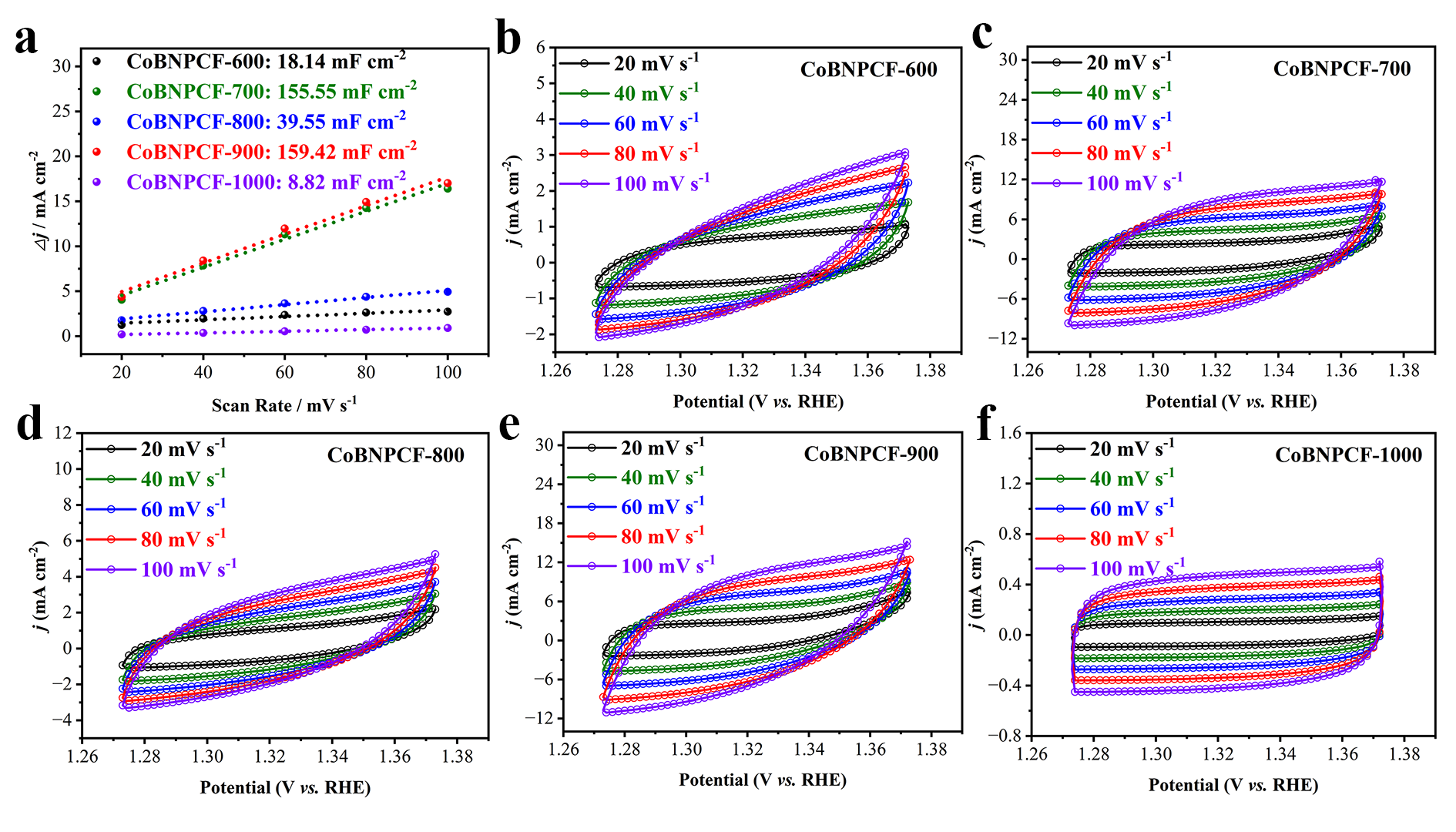


**Figure S32.** a) the capacitive currents at 1.32 V vs. RHE and CV curves of b) CoBNPCF-600, c) CoBNPCF-700, d) CoBNPCF-800, e) CoBNPCF-900, and f) CoBNPCF-1000.


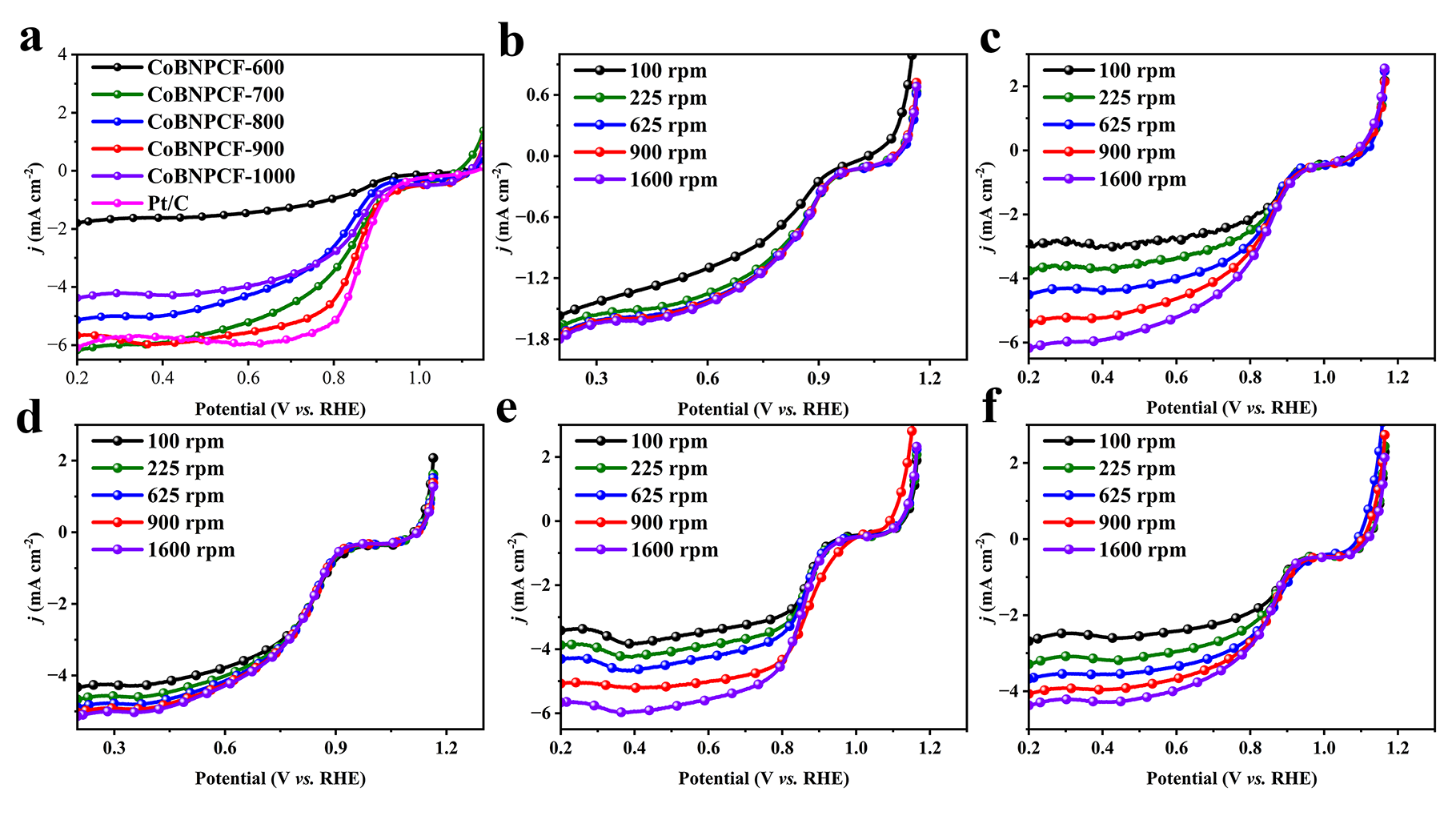


**Figure S33.** a) The LSV curves without iR-correction of CoBNPCFs-T. Different scan rates of LSV curves b) CoBNPCF-600, c) CoBNPCF-700, d) CoBNPCF-800, e) CoBNPCF-900, and f) CoBNPCF-1000.


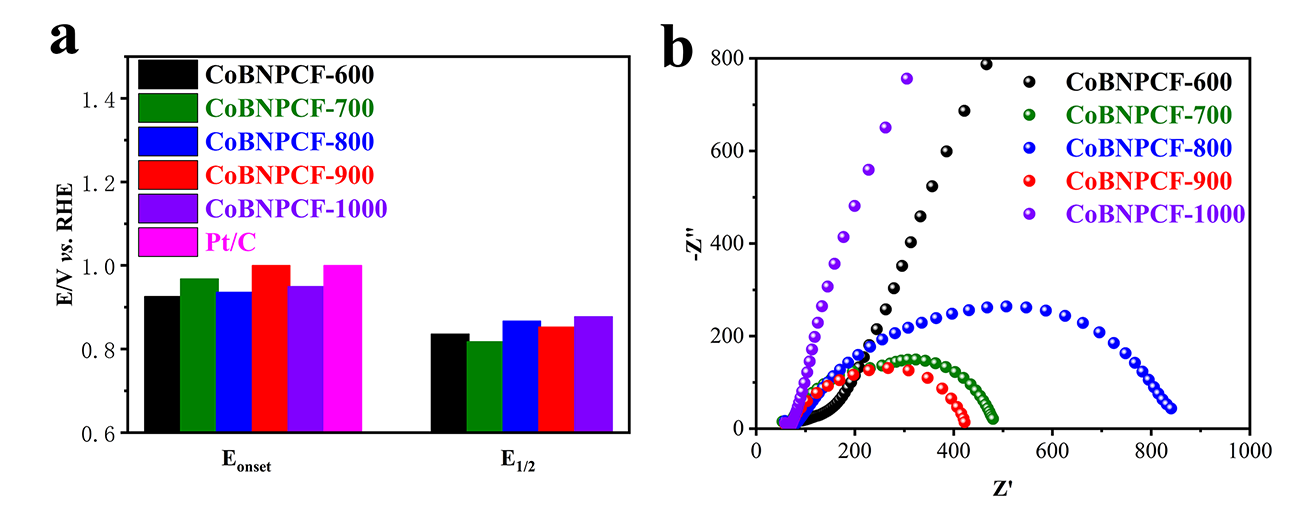


**Figure S34.** a) Histograms of E_onset_ and E_1/2_ values and b) EIS Nyquist plots.


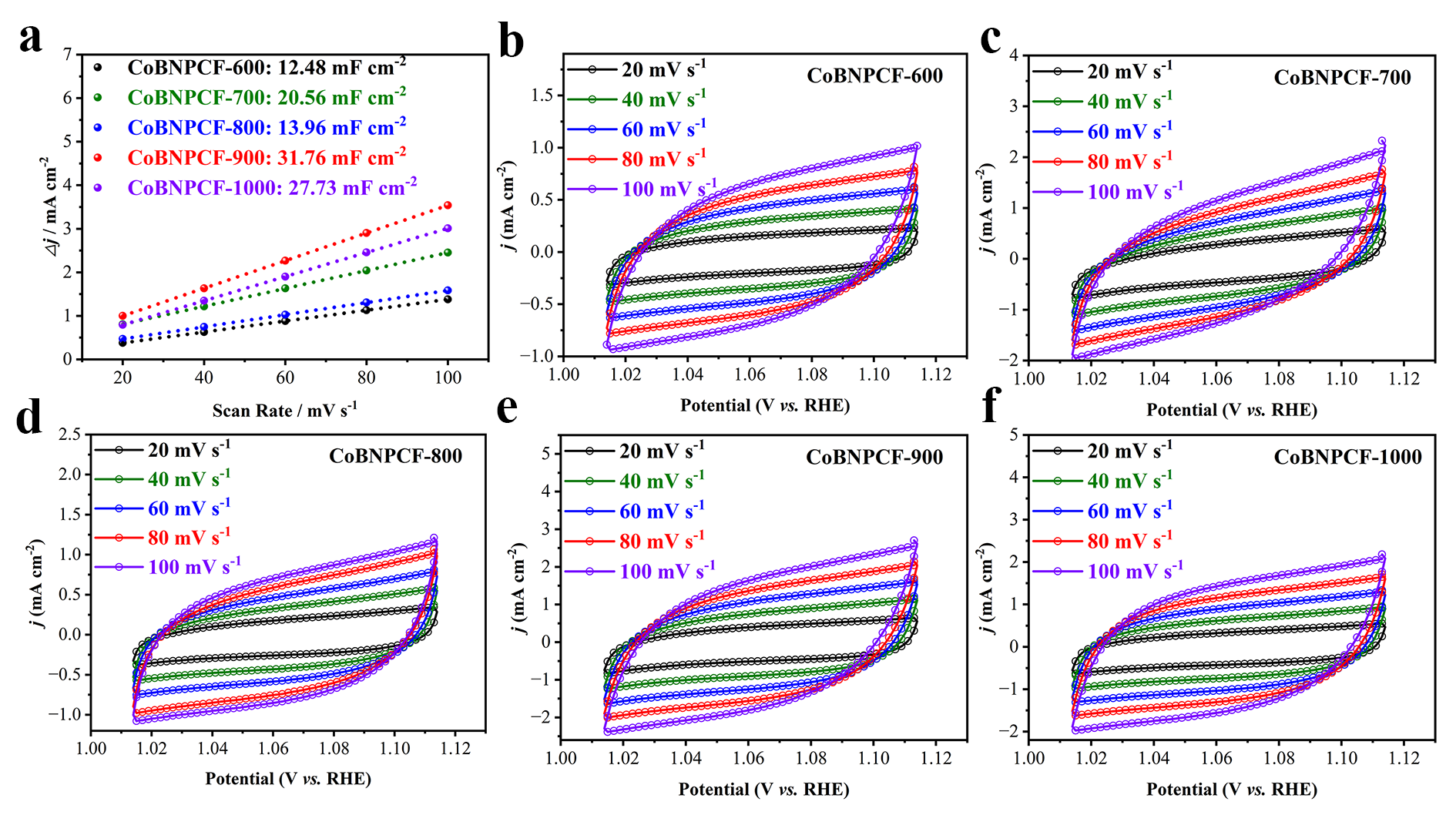


**Figure S35.** a) The capacitive currents at 1.06 V vs. RHE and CV curves of b) CoBNPCF-600, c) CoBNPCF-700, d) CoBNPCF-800, e) CoBNPCF-900, and f) CoBNPCF-1000.


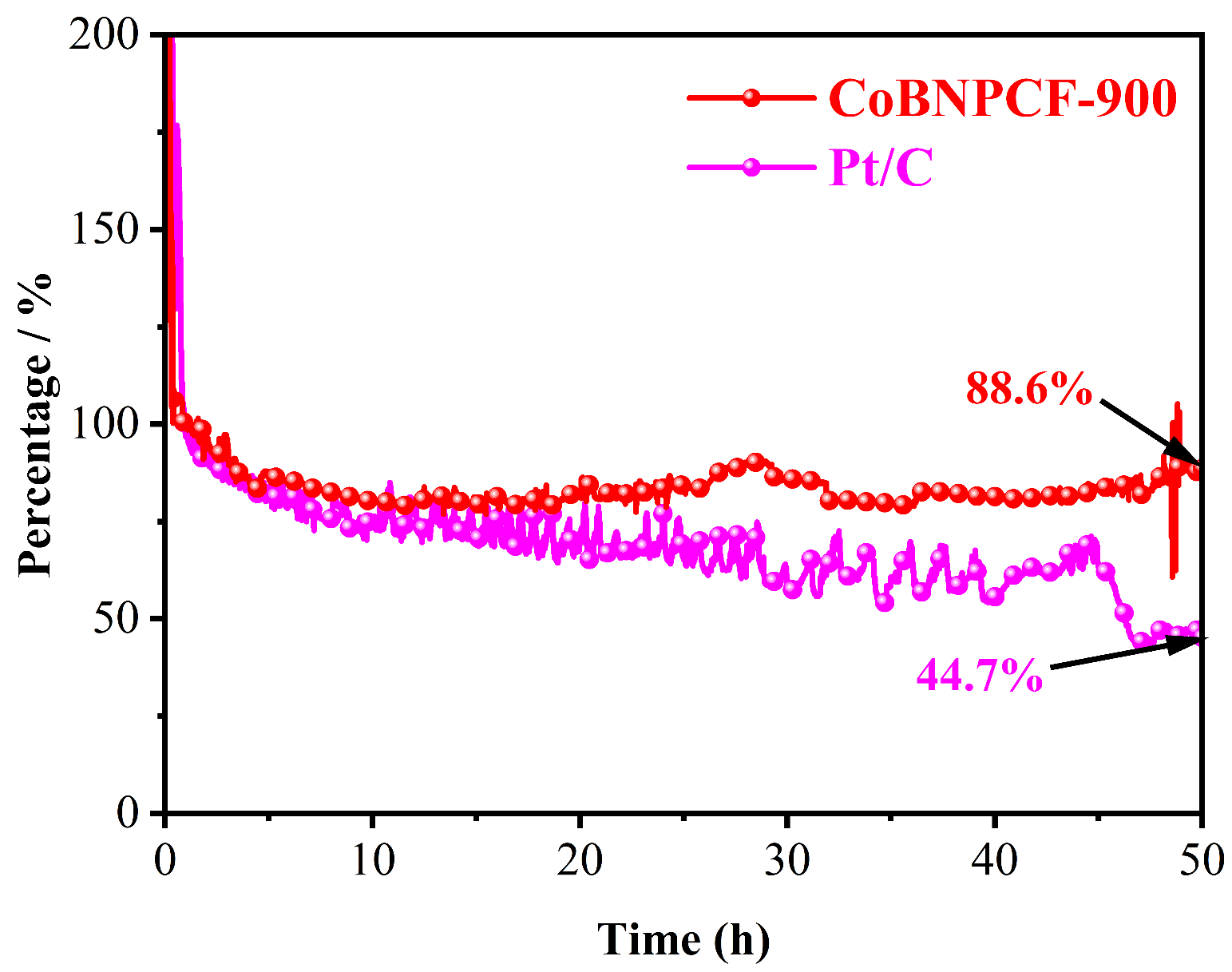


**Figure S36.** The catalytic I (percentages)-t curves recorded at + 0.94 V vs. RHE during ORR polarization for CoBNPCF-900 and Pt/C.

**Table S3.** The electrochemical properties of electrocatalysts recently reported.

| Catalyst | Electrolyte | E_1/2_ (V) | E_10_ (V) | △E | Ref. |
| --- | --- | --- | --- | --- | --- |
| CoBNPCF-800 | 0.1 M KOH: ORR  1 M KOH: OER | 0.86 | 1.52 | 0.66 | This work |
| Arc-Co SAC | 0.1 M KOH: ORR  1 M KOH: OER | 0.86 | 1.58 | 0.73 | ^[6]^ |
| Cu-Co/NC | 0.1 M KOH | 0.92 | 1.56 | 0.64 | ^[7]^ |
| Co_4_N@CoON/PCGN | 0.1 M KOH | 0.855 | 1.595 | 0.74 | ^[8]^ |
| Co_1_/BNG | 0.1 M KOH: ORR  1 M KOH: OER | 0.867 | 1.595 | 0.728 | ^[9]^ |
| CoN-BC-0.3 | 0.1 M KOH: ORR  1 M KOH: OER | 0.83 | 1.55 | 0.72 | ^[10]^ |
| CoN NLF | 0.1 M KOH | 0.863 | 1.53 | 0.668 | ^[11]^ |
| Fe_3_Co_1_–NC | 0.1 M KOH: ORR  1 M KOH: OER | 0.87 | 1.62 | 0.75 | ^[12]^ |
| IO─Ni_x_Co_9-x_S_8_@NSC | 0.1 M KOH | 0.926 | 1.519 | 0.59 | ^[13]^ |
| PPcFeCo/3D-G | 0.1 M KOH: ORR  1 M KOH: OER | 0.89 | 1.58 | 0.7 | ^[14]^ |


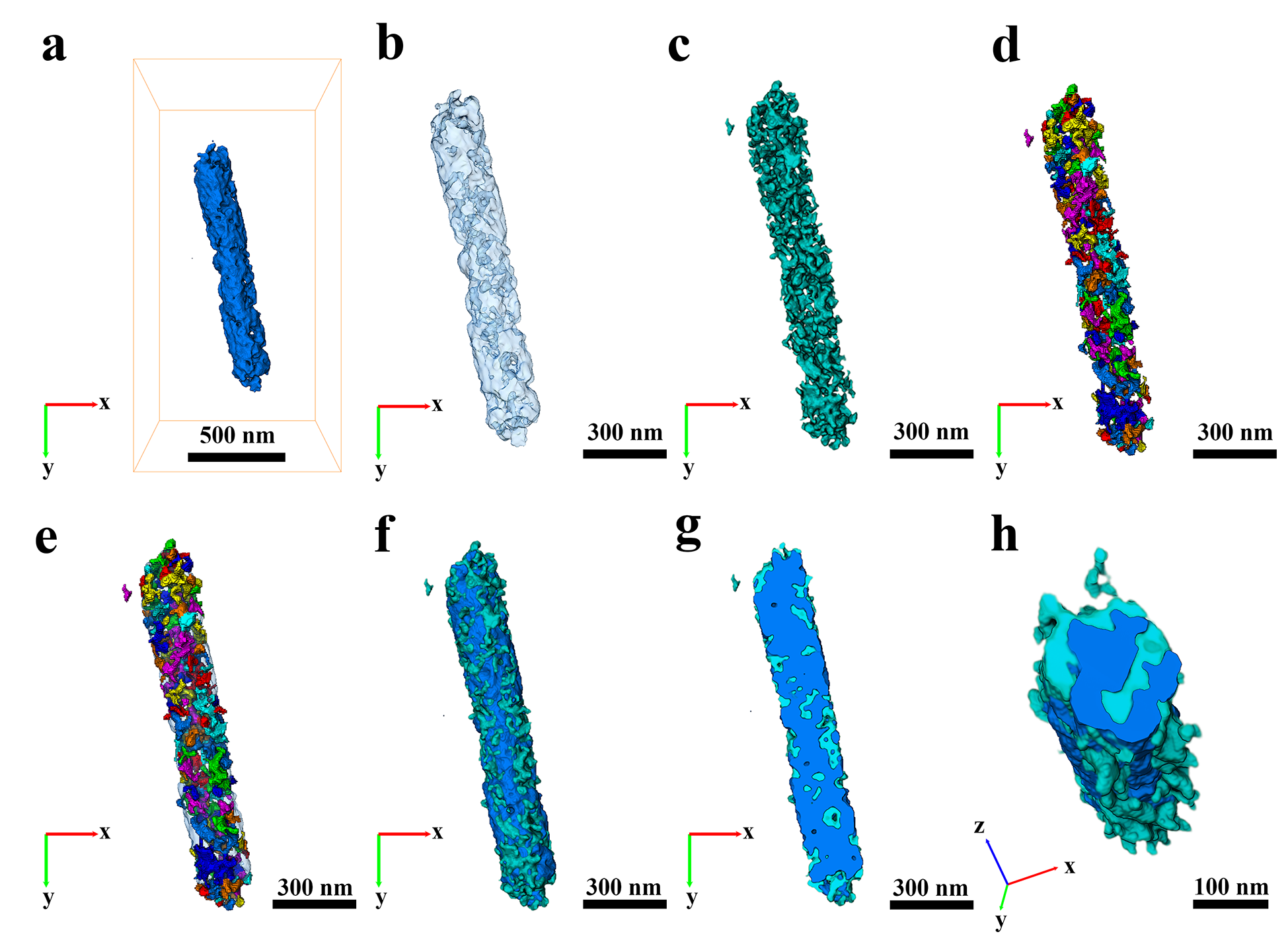


**Figure S37.** a) 3D reconstructed carbon matrix. b) Generated surface of carbon matrix. c) Pores distribution of carbon matrix. d) Pores in a unit view. e) Pores distribution in a transparency surface. f-h) Segmentation of pores and matrix.


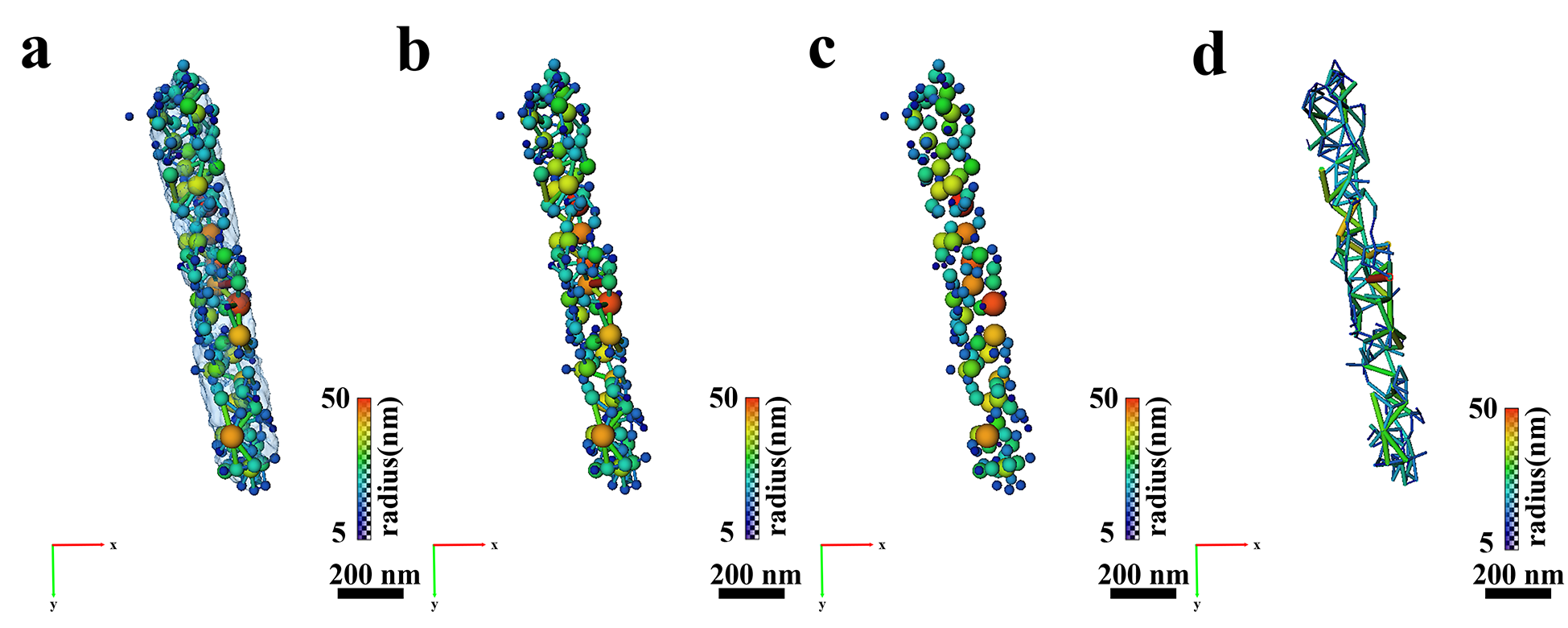


**Figure S38.** a) Balls-stick models performed pores and throat distribution with surface. b) Pores and throat distribution. c) Pores. d) Throats.


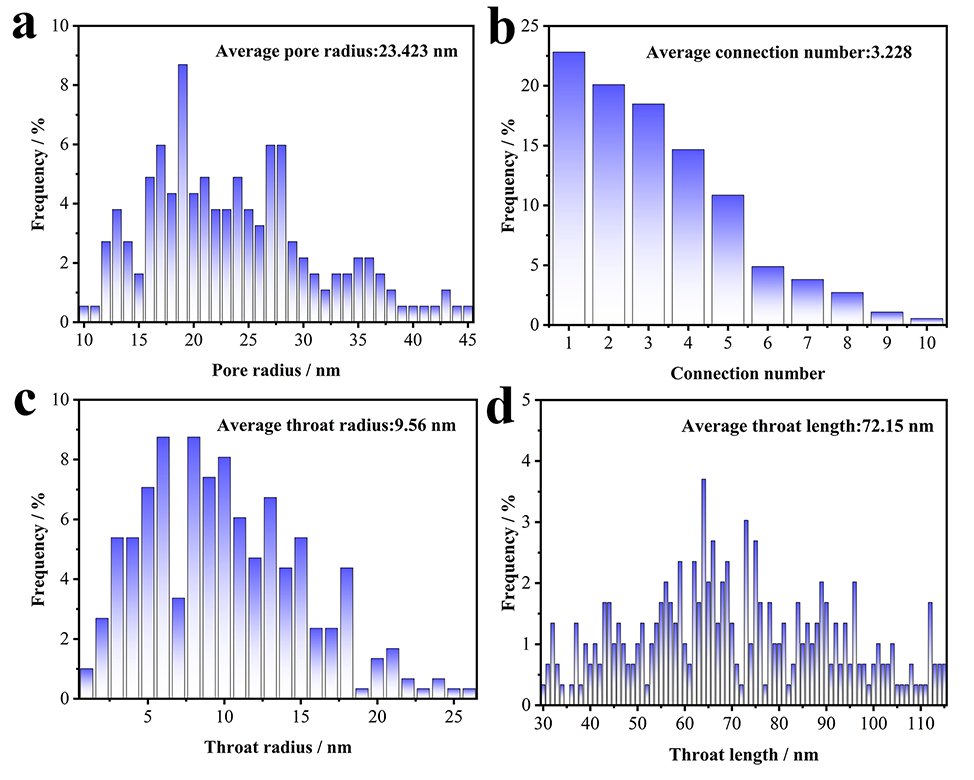


**Figure S39.** a) pores’ size b) pores’coordination number c) throats’ size and d)throat length of carbon matrix.


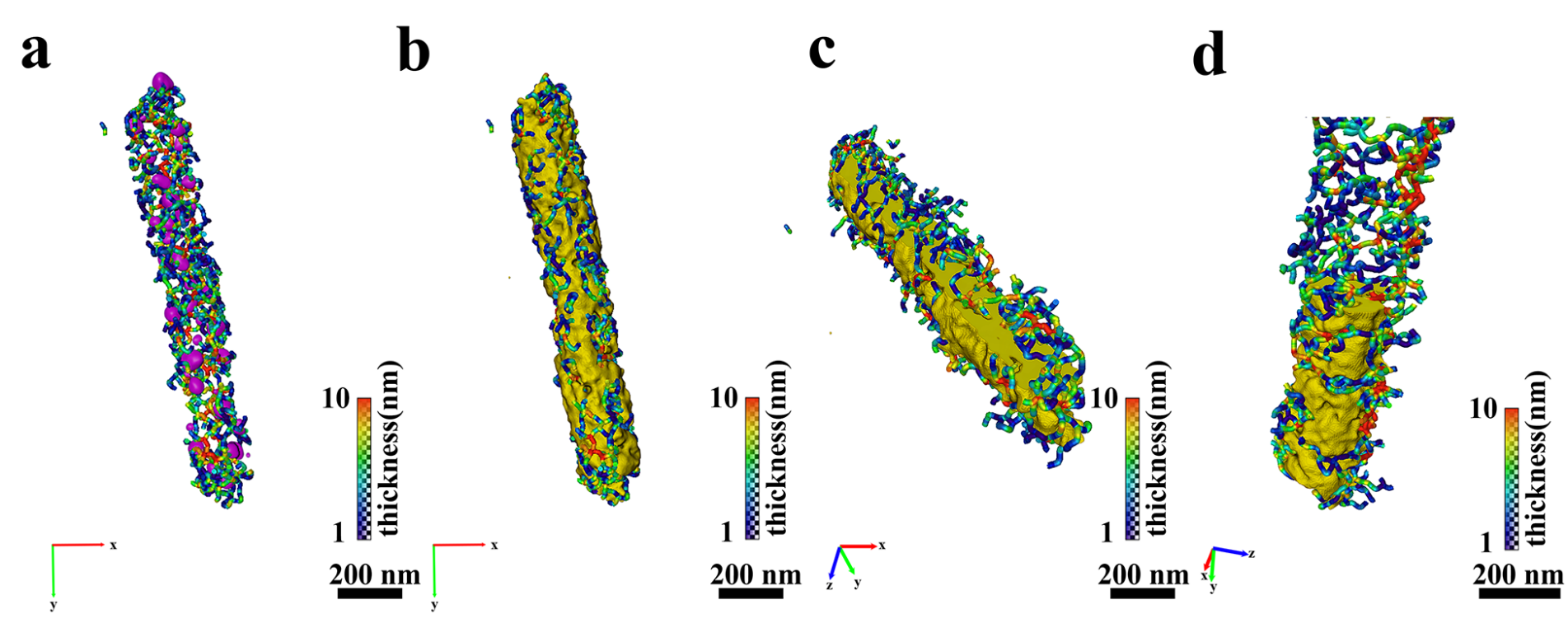


**Figure S40.** a) Location of Co nanoparticles and channels. b-d) Segmentation of channels and matrix


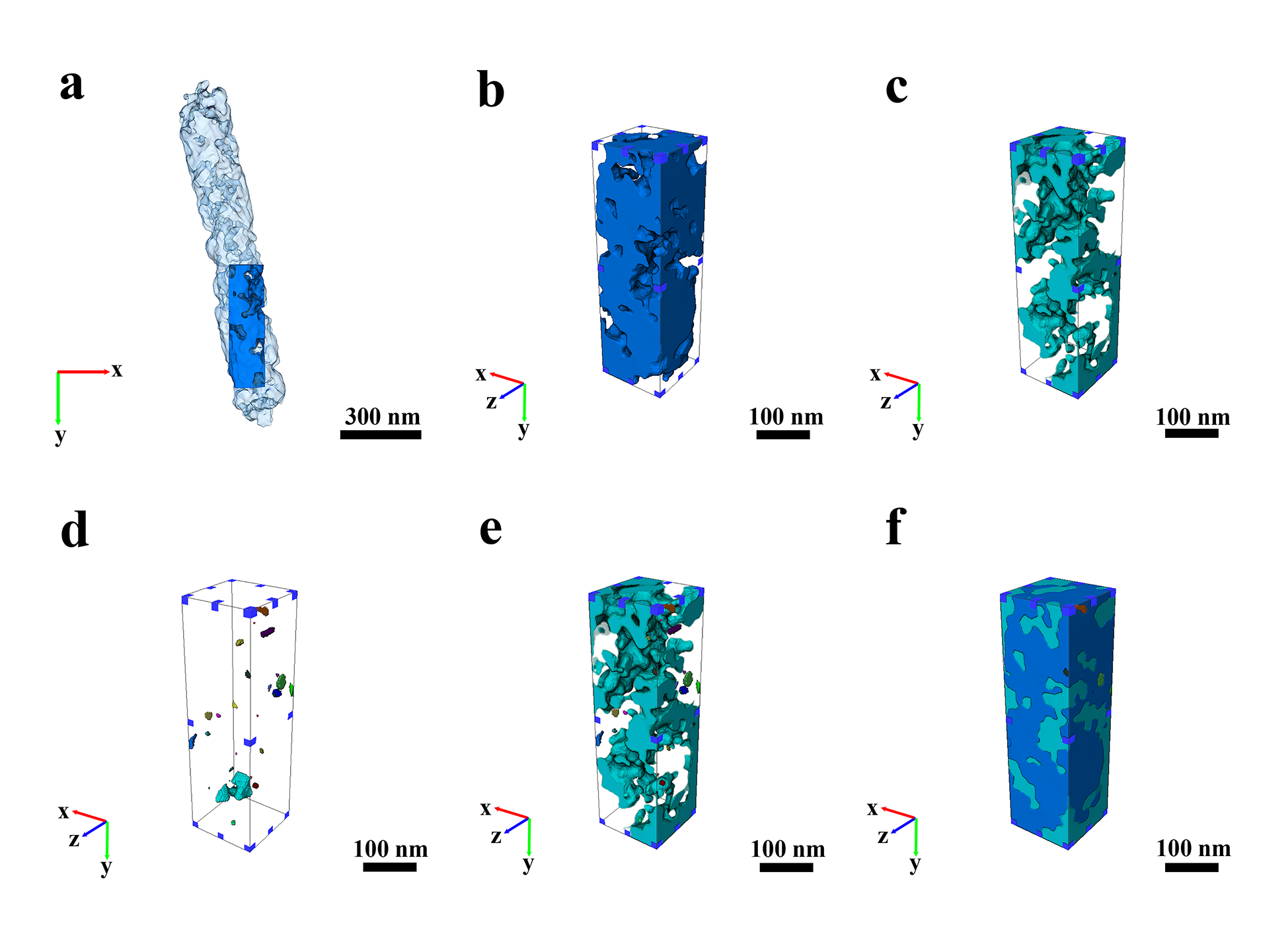


**Figure S41.** a) Location of extraction sub-volume. b) Extracted carbon matrix. c) Connected pores. d) Isolated pores. e) Both connected and isolated pores. f) Segmentation of sub-volume and pores.


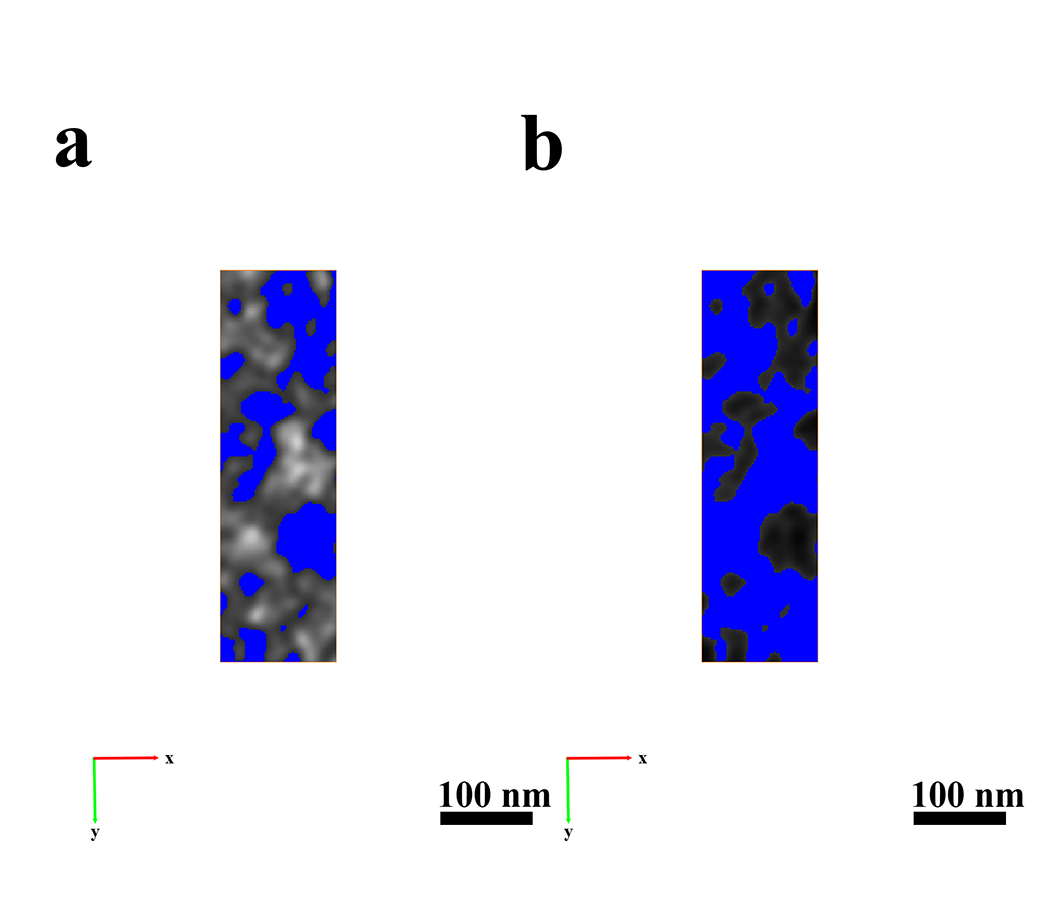


**Figure S42.** a) Ortho slice of pores segmentation. b) Ortho slice of carbon matrix segmentation.


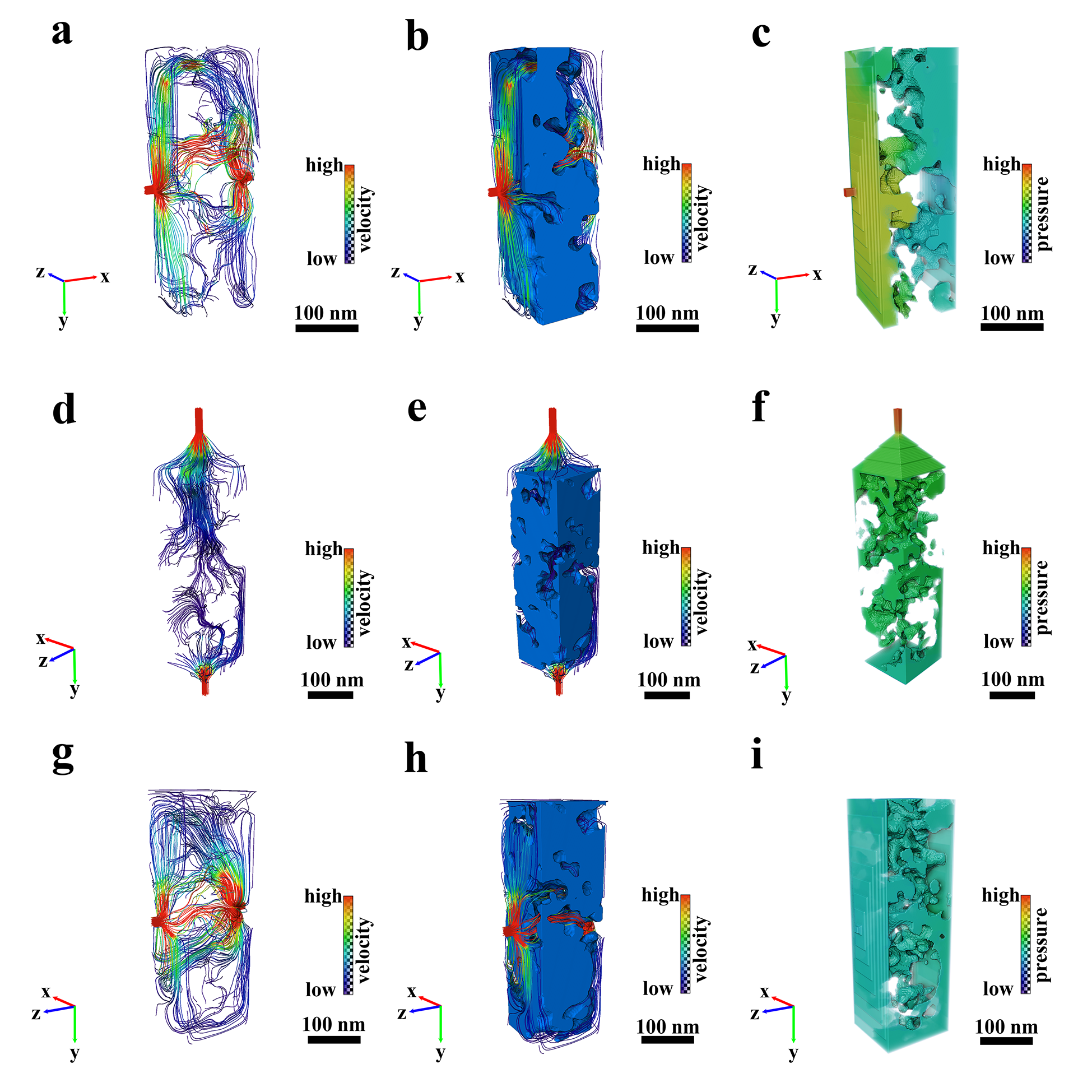


**Figure S43.** a) Streamlines b) carbon matrix and streamlines and c) corresponding fluid pressure from x direction. d) Streamlines e) carbon matrix and streamlines and cf) corresponding fluid pressure from y direction. g) Streamlines h) carbon matrix and streamlines and i) corresponding fluid pressure from z direction.


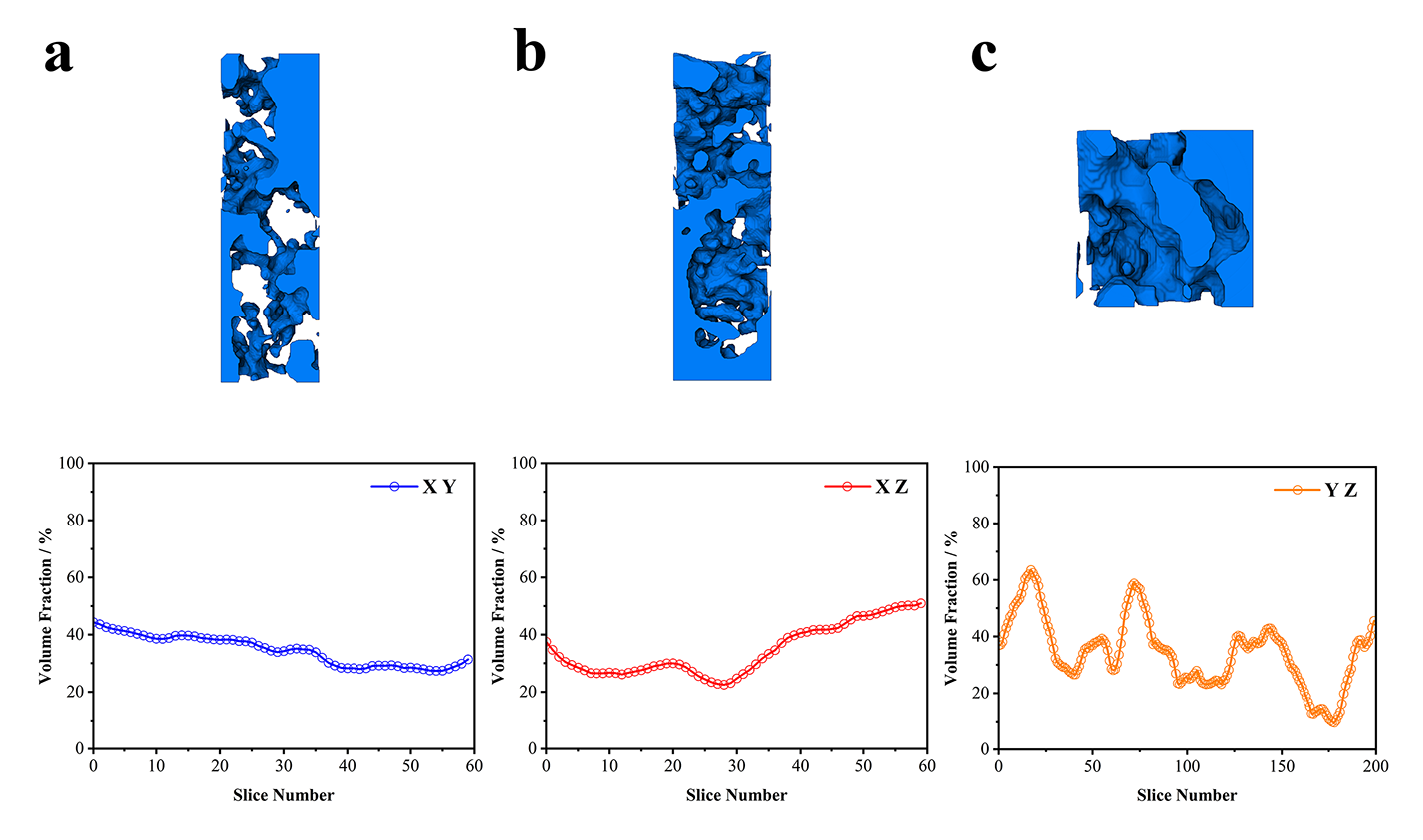


**Figure S44.** Volume fraction of a) xy plane, b) xz plane, and a) yz plane.


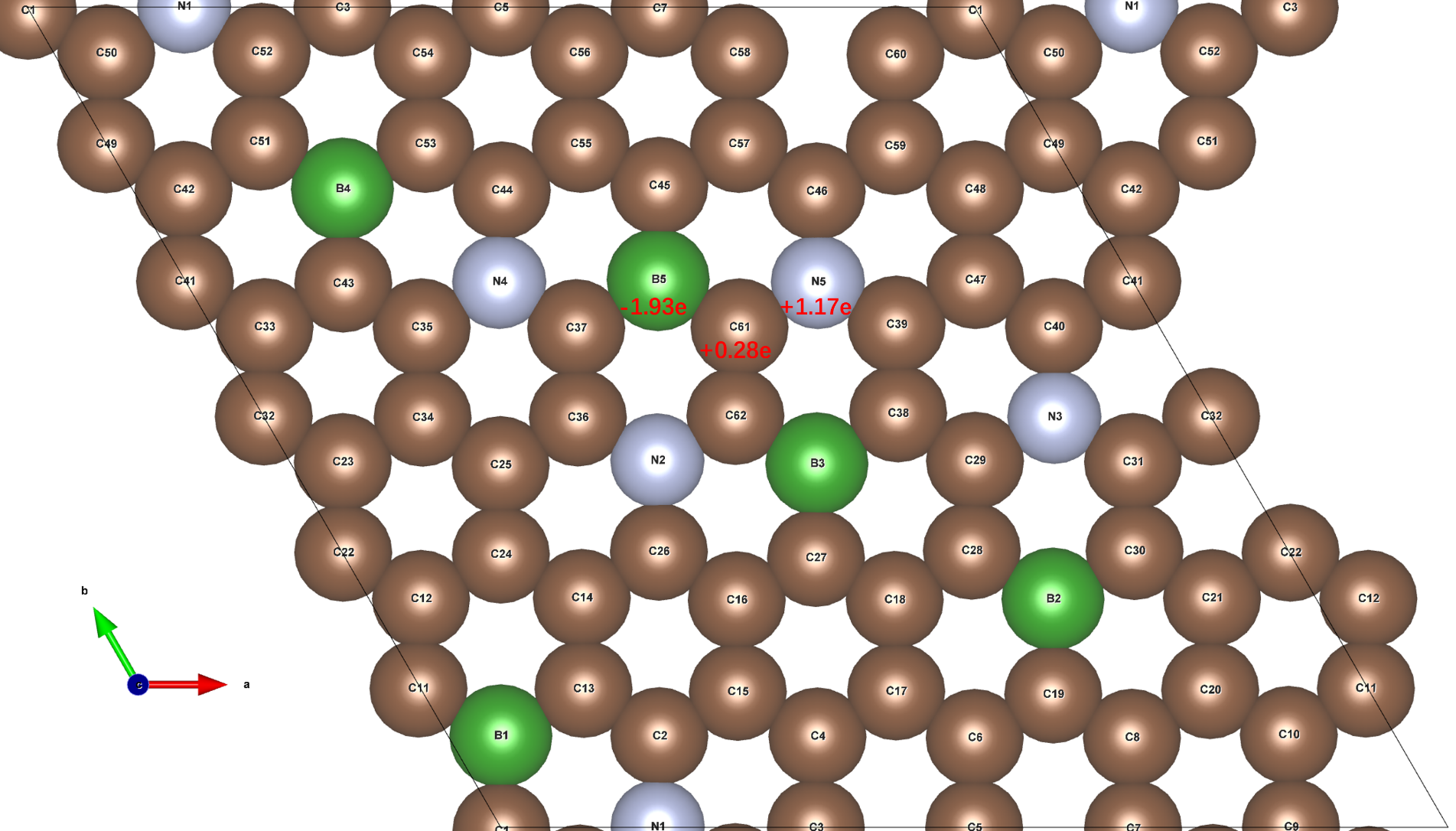


**Figure S45.** Bader charge analysis of BNC.


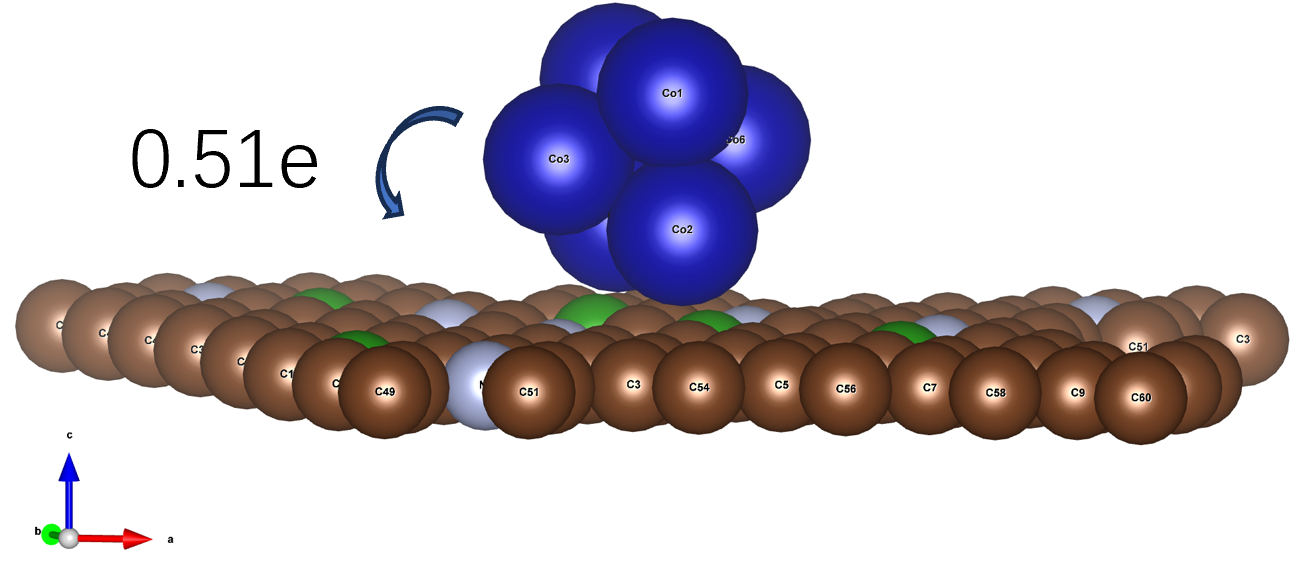


**Figure S46.** Interface Bader charge analysis of CoBNC.


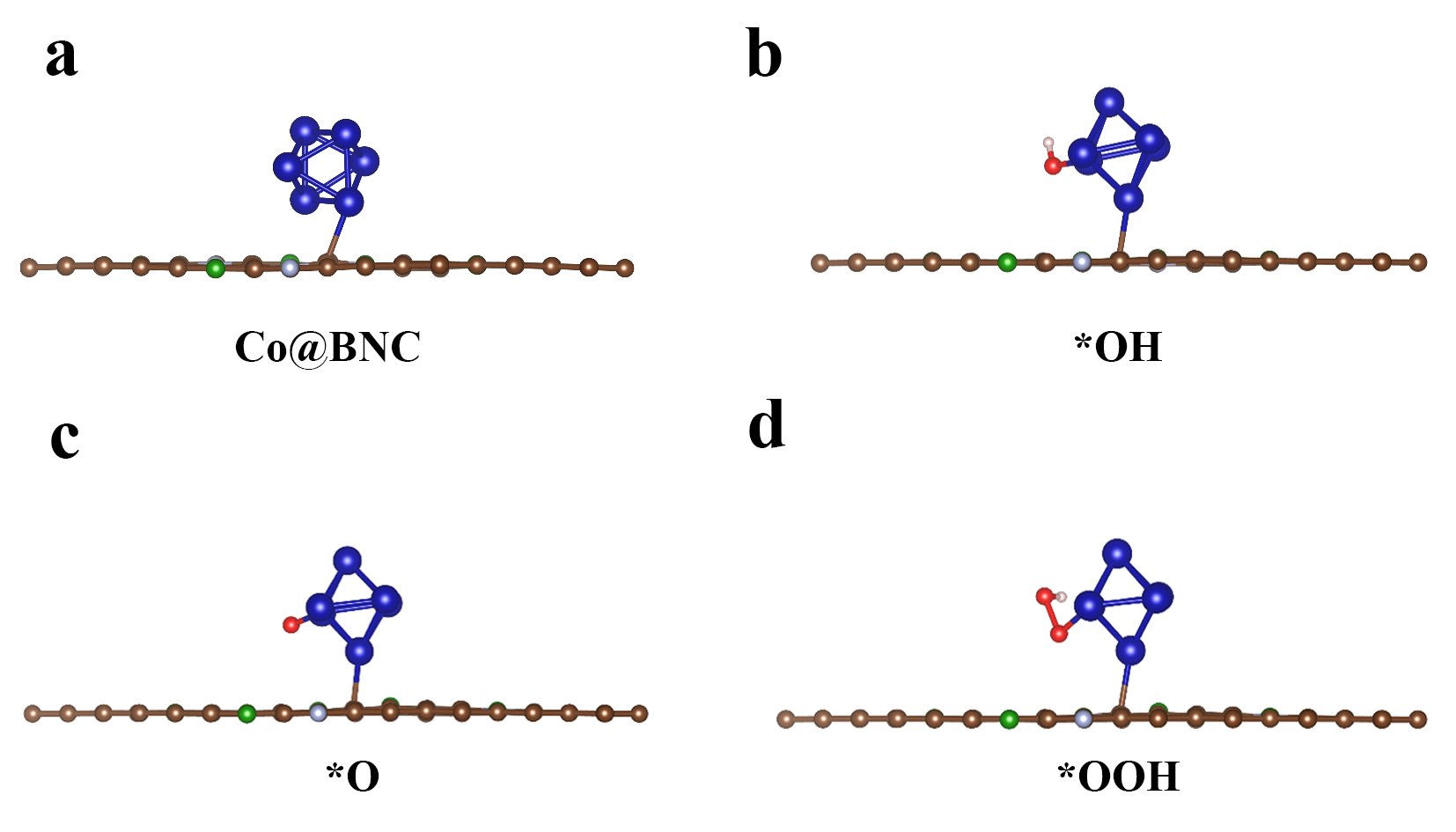


**Figure S47.** Configurations of adsorbates for OER process in Co@BNC model. Brown, green, gray, blue, red, and white sphere represent C, B, N, Co, O, and H atoms, respectively.


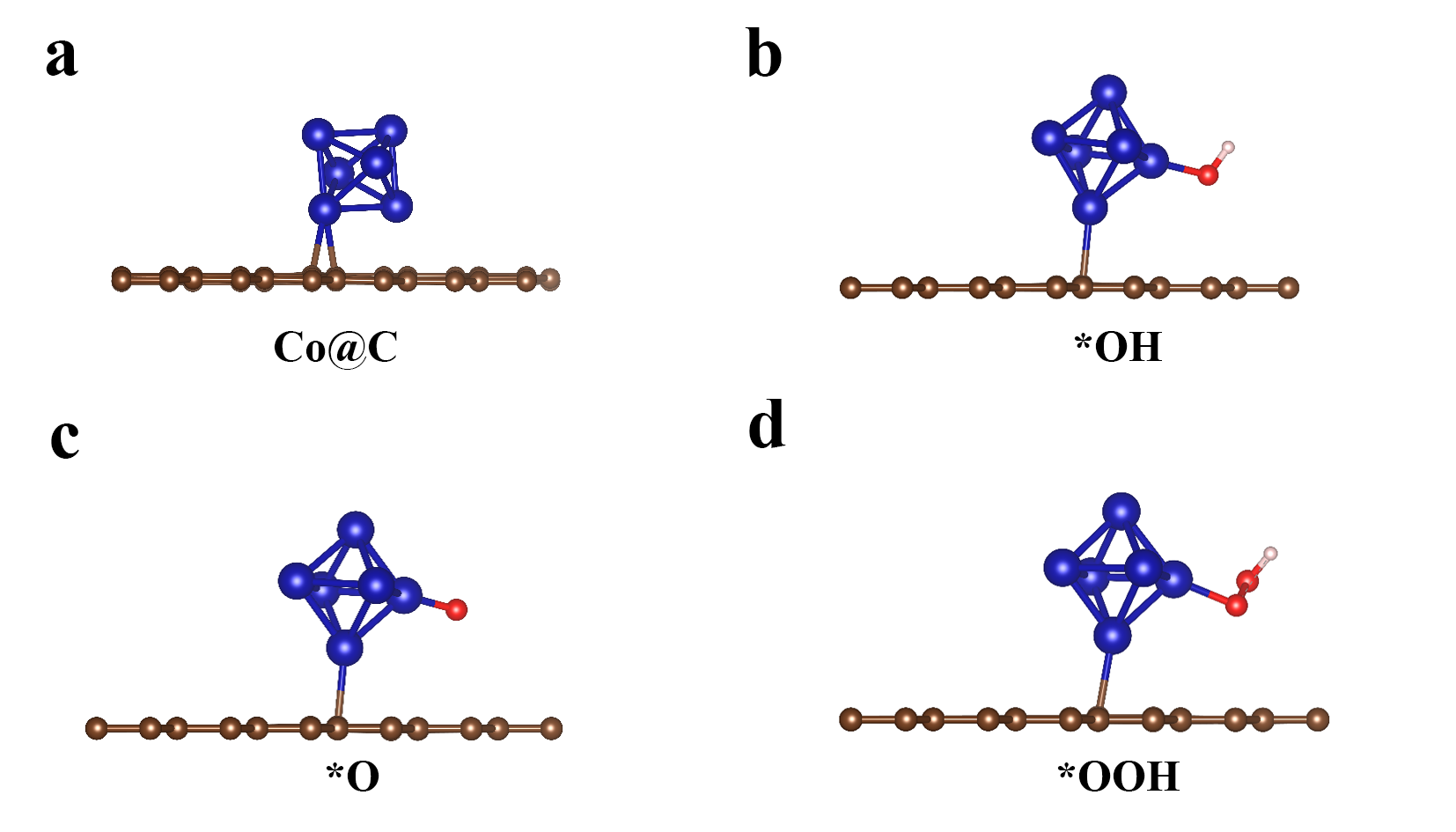


**Figure S48.** Configurations of adsorbates for OER process in Co@C model. Brown, blue, red, and white sphere represent C, Co, O, and H atoms, respectively.


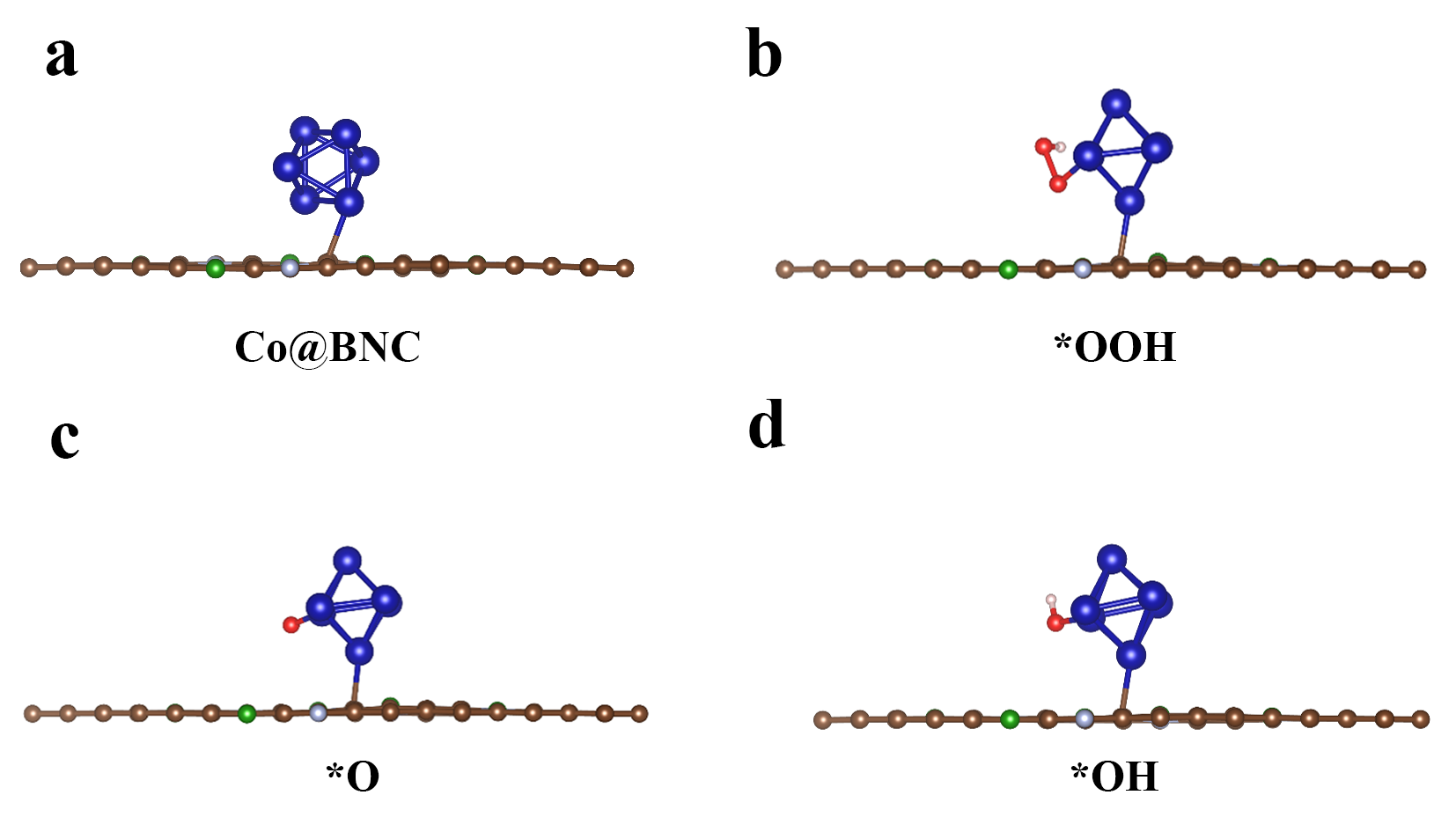


**Figure S49.** Configurations of adsorbates for ORR process in Co@BNC model. Brown, green, gray, blue, red, and white sphere represent C, B, N, Co, O, and H atoms, respectively.


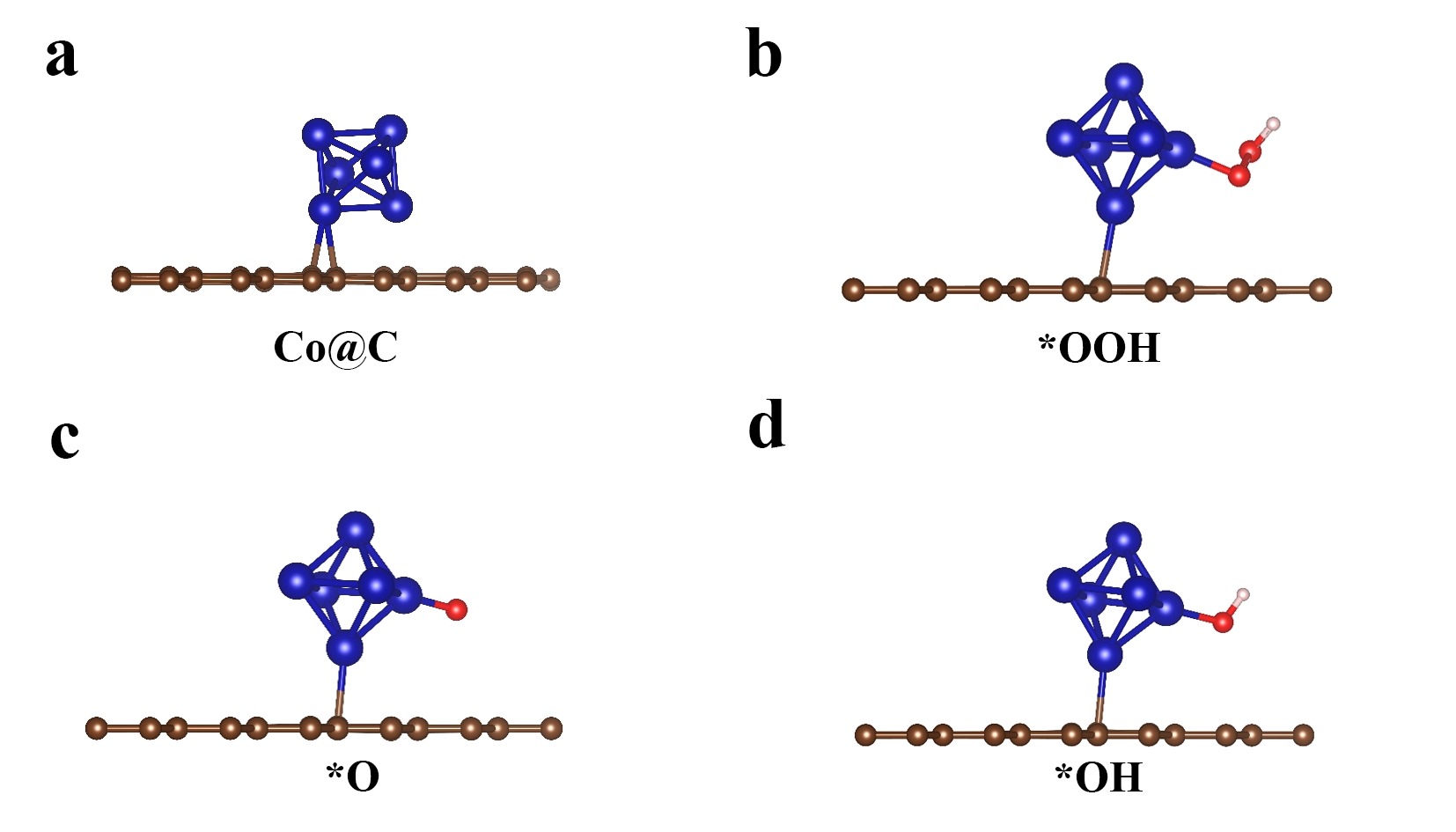


**Figure S50.** Configurations of adsorbates for ORR process in Co@C model. Brown, blue, red, and white sphere represent C, Co, O, and H atoms, respectively.

**Table S4.** The calculated △G values of OER for Co@BNC and Co@C models at U=0 V.

| Models | △G_1_ (eV) | △G_2_ (eV) | △G_3_ (eV) | △G_4_ (eV) |
| --- | --- | --- | --- | --- |
| Co@BNC | -0.01 | 0.86 | 2.26 | 1.81 |
| Co@C | -0.32 | 0.84 | 2.36 | 2.04 |

**Table S5.** The calculated △G values of OER for Co@BNC and Co@C models at U=1.23 V.

| Models | △G_1_ (eV) | △G_2_ (eV) | △G_3_ (eV) | △G_4_ (eV) |
| --- | --- | --- | --- | --- |
| Co@BNC | -1.24 | -0.37 | 1.03 | 0.58 |
| Co@C | -1.55 | -0.39 | 1.13 | 0.81 |

**Table S6.** The calculated △G values of ORR for Co@BNC and Co@C models at U=0 V.

| Models | △G_1_ (eV) | △G_2_ (eV) | △G_3_ (eV) | △G_4_ (eV) |
| --- | --- | --- | --- | --- |
| Co@BNC | -1.81 | -2.26 | -0.86 | 0.01 |
| Co@C | -2.04 | -2.36 | -0.84 | 0.32 |

**Table S7.** The calculated △G values of ORR for Co@BNC and Co@C models at U=1.23 V.

| Models | △G_1_ (eV) | △G_2_ (eV) | △G_3_ (eV) | △G_4_ (eV) |
| --- | --- | --- | --- | --- |
| Co@BNC | -0.58 | -1.03 | 0.37 | 1.24 |
| Co@C | -0.81 | -1.13 | 0.39 | 1.55 |

**Table S8.** The performance of zinc-air batteries recently reported.

| Catalyst | Current density (mA cm^-2^) | Cycle life  (h) | Voltage gap  (V) | Ref. |
| --- | --- | --- | --- | --- |
| CoBNPCF-800 | 5 | 1706 | 0.808 | This work |
| Co_4_N@CoON/PC GN | 5 | 1350 | 0.65 | ^[8]^ |
| Ni_3_FeN/MnO-CNT | 5 | 200 | 0.73 | ^[15]^ |
| CoS_x_/NCNTs/Ni-2 | 5 | 200 | 0.87 | ^[16]^ |
| Pt/Ar-NCO | 5 | 55 | 0.87 | ^[17]^ |
| NTACo-50Ag(PO) | 5 | 330 | 0.87 | ^[18]^ |
| CNTs-NC-CCC | 10 | 250 | 0.88 | ^[19]^ |
| Fe-SAs@NCTCs | 5 | 650 | 0.76 | ^[20]^ |
| Co-O-ZIF/PANI | 5 | 100 | 0.81 | ^[21]^ |
| Co-N-C@CoNiFe-LDH + Co-N-C | 5 | 950 | 0.77 | ^[22]^ |
| Fe/Fe_3_C@Fe-N_x_-C | 5 | 200 | 0.87 | ^[23]^ |
| CoO/Co*_x_*P | 5 | 202 | 0.86 | ^[24]^ |
| NP-ACSs@NiFe-MOFs | 10 | 450 | 0.79 | ^[25]^ |
| CoNP-PTCOF | 10 | 120 | 0.83 | ^[26]^ |

**References**

[1] G.Kresse, D. Joubert, From ultrasoft pseudopotentials to the projector augmented- wave method. *Phys. Rev. B* **1999**, 59, 1758-177*.*

[2] J. P. Perdew, K. Burke, M. Ernzerhof, Generalized gradient approximation made simple. *Phys. Rev. Lett* **1996**, 77, 3865-3868*.*

[3] S. Grimme, J. Antony, S. Ehrlich, H. Krieg, A consistent and accurate ab initio parametrization of density functional dispersion correction (DFT-D) for the 94 elements H-Pu. *J. Chem. Phys* **2010**, 132, 154104*.*

[4] H. J. Monkhorst, J. D. Pack. Special points for Brillouin-zone integrations. *Phys. Rev. B* **1976**, 13(12): 5188-5192*.*

[5] K. Momma, F. Izumi, VESTA: a three-dimensional visualization system for electronic and structural analysis. *J Appl Crystallogr* **2008**, 41, 653-658.

[6] Y. Yan, B. Wen, M. Liu, H. Lei, J. Yang, S. He, Z. Qu, W. Xia, H. Li, J. Zeng, Orienting Electron Fillings in d Orbitals of Cobalt Single Atoms for Effective Zinc-Air Battery at a Subzero Temperature. *Adv. Funct. Mater.* **2024**, 34, 2316100.

[7] Z. Li, S. Ji, C. Wang, H. Liu, L. Leng, L. Du, J. Gao, M. Qiao, J. H. Horton, Y. Wang, Geometric and Electronic Engineering of Atomically Dispersed Copper-Cobalt Diatomic Sites for Synergistic Promotion of Bifunctional Oxygen Electrocatalysis in Zinc-Air Batteries. *Adv. Mater.* **2023**, 35, 2300905.

[8] M. G. Park, J. Hwang, Y. P. Deng, D. U. Lee, J. Fu, Y. Hu, M. J. Jang, S. M. Choi, R. Feng, G. Jiang, L. Qian, Q. Ma, L. Yang, Y. S. Jun, M. H. Seo, Z. Bai, Z. Chen, Longevous Cycling of Rechargeable Zn-Air Battery Enabled by “Raisin-Bread” Cobalt Oxynitride/Porous Carbon Hybrid Electrocatalysts. *Adv. Mater* **2023**, 36, 2311105.

[9] G. Guan, Y. Liu, F. Li, X. Shi, L. Liu, T. Wang, X. Xu, M. Zhao, J. Ding, H. B. Yang, Atomic Cobalt Metal Centers with Asymmetric N/B-Coordination for Promoting Oxygen Reduction Reaction. *Adv. Funct. Mater.* **2024**, 34, 2408111.

[10] Z. Chen, Y. Zou, H. Chen, K. Zhang, B. Hui, Bamboo-Modulated Helical Carbon Nanotubes for Rechargeable Zn-Air Battery. *Small* **2023**, 20, 2307776.

[11] X. Liu, Z. Wang, J. Wang, T. Tang, C. Li, J. Yu, S. Zhang, C. Deng, Unsaturated cobalt-nitrogen atomic sites in necklace-like hairy fibers towards highly efficient oxygen electrocatalysis for flexible Zn-Air battery. *Energy Storage Mater.* **2024**, 65, 103184.

[12] J. Dong, Y. Pang, D. Bildan, L. Xing, X. Liu, X. Zhuge, Z. Yuan, Y. Ren, D. Liu, W. Lei, K. Luo, Fe_3_Co nanoparticles embedded carbon nanotube complex as effective bifunctional electrocatalysts for rechargeable zinc-air batteries. *Chem. Eng. J.* **2024**, 500, 157309.

[13] Y. Son, K. Min, S. Cheong, B. Lee, S. E. Shim, S. H. Baeck, Innovative Air Cathode with Ni-Doped Cobalt Sulfide in Highly Ordered Macroporous Carbon Matrix for Rechargeable Zn-Air Battery. *Adv. Sci.* **2024**, 11, 2407915.

[14] S. Wang, Z. Li, W. Duan, P. Sun, J. Wang, Q. Liu, L. Zhang, Y. Zhuang, Conjugated polymerized bimetallic phthalocyanine based electrocatalyst with Fe-N_4_/Co-N_4_ dual-sites synergistic effect for zinc-air battery. *J. Energy Chem.* **2023**, 86, 41.

[15] Q. Lu, X. Zou, Y. Bu, K. Liao, W. Zhou, Z. Shao, A Controllable Dual Interface Engineering Concept for Rational Design of Efficient Bifunctional Electrocatalyst for Zinc-Air Batteries. *Small* **2021**, 18, 2105604.

[16] L.-N. Lu, Y.-L. Luo, H.-J. Liu, Y.-X. Chen, K. Xiao, Z.-Q. Liu, Multivalent CoS_x_ coupled with N-doped CNTs/Ni as an advanced oxygen electrocatalyst for zinc-air batteries. *Chem. Eng. J.* **2022**, 427, 132041.

[17] H. Li, L. Hansen, A. Aliyeva, J. Wang, H. Qiu, M. Müller, S. Chen, C. Aktas, L. Kienle, B. Hartke, J. Benedikt, Plasma-engineering of Pt-decorated NiCo_2_O_4_ nanowires with rich oxygen vacancies for enhanced oxygen electrocatalysis and zinc-air battery performance. *Appl. Catal. B-Environ. Energy* **2025**, 361, 124607.

[18] Q. Wang, X. Gao, J. Chen, J. Luo, J. Li, P. Deng, C. You, D. Wu, X. Tian, Silver-decorated amorphous cobalt hydr(oxy)oxide as highly active catalyst for oxygen evolution reaction. *Chem. Eng. J.* **2023**, 462, 142253.

[19] X. Zheng, Y. Qian, H. Gong, W. Shi, J. Yan, W. Wang, X. Guo, J. Zhang, X. Cao, R. Yang, Bridge-linking interfacial engineering of triple carbons for highly efficient and binder-free electrodes toward flexible Zn-air batteries. *Appl. Catal. B-Environ. Energy* **2022**, 319, 121937.

[20] F. Lu, K. Fan, L. Cui, B. Li, Y. Yang, L. Zong, L. Wang, Engineering FeN_4_ active sites onto nitrogen-rich carbon with tubular channels for enhanced oxygen reduction reaction performance. *Appl. Catal. B-Environ. Energy* **2022**, 313, 121464.

[21] H. Lei, S. Yang, Q. Wan, L. Ma, M. S. Javed, S. Tan, Z. Wang, W. Mai, Coordination and interface engineering to boost catalytic property of two-dimensional ZIFs for wearable Zn-air batteries. *J. Energy Chem.* **2022**, 68, 78.

[22] Y. Arafat, Y. Zhong, M. R. Azhar, M. Asif, M. O. Tadé, Z. Shao, CoNiFe‐layered double hydroxide decorated Co-N-C network as a robust bi-functional oxygen electrocatalyst for zinc‐air batteries. *EcoMat* **2023**, 5, 12394.

[23] L. Zong, X. Chen, S. Liu, K. Fan, S. Dou, J. Xu, X. Zhao, W. Zhang, Y. Zhang, W. Wu, F. Lu, L. Cui, X. Jia, Q. Zhang, Y. Yang, J. Zhao, X. Li, Y. Deng, Y. Chen, L. Wang, Ultrafine Fe/Fe_3_C decorated on Fe-N-C as bifunctional oxygen electrocatalysts for efficient Zn-air batteries. *J. Energy Chem.* **2021**, 56, 72.

[24] Y. Niu, M. Xiao, J. Zhu, T. Zeng, J. Li, W. Zhang, D. Su, A. Yu, Z. Chen, A "trimurti" heterostructured hybrid with an intimate CoO/Co_x_P interface as a robust bifunctional air electrode for rechargeable Zn-air batteries. *J. Mater. Chem. A* **2020**, 8, 9177.

[25] L. Zong, P. Li, F. Lu, C. Wang, K. Fan, Z. Li, L. Wang, Assembling Amorphous Metal-Organic Frameworks onto Heteroatom‐Doped Carbon Spheres for Remarkable Bifunctional Oxygen Electrocatalysis. *Adv. Funct. Mater.* **2023**, 33, 2301013.

[26] J. H. Park, C. H. Lee, J. M. Ju, J. H. Lee, J. Seol, S. U. Lee, J. H. Kim, Bifunctional Covalent Organic Framework-Derived Electrocatalysts with Modulated p-Band Centers for Rechargeable Zn-Air Batteries. *Adv. Funct. Mater.* **2021**, 31, 2101727.
